# Decoupling of Carrier Pathways in Au/Cu‐Zn_3_In_2_S_6_ Through Bulk Hole Trapping and Surface Hot Electron Accumulation Enhances Photocatalytic Hydrogen Peroxide Production

**DOI:** 10.1002/adma.202511422

**Published:** 2025-08-18

**Authors:** Xiaowen Ruan, Chunsheng Ding, Jing Leng, Dongxu Jiao, XiangXiang Zhang, Minghua Xu, Depeng Meng, Xiaoqiang Cui, Zhaoke Zheng, Yongfa Zhu, Sai Kishore Ravi

**Affiliations:** ^1^ School of Energy and Environment City University of Hong Kong Tat Chee Avenue, Kowloon Hong Kong SAR 999077 China; ^2^ School of Materials Science and Engineering Key Laboratory of Automobile Materials of MOE Jilin University Changchun 130012 China; ^3^ State Key Laboratory of Chemical Reaction Dynamics Dalian Institute of Chemical Physics Chinese Academy of Sciences Dalian 116023 China; ^4^ State Key Laboratory of Crystal Materials Shandong University Jinan 250100 China; ^5^ Department of Chemistry Tsinghua University Beijing 100084 China

**Keywords:** artificial photosynthesis, H_2_O_2_ production, plasmonic nanoparticles, reactive oxygen species, spatial decoupling

## Abstract

Artificial photosynthesis offers a sustainable route to H_2_O_2_ production but is hindered by charge recombination and non‐selective reactive species generation, resulting in parasitic reactions that reduce selectivity and yield. Here, Au‐Cu co‐modified Zn_3_In_2_S_6_ (Au/Cu‐d/ZIS) is presented, a catalyst that spatially decouples charge carriers across bulk and surface sites, suppressing recombination and stabilizing intermediates for photocatalytic oxygen reduction. Cu doping introduces trap states that localize holes in the bulk and improve the separation and transportation of bulk photogenerated carriers. Plasmonic Au nanoparticles drive surface hot electron accumulation and further contribute to the oxygen reduction reaction. The optimized catalyst achieves an H_2_O_2_ evolution rate of 94.2 µmol g^−1^ min^−1^ using pure water without any sacrificial agents, outperforming pristine Zn_3_In_2_S_6_ by nearly threefold. DRIFTS identifies stabilized oxygenated species on the catalyst surface, and DFT calculations demonstrate that Cu trap states lower energy barriers for •O_2_^−^ formation, while Au NPs enhance the charge transfer and ORR reaction between ZIS and O_2_. The catalyst maintains stability and reusability, producing 2300 µmol g^−1^ of H_2_O_2_ under natural sunlight over 4 h with consistent performance across multiple cycles. Furthermore, it is successfully applied for bacterial sterilization and pharmaceutical pollutant degradation, demonstrating its potential for environmental remediation.

## Graphical abstract

We design and implement Au‐Cu co‐modified Zn_3_In_2_S_6_ catalyst with spatially decoupling charge carriers across bulk and surface sites, which effectively suppresses carrier recombination, drives surface hot electron accumulation and stabilizes intermediates for photocatalytic oxygen reduction. The optimized catalyst achieves an H_2_O_2_ evolution rate of 94.2 µmol g^−1^ min^−1^ using pure water without any sacrificial agents, far exceeding most reported photocatalysts.

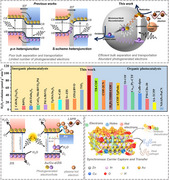

## 1 Introduction

Hydrogen peroxide (H_2_O_2_) is a versatile and valuable chemical with widespread applications in disinfection, environmental remediation, clean fuel production, and organic synthesis.^[^ ^1^, ^2^, ^3^, ^4^ ^]^ However, its conventional industrial production methods, including the anthraquinone process, the direct H_2_–O_2_ reaction, and electrocatalytic oxygen reduction, face critical challenges such as high energy demands, harsh reaction conditions, and substantial chemical waste.^[^ ^5^, ^6^, ^7^, ^8^ ^]^ These limitations necessitate the development of innovative and sustainable approaches for efficient H_2_O_2_ production under ambient conditions. Artificial photosynthesis of H_2_O_2_ from oxygen and water, which utilizes sunlight as the sole energy source to drive mild chemical conversions, represents a promising solution to address these challenges.^[^ ^9^, ^10^, ^11^, ^12^, ^13^, ^14^ ^]^

Artificial photosynthesis couples sunlight with oxygen and water to drive sustainable H_2_O_2_ production through two key half‐reactions: the two‐electron oxygen reduction (O_2_ + 2H^+^ + 2e^−^ → H_2_O_2_, 0.68 V vs RHE) and water oxidation (2H_2_O +2h^+^→ H_2_O_2_ + 2H^+^, 1.76 V vs RHE).^[^ ^15^, ^16^ ^]^ Numerous photocatalysts, including metal‐organic frameworks (MOFs), graphitic carbon nitride (g‐C_3_N_4_), metal oxides (TiO_2_), and covalent organic frameworks (COFs), have been studied to facilitate these reactions.^[^ ^17^, ^18^, ^19^, ^20^ ^]^ However, their application is often limited by poor visible‐light absorption, inappropriate bandgaps, slow reaction kinetics, and high recombination rates of photogenerated electron‐hole pairs, which collectively reduce H_2_O_2_ selectivity and yield.

To address the limitations of photocatalysts, strategies such as the introduction of sacrificial agents and heterostructure construction have been employed to enhance charge separation and reduce recombination. Sacrificial agents act as electron or hole scavengers to drive reactions, while heterostructures create energy level mismatches at interfaces to promote charge transfer.^[^ ^21^, ^22^, ^23^ ^]^ However, these approaches primarily target surface‐active sites and fail to address recombination in the bulk crystal, where over 90% of photogenerated carriers recombine before reaching the surface. This bulk recombination significantly limits the utilization of photogenerated carriers and reduces overall efficiency. Furthermore, the use of sacrificial agents complicates mechanistic investigations, reduces intrinsic catalytic efficiency, and introduces additional challenges in product purification. As a result, there is a critical need to develop strategies that enable spatially controlled charge separation across bulk and surface regions of single photocatalysts, thereby maximizing the effective use of photogenerated carriers for H_2_O_2_ production under ambient conditions and without sacrificial agents.

Zn_3_In_2_S_6_, a typical n‐type ternary chalcogenide compound, has emerged as a promising photocatalyst due to its suitable band positions for redox reactions, favorable conduction and valence band edges, and high redox potential. These properties make it well‐suited for artificial photosynthesis of H_2_O_2_.^[^ ^24^, ^25^, ^26^ ^]^ However, single‐phase Zn_3_In_2_S_6_ suffers from high bulk charge recombination, where a majority of photogenerated carriers are lost during transport, as well as insufficient surface‐active sites for catalytic reactions. Heteroatom doping has been widely explored to mitigate these issues by introducing intermediate trap states within the bandgap. These trap states facilitate bulk charge separation, prolong carrier lifetimes, suppress recombination, and extend light absorption range.^[^ ^27^, ^28^, ^29^, ^30^, ^31^ ^]^ Nevertheless, excessive doping can disrupt the crystal structure, forming recombination centers that degrade photocatalytic performance. To complement bulk charge separation, surface modification with plasmonic nanoparticles, such as Au, can be employed to enhance surface electron density through localized surface plasmon resonance (LSPR).^[^ ^32^, ^33^, ^34^, ^35^, ^36^ ^]^ LSPR not only broadens the absorption spectrum into the visible and near‐infrared regions but also creates high‐energy “hot electrons” that promote oxygen reduction reactions.^[^ ^33^, ^37^, ^38^ ^]^ When gold nanoparticles are irradiated with light, the LSPR effect induces localized charge redistributions, increasing the electron density on one side of the nanoparticle and reducing it on the other. This augmentation in surface charge density enhances the rate and selectivity of catalytic reactions. Since photocatalytic H_2_O_2_ production primarily relies on electron‐driven reduction reactions, having a surplus of electrons is particularly advantageous. Therefore, coupling heteroatom doping to improve bulk charge separation with plasmonic surface modification represents a synergistic strategy to address the dual challenges of charge recombination and limited surface activity.

Given the challenges of high bulk recombination and limited surface‐active sites in single‐phase Zn_3_In_2_S_6_, we propose a catalyst design combining heteroatom doping and plasmonic surface modification to achieve spatial decoupling of carrier pathways. Cu doping introduces intermediate trap states within the bulk, effectively localizing holes and reducing bulk recombination, while Au nanoparticles enhance surface electron density via LSPR effect. This approach not only improves bulk charge separation but also facilitates the accumulation of high‐energy hot electrons on the surface, critical for oxygen reduction to H_2_O_2_. By stabilizing reactive oxygen intermediates and enhancing charge transfer dynamics, this dual modification strategy aims to maximize the efficiency of Zn_3_In_2_S_6_ for artificial photosynthesis of H_2_O_2_ under ambient conditions. The synergistic interplay between Cu‐induced bulk modifications and Au‐driven surface plasmonic effects establishes a robust pathway for overcoming the intrinsic limitations of photocatalysts, enabling efficient, selective, and sustainable H_2_O_2_ production.

To validate the effectiveness of this approach, we designed a catalyst design composed of Cu‐doped Zn_3_In_2_S_6_ with surface‐anchored Au nanoparticles (denoted as: Au/Cu‐d/ZIS). In situ X‐ray photoelectron spectroscopy (XPS), femtosecond transient absorption spectroscopy, and theoretical analyses confirmed an efficient charge transfer and effective extension of carrier lifetime induced by the synergistic effect of Cu doping and Au LSPR. The optimized Au/Cu‐d/ZIS catalyst exhibited an enhanced H_2_O_2_ evolution rate of 94.2 µmol g^−1^ min^−1^ using pure water without any sacrificial agents, surpassing most previously reported photocatalysts. Free radical trapping experiments, electron spin resonance (ESR) spectroscopy, and in situ diffuse reflectance infrared Fourier transform spectroscopy (DRIFTS) confirmed the roles of reactive oxygen intermediates in H_2_O_2_ production. The system exhibited robust stability and reusability, with the generated H_2_O_2_ effectively applied in bacterial sterilization and pharmaceutical pollutant degradation. These findings highlight the potential of decoupling carrier pathways across bulk and surface regions in overcoming the limitations of traditional photocatalysts and pave the way for sustainable artificial photosynthetic systems.

## 2 Results and Discussion

### 2.1 Surface Morphology and Chemical Composition

**Figure 1:**
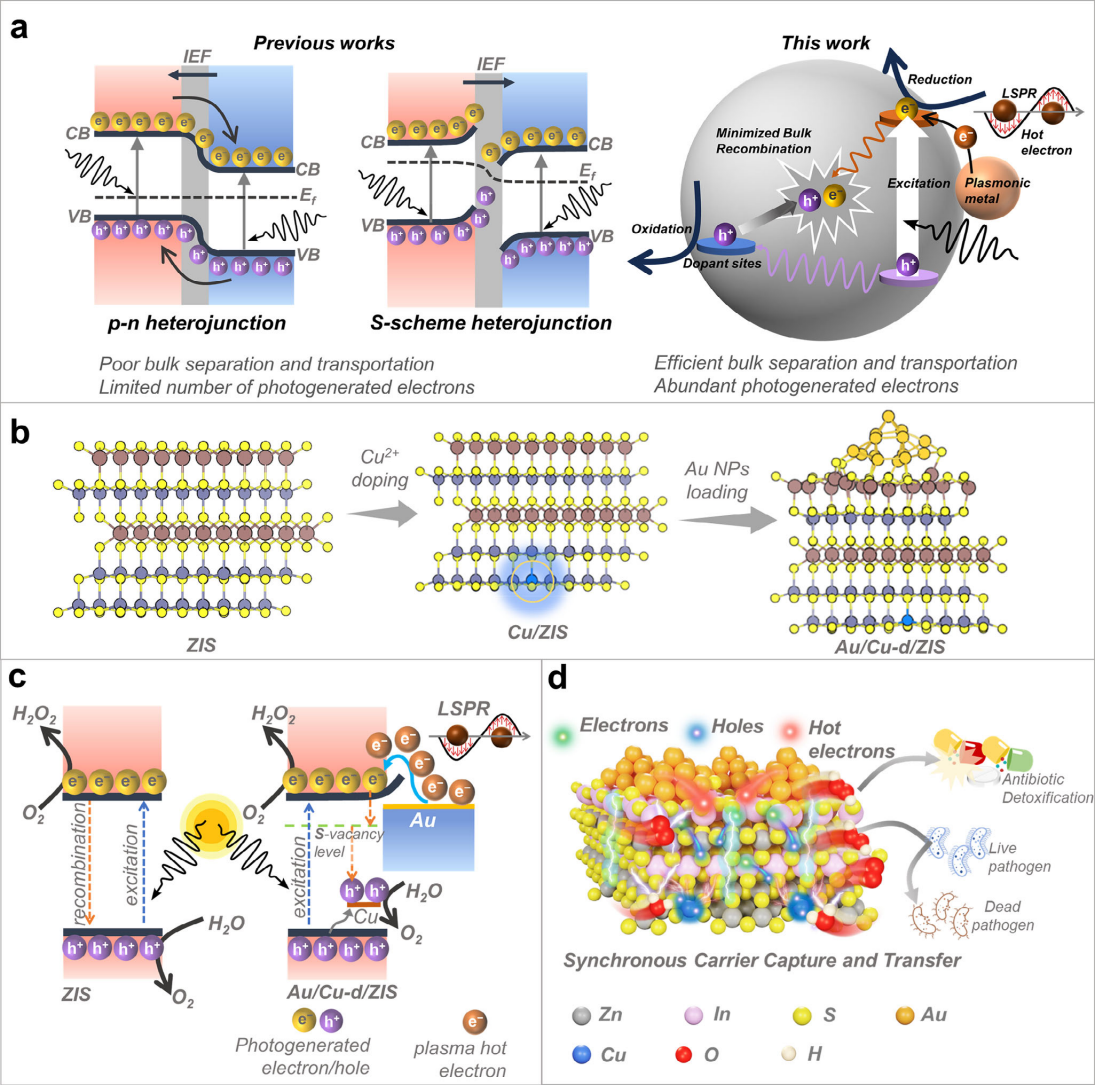
a) Schematic illustration comparing p‐n junctions, S‐scheme heterojunctions, and the proposed Au/Cu‐d/ZIS system in artificial photosynthesis. b) Schematic representation of the material design for Au/Cu‐d/ZIS. c) Band structure illustrating the effects of Cu doping and Au plasmonic nanoparticles on charge separation and energy alignment. d) Visualization of the photogenerated carrier separation and transport in Au/Cu‐d/ZIS, highlighting its efficiency in photocatalytic H_2_O_2_ production and potential environmental applications.

**Figure** **1**a presents a schematic illustration of the proposed Au/Cu‐d/ZIS system, compared with previously reported heterojunction‐based artificial photosynthetic systems. Traditional systems, such as p‐n junctions and S‐scheme heterojunctions, primarily focus on the surface separation and transport of photogenerated carriers. However, these approaches are limited by poor bulk charge separation, inefficient carrier transport, and an insufficient number of photogenerated electrons, which collectively constrain their photocatalytic efficiency. Our work addresses these limitations through the combined strategy of elemental doping and LSPR effect (Figure 1a). Specifically, Cu^2+^ cations were chosen as dopants to substitute Zn^2+^ in Zn_3_In_2_S_6_, while Au nanoparticles were loaded onto the catalyst surface to form the Au/Cu‐d/ZIS system (Figure 1b). This design achieves enhanced bulk/surface separation and efficient carrier transport, while simultaneously generating abundant photogenerated electrons, as schematically illustrated in Figure 1c,d. These combined features are highly advantageous for boosting the photocatalytic production of H_2_O_2_ under ambient conditions.

**Figure 2:**
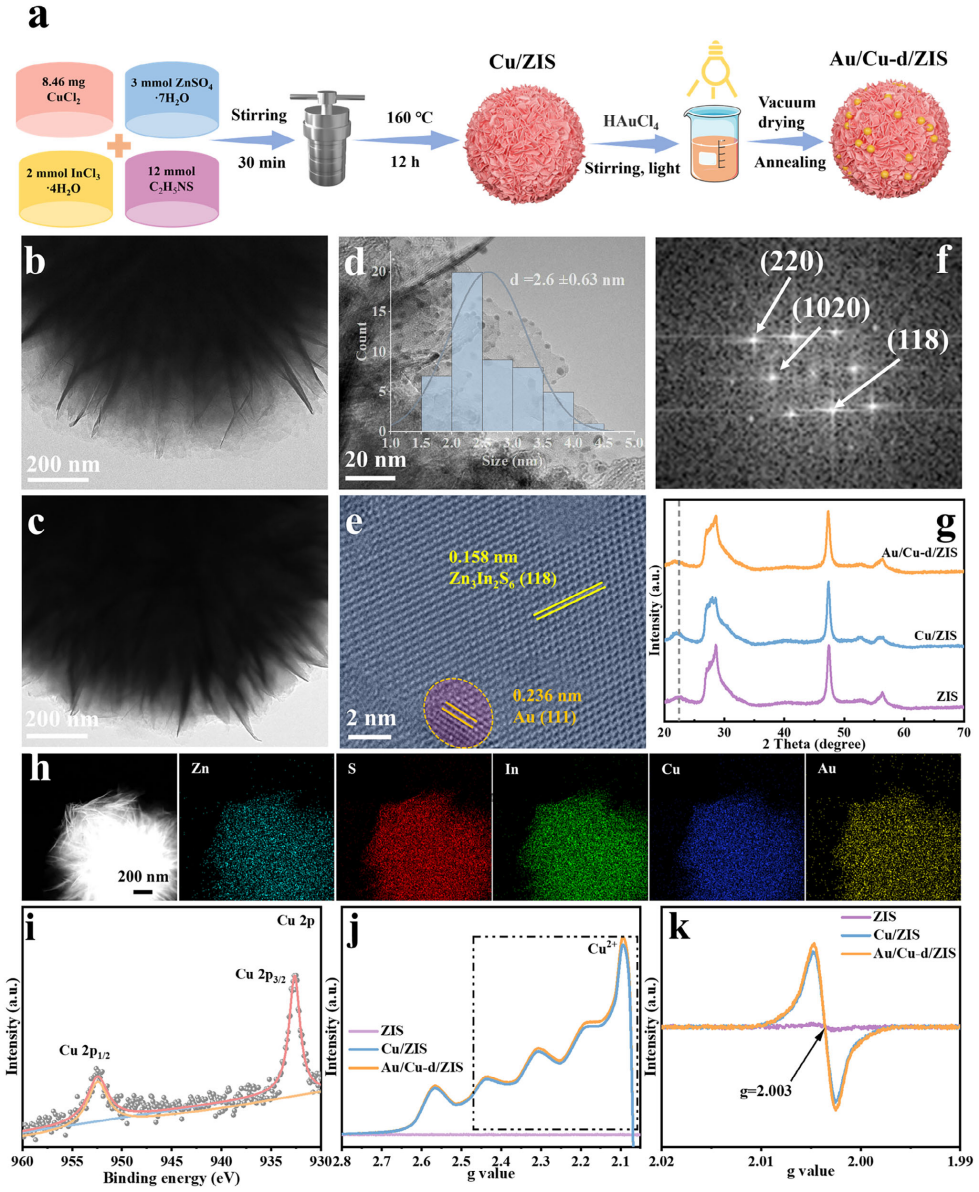
a) Schematic of the Au/Cu‐d/ZIS synthetic route. TEM images of b) pristine ZIS, c) Cu/ZIS, and d) Au/Cu‐d/ZIS (Inset: Size distribution of Au NPs). e) HRTEM and f) FFT images of Au/Cu‐d/ZIS. g) XRD spectra of pristine ZIS, Cu/ZIS, and Au/Cu‐d/ZIS. h) Elemental mapping images and i) Cu 2*p* XPS spectra of Au/Cu‐d/ZIS. j,k) Low‐temperature ESR spectra of pristine ZIS, Cu/ZIS, and Au/Cu‐d/ZIS.

The synthetic route of Au/Cu‐d/ZIS is shown in **Figure** **2**a. The morphology and structure of the prepared catalysts are firstly characterized by scanning electron microscopy (SEM) and transmission electron microscopy (TEM). Similar to pristine ZIS, the Cu/ZIS catalyst exhibits a sea urchin‐like structure composed of abundant nanosheets, indicating that Cu doping preserves the fundamental structure of ZIS (Figure 2b,c; Figures S1–S4, Supporting Information). Furthermore, TEM analysis confirmed that Au nanoparticles were firmly anchored on the surface of the Au/Cu‐d/ZIS catalyst (Figure 2d). HRTEM imaging revealed crystal facets with spacings of 0.158 and 0.236 nm, corresponding to the (118) plane of the hexagonal ZIS phase and the (111) plane of Au nanoparticles, respectively (Figure 2e). The structure of the Au/Cu‐d/ZIS catalyst was further validated by fast Fourier transform (FFT) analysis (Figure 2f). X‐ray diffraction (XRD) analysis confirmed that no additional impurity phases were introduced during the modification process, and the basic structure of ZIS was retained, consistent with the morphological observations (Figure 2g). A slight shift in XRD peaks was observed in the Cu/ZIS and Au/Cu‐d/ZIS catalysts, attributed to the lattice contraction caused by the substitution of Zn^2+^ with the smaller Cu^2+^ ions (Figure 2g).^[^ ^27^ ^]^ Elemental mapping demonstrated the homogeneous distribution of Zn, In, S, Cu, and Au throughout the Au/Cu‐d/ZIS catalyst (Figure 2h). The presence and oxidation state of Cu species were further confirmed through X‐ray photoelectron spectroscopy (XPS). The Cu 2*p* spectra of the Au/Cu‐d/ZIS catalyst showed two distinct peaks at binding energies of 932.68 and 952.48 eV, which are ascribed to Cu 2*p*_3/2_ and Cu 2*p*_1/2_ of Cu^2+^, respectively, whose result is consistent with that of previous related research (Figure 2i).^[^ ^39^ ^]^ Electron spin resonance (ESR) spectroscopy was employed to investigate the valence states of the Cu dopant and vacancy properties. A characteristic signal with a g‐value of 2.08 was detected in both Cu/ZIS and Au/Cu‐d/ZIS, indicating the presence of Cu^2+^, while no discernible signal was observed in pristine ZIS (Figure 2j).^[^ ^40^ ^]^ Additionally, both Cu/ZIS and Au/Cu‐d/ZIS displayed an apparent ESR signal at a g‐value of 2.003, attributed to sulfur vacancies bound to unpaired electrons, further confirming the defect‐rich structure (Figure 2k; Figure S5, Supporting Information).

The appearance of S vacancies can be attributed to the following explanation: Specifically, in doping engineering strategy, when foreign atoms occupy the host lattice, Jahn–Teller distortion occurs due to differences in atomic radius and coordination numbers.^[^ ^41^, ^42^ ^]^ Therefore, after Cu doping into ZIS, influenced by Jahn–Teller distortion, adjacent S atoms escape from the lattice, generating adaptive S vacancies as a stress response.^[^ ^27^ ^]^ Meanwhile, distortion can maintain thermodynamic stability to a certain extent. Therefore, in Cu/ZIS, Cu exists in a divalent form and replaces the position of Zn, accompanied by the formation of S vacancies.

### 2.2 Photocatalytic Performance of Catalysts

**Figure 3:**
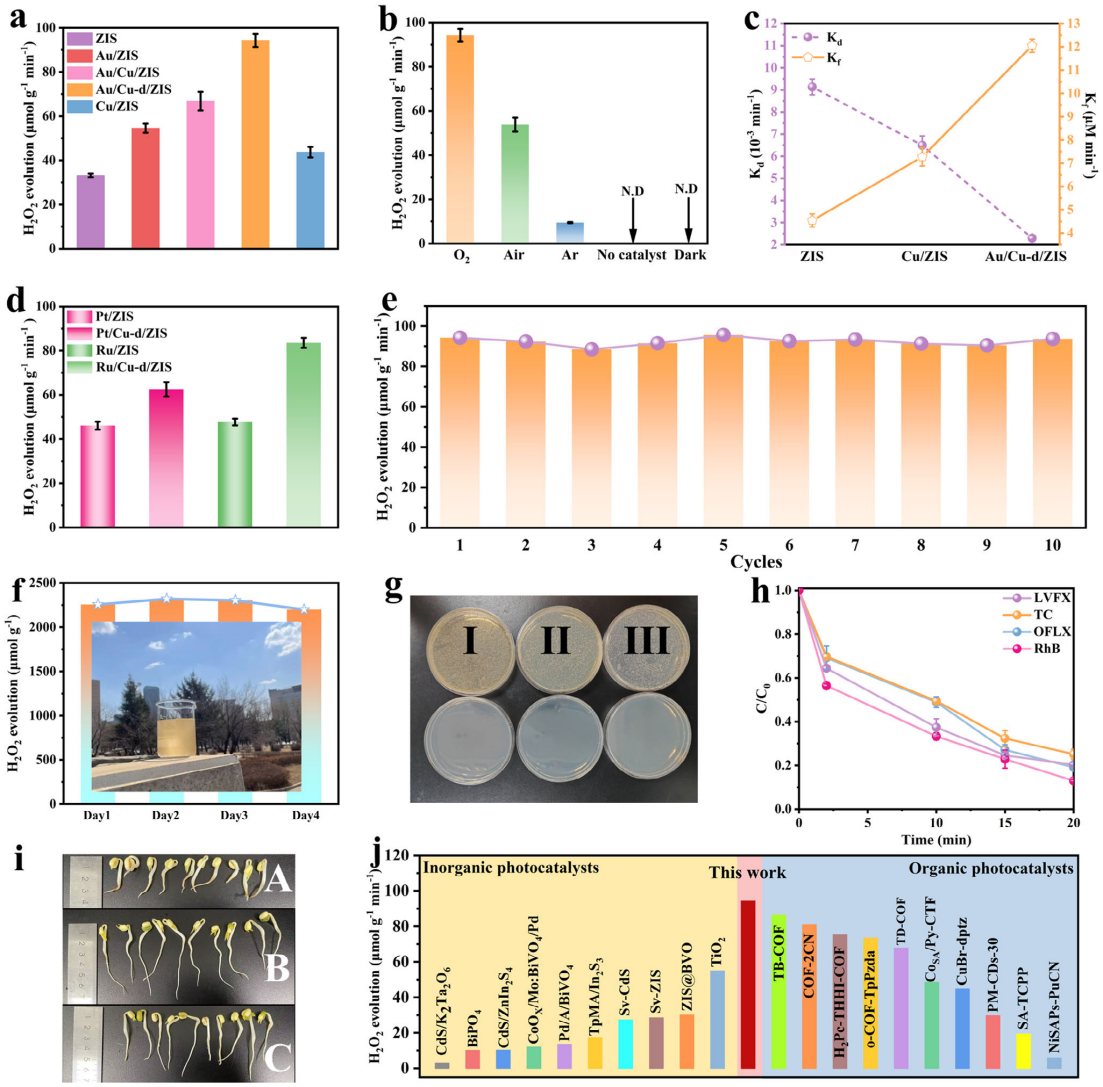
a) Photocatalytic H_2_O_2_ evolution rates of the as‐prepared photocatalysts. b) Photocatalytic H_2_O_2_ evolution rate of the Au/Cu‐d/ZIS catalyst under various conditions. c) H_2_O_2_ formation rate constant (*K*_f_) and decomposition rate constant (*K*_d_) for pristine ZIS, Cu/ZIS, and Au/Cu‐d/ZIS catalysts. d) Photocatalytic H_2_O_2_ production rates of Pt/ZIS, Pt/Cu‐d/ZIS, Ru/ZIS, and Ru/Cu‐d/ZIS catalysts. e) Cycling experiments demonstrating the stability of the Au/Cu‐d/ZIS catalyst. f) H_2_O_2_ evolution using the Au/Cu‐d/ZIS catalyst under natural sunlight irradiation over four non‐consecutive sunny days (10:30 AM to 2:30 PM). g) Antimicrobial properties of photocatalytically produced H_2_O_2_ against I: Rhodospirillum, II: Alcaligenes, and III: Escherichia coli. h) In situ photocatalytic H_2_O_2_ production for the degradation of antibiotics and Rhodamine B (RhB). i) Comparison of mung bean sprout rhizome lengths cultured under different conditions: A (TC solution), B (ultrapure water), and C (degraded TC solution). j) Comparison of photocatalytic H_2_O_2_ production rates achieved by the Au/Cu‐d/ZIS catalyst with previously reported organic and inorganic photocatalysts.

The artificial photosynthesis of H_2_O_2_ was investigated in pure water without the use of sacrificial agents. The optimized Au/Cu‐d/ZIS catalyst demonstrated the highest H_2_O_2_ evolution rate of 94.2 µmol g^−1^ min^−1^, significantly outperforming pristine ZIS (33.2 µmol g^−1^ min^−1^), Cu/ZIS (43.7 µmol g^−1^ min^−1^), Au/ZIS (54.6 µmol g^−1^ min^−1^) and Au/Cu/ZIS (66.8 µmol g^−1^ min^−1^), respectively (**Figure** **3**a). These results indicate that the co‐modification of Cu and Au, along with the presence of sulfur vacancies, synergistically enhances the photocatalytic H_2_O_2_ evolution performance (Figure S6, Supporting Information). Control experiments revealed that H_2_O_2_ production was completely inhibited in the absence of light or photocatalysts. Under O_2_ injection conditions, the H_2_O_2_ evolution rate reached 94.2 µmol g^−1^ min^−1^ and 36.8 µmol g^−1^ min^−1^ under UV‐vis and visible light irradiation, respectively, further highlighting the catalyst's optimal performance (Figure 3b; Figure S7, Supporting Information). To gain deeper insights into the H_2_O_2_ production dynamics, the H_2_O_2_ generation rate constant (K_f_, µm min^−1^) and decomposition rate constants (K_d_, min^−1^) were calculated for pristine ZIS, Cu/ZIS, and Au/Cu‐d/ZIS catalysts. The Au/Cu‐d/ZIS catalyst exhibited the highest K_f_ and lowest K_d_ values, indicating an improved capability to promote H_2_O_2_ production while minimizing its decomposition (Figure 3c; Figures S8 and S9, Supporting Information). Further control experiments including H_2_O_2_ decomposition comparison by the prepared catalyst under dark condition were investigated. The results showed that no H_2_O_2_ was generated in the dark and the decomposition of H_2_O_2_ was almost negligible, indicating that the generation and decomposition of H_2_O_2_ are both driven by photoelectrons and holes (Figure 3b; Figure S10, Supporting Information). Additionally, substituting Au with Pt or Ru to form Pt/Cu‐d/ZIS and Ru/Cu‐d/ZIS demonstrated similarly enhanced H_2_O_2_ evolution performance (Figure 3d). Cycling experiments and structural characterizations before and after the reactions confirmed the stability and reusability of the Au/Cu‐d/ZIS catalyst (Figure 3e; Figures S11–S16, Supporting Information). Under natural sunlight irradiation, the Au/Cu‐d/ZIS catalyst achieved ≈2300 µmol g^−1^ of H_2_O_2_ in 4 h, maintaining similar yields over 4 consecutive sunny days (Figure 3f). The generated H_2_O_2_ was further applied for environmental remediation. Direct bacterial inactivation using the photocatalytically produced H_2_O_2_ achieved 100% efficiency, and effective degradation of pharmaceutical pollutants, including levofloxacin (LVFX), ofloxacin (OFL), Rhodamine B (RhB), and tetracycline hydrochloride (TC), was demonstrated (Figure 3g,h). Notably, mung bean sprouts cultured in the degraded TC solution exhibited similar growth conditions to those grown in ultrapure water, confirming the detoxification efficacy of the system (Figure 3i). These findings indicate that the optimized Au/Cu‐d/ZIS photocatalytic system demonstrates superior performance in sterilization and detoxification applications. Moreover, the achieved H_2_O_2_ evolution rate surpasses most previously reported organic and inorganic photocatalysts (Figure 3j).^[^ ^43^, ^44^, ^45^, ^46^, ^47^, ^48^, ^49^, ^50^, ^51^, ^52^, ^53^, ^54^, ^55^, ^56^, ^57^, ^58^, ^59^, ^60^, ^61^, ^62^ ^]^

### 2.3 Chemical Composition and Carrier Transportation

**Figure 4:**
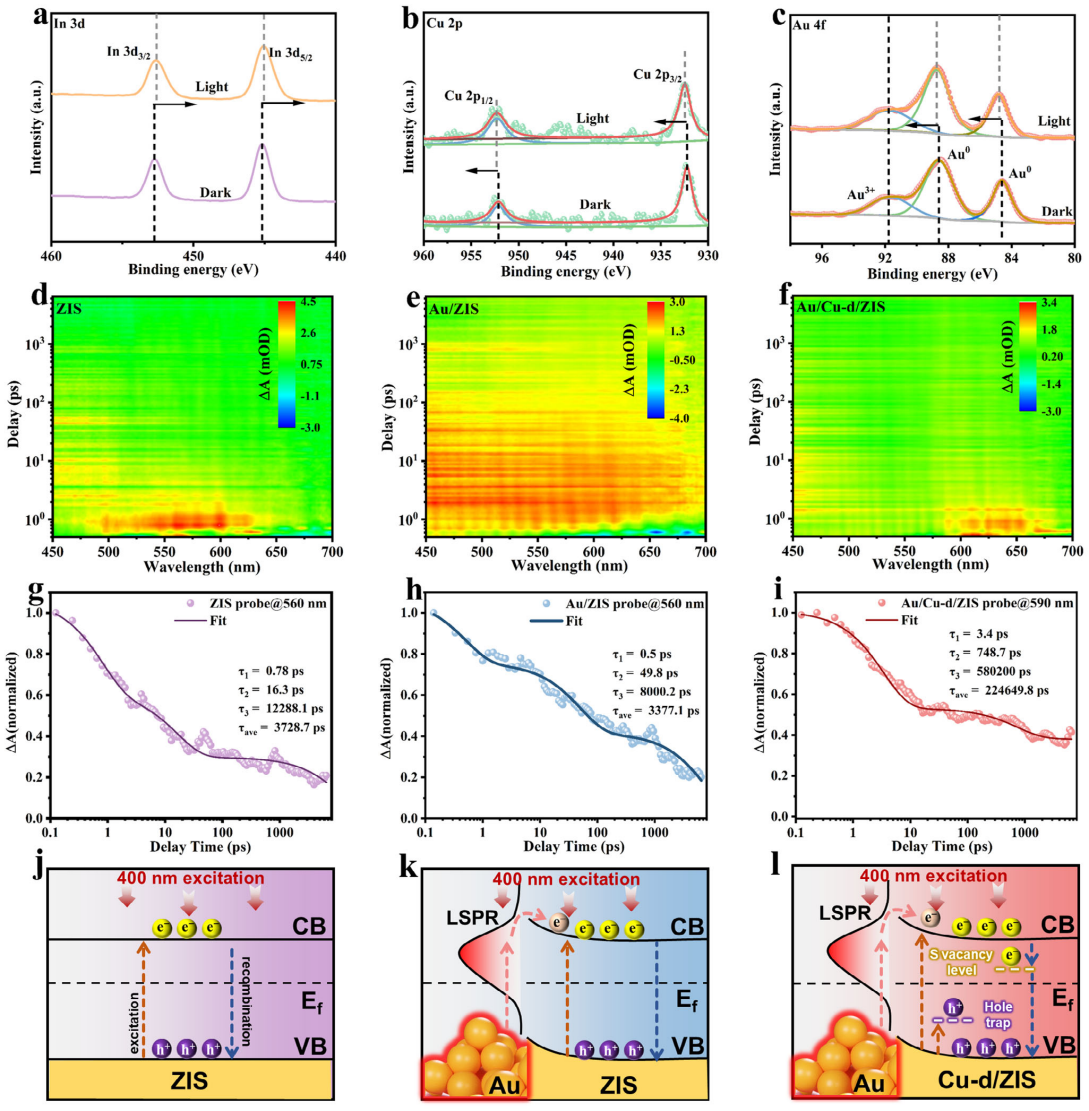
In situ XPS spectra of a) In 3d, b) Cu 2p, and c) Au 4f under dark and irradiation of Au/Cu‐d/ZIS catalyst, respectively. Transient absorption spectra of d) ZIS, e) Au/ZIS, and f) Au/Cu‐d/ZIS. Normalized transient absorption kinetics for g) ZIS, h) Au/ZIS, and i) Au/Cu‐d/ZIS after 400 nm laser excitation. j–l) Schematic illustration of photogenerated electrons transfer and hot electrons injection under 400 nm excitation.

Theoretically, as a typical layered material, ZIS has a distinct internal electric field distribution. When exposed to light irradiation, photo‐generated electrons and holes can be separated and transferred to different layers. Photo‐generated electrons tend to accumulate at in site while photo‐generated holes will accumulate at Zn site.^[^ ^63^, ^64^ ^]^ Therefore, when Cu‐doped ZIS replaces Zn sites, it is reasonable for Cu sites to act as hole acceptors. On the other hand, although Cu and Zn have the same valence, due to electronegativity differences (Cu: 1.90, Zn: 1.65), introducing Cu into ZIS will inevitably lead to a redistribution of interface charges. To maintain charge balance, Cu, which has a greater electronegativity, will attract more electrons than Zn, ultimately forming an electron‐rich center at the Cu sites. When illumination is applied, photogenerated electrons and holes are excited and separated. According to the principle of opposite charges attracting each other, the photo‐generated holes are more likely to transfer to the electron‐rich Cu sites. To further investigate the carrier transportation and redox sites, in situ XPS test was subsequently tested.^[^ ^65^ ^]^ Specifically, under illumination, the Au 4f and Cu 2p peaks in Au/Cu‐d/ZIS shifted toward higher binding energies, while the In 3d and S 2p peaks shifted to lower binding energies (**Figure** **4**a–c; Figures S17 and S18, Supporting Information). These shifts indicate that ZIS acts as an electron acceptor, while Au and Cu serve as electron donors and hole acceptors, respectively. This observation is also detected by in‐situ light irradiation XPS with oxygen injection and confirms the potential for directional transportation of photogenerated carriers in the Au/Cu‐d/ZIS catalyst (Figure S19, Supporting Information). Ex situ XPS further confirmed the successful construction of the catalyst by analyzing the electronic states and chemical composition of pristine ZIS, Cu/ZIS, and Au/Cu‐d/ZIS (Figures S20 and S21, Supporting Information). Similar trends are also detected in Pt/Cu‐d/ZIS and Ru/Cu‐d/ZIS catalyst, and it suggests that this kind of modification strategy is universal and feasible (Figures S22 and S23, Supporting Information). Femtosecond transient absorption (fs‐TA) spectra were recorded to examine the enhanced carrier transfer dynamics. Pristine ZIS exhibited significant absorption within the 450–‐600 nm range, while the modified Au/ZIS and Au/Cu‐d/ZIS catalysts displayed broadened light absorption ranges under photoexcitation (Figure 4d–f). Time‐dependent intensity decay trends in the TA spectra and the corresponding decay kinetics were analyzed at 400 nm (Figure 4g–i; Figures S24–S26, Supporting Information). Upon comparing the exciton bleaching kinetics of pristine ZIS, it becomes evident that the Au/ZIS sample exhibits a prolonged lifetime, indicating that hot electrons generated in the Au cores will inject into ZIS. Meanwhile, introducing Cu doping and S vacancy can strongly trap the holes and electrons at the valence band maximum and conduction band maximum, respectively, thereby prolonging the lifetime of photogenerated carriers and facilitating their participation in hydrogen peroxide evolution reactions. At this time, the photo‐electrons generated from ZIS and hot electrons generated by Au will accumulate on the S vacancy site and S vacancy acts as the main oxygen reduction reaction site. Simultaneously, the photogenerated holes will be effectively captured by Cu site, thus well achieving the efficient carriers’ transportation through bulk hole trapping and surface hot electron accumulation (Figure 4j–l). The average carrier lifetimes (τ_av_) of ZIS, Au/ZIS, and Au/Cu‐d/ZIS were calculated as 3728.7, 3377.1, and 224649.8 ps, respectively. The significantly extended carrier lifetime in Au/Cu‐d/ZIS underscores its superior charge transfer efficiency and reduced recombination of photogenerated electron‐hole pairs. Transient photocurrent response and electrochemical impedance spectroscopy (EIS) measurements were performed to evaluate the separation and transport of photogenerated carriers. The Au/Cu‐d/ZIS catalyst exhibited a notably enhanced photocurrent response and reduced charge transfer resistance compared to pristine ZIS and Cu/ZIS (Figures S27 and S28, Supporting Information), indicating improved charge transfer efficiency. Negative/positive sweep LSV current curves under light irradiation and steady‐state surface photovoltage (SPV) reveal that Cu doping is beneficial for capturing photogenerated holes and enhancing the oxidation performance while Au can promote the concentration of photogenerated electrons and reduction reaction kinetics (Figures S29 and S30, Supporting Information). Additionally, the photoluminescence (PL) spectra showed that Au/Cu‐d/ZIS had the lowest PL intensity and shortest time‐resolved PL (TRPL) lifetime, confirming that the introduction of Cu and Au effectively suppressed carrier recombination, allowing more photogenerated carriers to be separated and transferred efficiently (Figures S31 and S32, Supporting Information).

To investigate the light absorption property and bandgap of as‐prepared photocatalysts, UV–vis diffuse reflectance spectra (UV–vis DRS) and valence band XPS spectra (VB‐XPS) are measured. The optical absorption edges of pristine ZIS and Au/ZIS mainly range from 400 to 500 nm while it in Cu/ZIS and Au/Cu‐d/ZIS catalyst range from 500 to 600 nm (Figure S33a, Supporting Information). This suggests that modification of Au and Cu can enhance the light absorption ability of ZIS. The corresponding optical band gaps of ZIS and Cu/ZIS are calculated to be 2.81 and 2.69 eV, respectively (Figure S33b, Supporting Information). According to the equation E_vb_ = Φ + E_vb‐xps_ ‐ 4.44 eV (where Φ is the work function of the XPS instrument, 4.40 eV), the positions of the valence bands (VBs) of ZIS and Cu/ZIS is 1.62 and 1.48 eV, respectively (Figure S34 Supporting Information).^[^ ^24^ ^]^ Based on the tested energy gap between valence band edge and Fermi level versus RHE, the band positions of ZIS and Cu/ZIS are proposed, and they can meet the theoretical potential requirements for H_2_O_2_ production by oxygen reduction reaction (Figure S35, Supporting Information).

**Figure 5:**
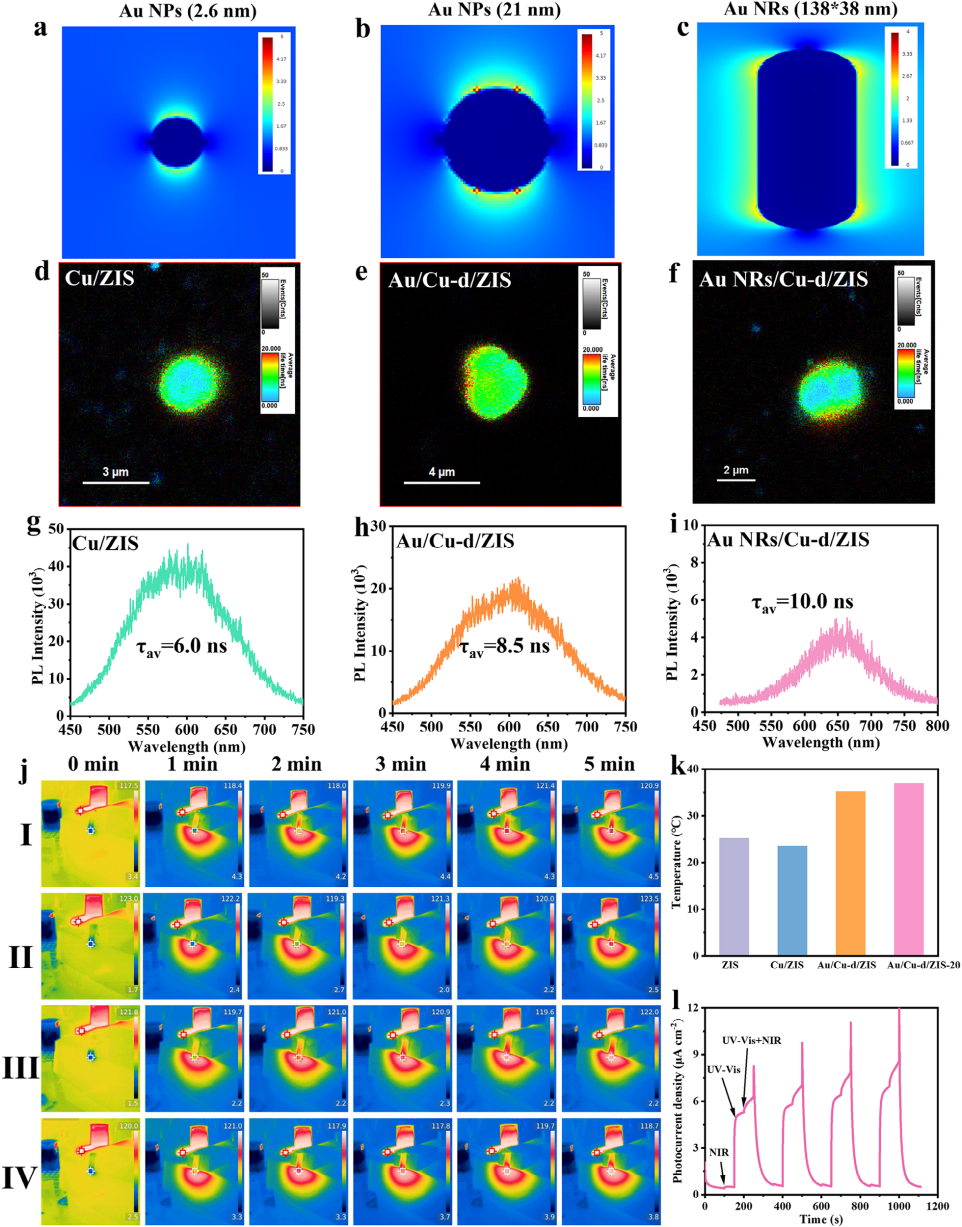
a–c) Electric field distribution from the FDTD for different Au. Single‐particle d–f) PL image and corresponding spectra (g‐i) of Cu/ZIS, Au/Cu‐d/ZIS, and Au NRs/Cu‐d/ZIS. j,k) Temperature change over time of I: ZIS, II: Cu/ZIS, III: Au/Cu‐d/ZIS, and IV: Au/Cu‐d/ZIS‐20 catalysts under infrared light irradiation. l) Transient photocurrent curves of Au/Cu‐d/ZIS under different illumination conditions.

To have a deep understanding of LSPR effect, we further employed the Finite Difference Time Domain (FDTD) method to calculate the extinction, absorption, and scattering spectra of different types of Au. As shown in **Figure** **5**a–c, plasmonic effect is observed in small size Au NPs and the local electric field effect will be continuously enhanced with increasing the Au size. This effect can promote the hot electron generation, accelerate the carrier transportation, and provide abundant active carriers involved in subsequent catalytic processes (Figures S36 and S37, Supporting Information). Single‐particle fluorescence spectroscopy is further tested to investigate the local charge distribution and carrier transportation in LSPR‐mediated photocatalysts (Figure 5d–i). Pristine Cu/ZIS catalyst exhibits a high PL intensity while the significant deceased intensity of Cu/ZIS with Au NPs and increased average carrier lifetime are observed, which suggest that an efficient electron transfer exists between Cu/ZIS and Au. Thermal imaging photographs are used to record the temperature changes and photothermal conversion performance of the as‐prepared photocatalyst under infrared light irradiation (Figure 5j). Compared with pristine ZIS and Cu/ZIS, the temperature changes in Au/Cu‐d/ZIS and Au/Cu‐d/ZIS‐20 were more significant within 5 min, further validating the successful LSPR effect introduction (Figure 5k). Furthermore, we have tested the transient photocurrent responses of Au/Cu‐d/ZIS and the result shows that the photocurrent response exposed to both ultraviolet‐visible and near‐infrared light is enhanced compared to it under ultraviolet‐visible light alone (Figure 5l). The sudden surge in photocurrent response upon turning off the light further indicates that the hot electrons generated by Au NPs under LSPR cause an imbalance between internal and external carriers in Cu‐d/ZIS. Based on the above discussion, the optimized Au/Cu‐d/ZIS catalyst with surface plasmon resonance (LSPR) effect can effectively promote the separation and transfer of surface and bulk carriers and be beneficial for photocatalytic reactions.

### 2.4 Radical Trapping Experiments and Reaction Mechanism

**Figure 6:**
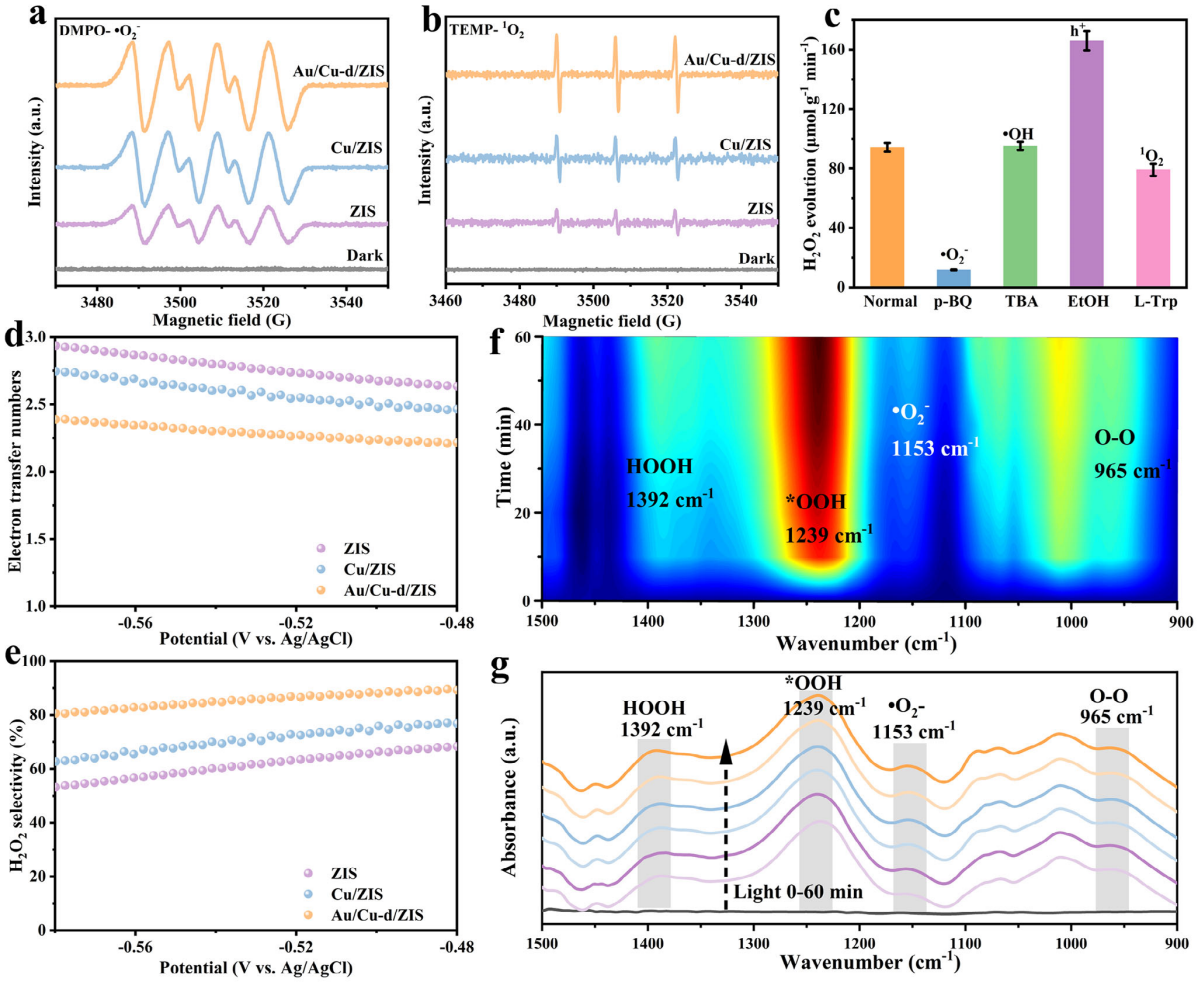
DMPO spin‐trapping ESR spectra for a) •O_2_^−^ and b) ^1^O_2_ of pristine ZIS, Cu/ZIS, and Au/Cu‐d/ZIS catalyst. c) Radical trapping experiments on Au/Cu‐d/ZIS catalyst. d) Electron transfer number and e) selectivity of H_2_O_2_ production. f,g) In situ DRIFTS spectra recorded over Au/Cu‐d/ZIS under irradiation.

Quenching experiments and electron spin resonance (ESR) techniques were employed to investigate the reaction mechanism underlying the enhanced photocatalytic H_2_O_2_ production.^[^ ^66^ ^]^ The as‐prepared Au/Cu‐d/ZIS catalyst exhibited significantly stronger •O_2_^−^ signals compared to pristine ZIS and Cu/ZIS (**Figure** **6**a). No •O_2_^−^ signals were detected under dark conditions, while the signals increased gradually with prolonged light irradiation, indicating that Au/Cu‐d/ZIS consistently produces intermediates that subsequently form H_2_O_2_ (Figure S38, Supporting Information). Non‐radical reactive oxygen species, such as singlet oxygen (^1^O_2_), were also examined. ESR results revealed that the Au/Cu‐d/ZIS catalyst generated stronger ^1^O_2_ signals than pristine ZIS and Cu/ZIS (Figure 6b). Radical trapping experiments aligned with these ESR results, as the addition of p‐benzoquinone (p‐BQ), a •O_2_^−^ scavenger, significantly reduced the H_2_O_2_ yield (Figure 6c). This finding highlights the critical role of •O_2_^−^ as an intermediate in H_2_O_2_ production, with the reaction primarily driven by photogenerated electron reduction.^[^ ^67^ ^]^ Conversely, no significant reduction in H_2_O_2_ yield was observed upon introducing tert‐butyl alcohol (TBA), a •OH scavenger. Interestingly, the addition of an isopropanol/ethanol mixture further increased H_2_O_2_ production (Figure 6c). This result is consistent with the previously discussed bandgap structure, as the valence band potential of Au/Cu‐d/ZIS does not support •OH production. Furthermore, capturing holes enhances the oxygen reduction reaction. The role of singlet oxygen (^1^O_2_) was confirmed by the decreased H_2_O_2_ yield upon introducing L‐tryptophan (L‐Trp), a ^1^O_2_ scavenger, which demonstrated that ^1^O_2_, formed via the oxidation of •O_2_^−^ by photogenerated holes, participates in the H_2_O_2_ generation process (Figures S39 and S40, Supporting Information).^[^ ^68^ ^]^ A rotating ring‐disk electrode (RRDE) was employed to elucidate the electron transfer number and H_2_O_2_ selectivity. The results showed that the Au/Cu‐d/ZIS catalyst exhibited an electron transfer number close to 2, as well as significantly higher H_2_O_2_ selectivity compared to pristine ZIS and Cu/ZIS (Figure 6d,e; Figure S41, Supporting Information).

In‐situ diffuse reflectance infrared Fourier transform spectroscopy (DRIFTS) was tested to explore the dynamic intermediates during photocatalytic H_2_O_2_ production (Figure 6f,g). Under illumination and presence of oxygen and water, an emerging signal trend of intermediates during H_2_O_2_ evolution process is observed when the reaction time increases. The peak detected at 1200 cm^−1^‐1250 cm^−1^ is ascribed to the formation of *OOH intermediates. Intermediate *OOH undergoes further hydrogenation (•O_2_^−^, 1167 cm^−1^) and reduction to produce H_2_O_2_.^[^ ^58^, ^59^ ^]^ These results confirm the existence of intermediates for H_2_O_2_ evolution through the ORR pathway, which are consistent with the ESR results and active radical trapping experiments.

**Figure 7:**
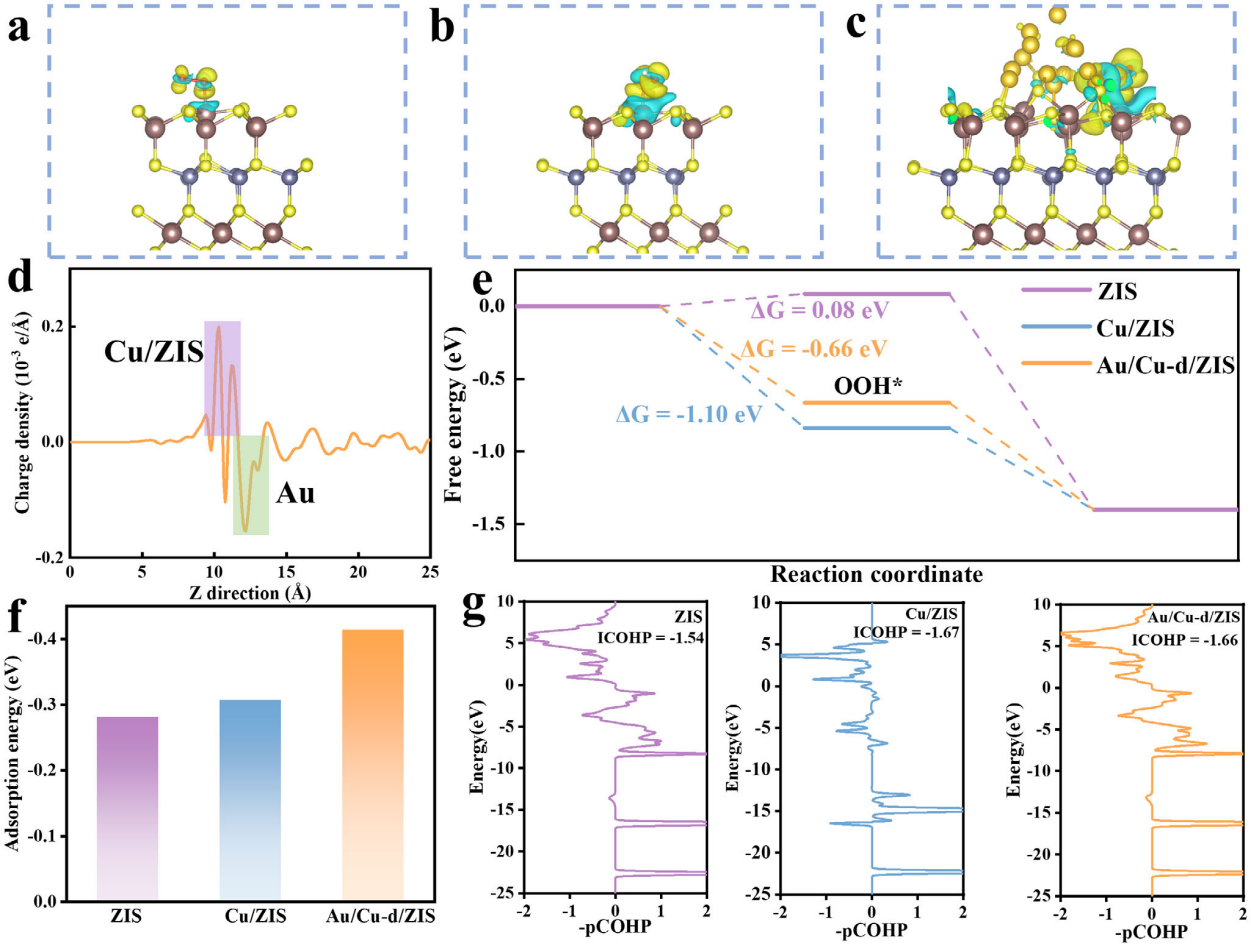
Differential charge density of *OOH of a) ZIS, b) Cu/ZIS, and c) Au/Cu‐d/ZIS catalyst. d) The planar‐averaged electron density difference over the Au/Cu‐d/ZIS catalyst. e) Free energy profiles of ORR toward H_2_O_2_ evolution in ZIS, Cu/ZIS, and Au/Cu‐d/ZIS. f) Adsorption energies of H_2_O on ZIS, Cu/ZIS, and Au/Cu‐d/ZIS. g) The COHP of ZIS, Cu/ZIS and Au/Cu‐d/ZIS.

To gain a deeper understanding of the promotion of Au and Cu to produce H_2_O_2_ by ZIS, we further conducted a detailed analysis through density functional theory (DFT). Differential charges between the key intermediate *OOH and different catalysts were analyzed. The results showed that the strongest interactions existed between Au/Cu‐d/ZIS and *OOH, suggesting that Au and Cu jointly promoted the electron transfer between ZIS and *OOH, greatly facilitating the H_2_O_2_ production (**Figure** **7**a–c). To elucidate the charge transfer kinetics at the interface of Au/Cu‐d/ZIS, the plane‐averaged charge density difference between Cu/ZIS and Au was analyzed (Figure 7d). The results showed that Cu/ZIS donates electrons to Au, thus leading to a positive charge of Cu/ZIS at the contact surface and a negative charge of Au at the contact surface, forming an in‐built electric field (IEF) between Cu/ZIS and Au. The hot electrons generated by Au will be transferred to Cu/ZIS under the synergistic effect of photo‐excitation and IEF, which agrees with the in‐situ XPS results and theoretical work functions (Figure S42, Supporting Information). The Gibbs free energies of ORR on ZIS, Cu/ZIS, and Au/Cu‐d/ZIS were further calculated to understand the catalytic properties of different catalysts, and it was found that Au/Cu‐d/ZIS exhibited the most favorable Gibbs free energy (Figure 7e). Au/Cu‐d/ZIS also exhibited the optimal H_2_O adsorption capacity, superior to that of ZIS and Cu/ZIS (Figure 7f). To quantitatively evaluate the interactions arising from electron transfer, crystal orbital Hamiltonian population (COHP) was calculated, and showed that the introduction of Au and Cu resulted in a significant correlation between the *OOH and the catalytic performance. The results showed that Au/Cu‐d/ZIS (−1.66 eV) and Cu/ZIS (−1.67 eV) is stronger than the interaction in the pristine ZIS (−1.54 eV) (Figure 7g). The above results indicated that Au and Cu co‐modification in ZIS can effectively promote the interaction between the catalyst and O_2_ to produce H_2_O_2_.

## 3 Conclusion

In summary, the integration of Au nanoparticles with Cu atom‐doped Zn_3_In_2_S_6_ (Au/Cu‐d/ZIS) successfully achieves synchronous carrier capture and transfer, leading to enhanced photocatalytic performance. The optimized Au/Cu‐d/ZIS catalyst demonstrated an exceptional H_2_O_2_ production rate of 94.2 µmol g^−1^ min^−1^ in pure water without the use of sacrificial agents, outperforming most previously reported photocatalysts. In‐situ XPS and femtosecond transient absorption spectroscopy revealed that the enhanced photocatalytic activity stems from efficient bulk carrier capture and the accumulation of abundant photogenerated electrons. Additionally, theoretical calculations and in situ Fourier transform infrared spectroscopy confirmed the pivotal roles of Au and Cu in facilitating the production of H_2_O_2_ through the oxygen reduction reaction in ZIS. This work provides valuable insights into the design of advanced catalyst architectures that enable efficient carrier capture and transfer at the atomic scale, paving the way for future developments in artificial photosynthesis systems.

## Conflict of Interest

The authors declare no conflict of interest.

## Supporting information



Supporting Information

## Data Availability

Research data are not shared.
