# Supplementary material for: Decoupling of Carrier Pathways in Au/Cu‐Zn3In2S6 Through Bulk Hole Trapping and Surface Hot Electron Accumulation Enhances Photocatalytic Hydrogen Peroxide Production
Source: Adv Mater. 2025 Aug 18;37(44):e11422. doi: 10.1002/adma.202511422 (PMC12592919; doi:10.1002/adma.202511422)
Supplement: Supplementary file 1 — Supporting Information [file ADMA-37-e11422-s001.docx]

**Decoupling of Carrier Pathways in Au/Cu-Zn₃In₂S₆ through** **Bulk Hole Trapping and Surface Hot Electron Accumulation Enhances Photocatalytic Hydrogen Peroxide Production**

Xiaowen Ruan,^#[1]^ Chunsheng Ding,^#[2]^ Jing Leng,^[3]^ Dongxu Jiao,^[2]^ XiangXiang Zhang,^[4]^ Minghua Xu,^[2]^ Depeng Meng,^[2]^ Xiaoqiang Cui^*[2]^ Zhaoke Zheng^*[4]^ Yongfa Zhu^*[5]^, and Sai Kishore Ravi^*[1]^

^[1]^ School of Energy and Environment, City University of Hong Kong, Tat Chee Avenue, Kowloon, Hong Kong SAR, 999077, China

^[2]^ School of Materials Science and Engineering, Key Laboratory of Automobile Materials of MOE, Jilin University, Changchun 130012, China

^[3]^ State Key Laboratory of Chemical Reaction Dynamics, Dalian Institute of Chemical Physics, Chinese Academy of Sciences, Dalian 116023, China

^[4]^ State Key Laboratory of Crystal Materials, Shandong University, Jinan 250100, China

^[5]^ Department of Chemistry, Tsinghua University, Beijing 100084, China

#These authors contributed equally

*Corresponding Author: Xiaoqiang Cui; Sai Kishore Ravi; Zhaoke Zheng; Yongfa Zhu

E-mail: xqcui@jlu.edu.cn; skravi@cityu.edu.hk; zkzheng@sdu.edu.cn; zhuyf@tsinghua.edu.cn

**Table of Contents**

[**1.** **Chemicals and materials** 3](#_Toc196931656)

[**2.** **Synthesis of samples** 3](#_Toc196931657)

[**2.1 The synthesis of** **copper doped Zn_3_In_2_S_6_ (Cu/ZIS)** 3](#_Toc196931658)

[**2.2 The synthesis of Au/Cu-d/ZIS** 3](#_Toc196931659)

[**3.** **Characterization** 4](#_Toc196931660)

[**4.** **Photocatalytic H_2_O_2_ production** 5](#_Toc196931661)

[**5.** **Photoelectrochemical measurements** 6](#_Toc196931662)

[**6.** **The rotating ring disk electrode (RRDE) measurement** 6](#_Toc196931663)

[**7.** **In situ Fourier transform infrared (FTIR) Spectroscopy** 7](#_Toc196931664)

[**8.** **Computational methods** 7](#_Toc196931665)

[**9.** **Figure S1-S42** 9](#_Toc196931666)

[**10.** **Table. S1** 30](#_Toc196931667)

[**11. References** 31](#_Toc196931668)

1. **Chemicals and materials**

Zinc sulphate heptahydrate (ZnSO_4_.7H_2_O, 99.995%), indium trichloride tetrahydrate (InCl_3_.4H_2_O, 99.99%), Copper chloride (CuCl_2_, 99.9%), potassium hydrogen phthalate (99.8%), para-benzoquinone (p-BQ, 99%), urea, and absolute ethanol were purchased from Macklin Biochemical Technology Co., Ltd. Thioacetamide (TTA, 98%), Gold chloride trihydrate (HAuCl_4_.3H_2_O, 99.9%), phosphate buffer (pH=7.0), tert-butyl alcohol (TBA, 99.5%), Manganese acetate dihydrate (MnAC_3_, 97%), potassium iodide (KI, 99%), L-Tryptophan (L-trp, 99%), 4-Carboxyphenylboronic acid (97%) were purchased from Aladdin Biochemical Technology Co., Ltd. All the purchased drugs can be used directly without any purification.

1. **Synthesis of samples**

**2.1 The synthesis of** **copper doped Zn_3_In_2_S_6_ (Cu/ZIS)**

3 mmol ZnSO_4_.7H_2_O and 2 mmol InCl_3_.4H_2_O were dissolved in 35 ml of deionized water, after stirring for 30 min, add 8.46 mg CuCl_2_ to the above solution and then continue stirring for 15 min. Finally, added 12 mmol TAA, after stirring for 30 min, the mixture was transferred to a 50 ml Teflon-lined stainless-steel reactor and the reaction time and temperature were set at 12 h and 160 °C, respectively. After cooling to room temperature, the precipitate was collected by centrifugation and washed three times with distilled water and ethanol, then dried in a vacuum oven at 60 °C. The Cu doping concentration obtained through ICP test was 0.52 wt%. The preparation of Zn_3_In_2_S_6_ (ZIS) is similar to that of Cu-ZIS, except that CuCl_2_ is not added.

**2.2 The synthesis of Au/Cu-d/ZIS**

Au/Cu-d/ZIS prepared by photo-deposition, 200 mg of Cu/ZIS was dispersed in an aqueous ethanol solution (20 % v/v ethanol), followed by the addition of 4 mg of HAuCl_4_.3H_2_O, which was illuminated under argon for 30 min. Then the precipitate was collected by centrifugation and washed three times with distilled water and ethanol, then dried in a vacuum oven at 60 °C. After that, the sample was heat-treated at 200 °C for 2 h under air conditions, and the heating rate was set at 5 °C/min and the sample obtained is named Au/Cu-d/ZIS. The synthesis of Au/ZIS is similar to that of Au/Cu-d/ZIS, except that Cu/ZIS is replaced by ZIS. By controlling the mass of HAuCl_4_.3H_2_O (10 mg and 20 mg), Au/ZIS-10, Au/Cu-d/ZIS-10, Au/ZIS-20 and Au/Cu-d/ZIS-20 were additionally synthesized. For Pt/ZIS and Ru/ZIS, the synthesis method is similar to that of Au/ZIS, except that HAuCl_4_.3H_2_O is replaced with the same mass of H_2_PtCl_6_.6H_2_O and RuCl_3_.xH_2_O. In addition, for Pt/Cu-d/ZIS and Ru/Cu-d/ZIS, the synthesis method is similar to that of Au/Cu-d/ZIS, except that HAuCl_4_.3H_2_O is replaced with the same mass of H_2_PtCl_6_.6H_2_O and RuCl_3_.xH_2_O.

1. **Characterization**

The crystal structure is revealed by the powder X-ray diffraction (XRD) on a Bragg-Brentano diffractometer (D8-tools, Germany) equipped with a Cu Kα source, and the scanning region was from 10° to 80°. A JEM-2000EX transmission electron microscope (JEOL Co., Japan) was used to acquire TEM images of the as-prepared samples with an acceleration voltage of 200 kV. The transmission electron microscopy (TEM) and high-resolution TEM (HRTEM) images were acquired by a JEOLJEM-2100F (UHR) Field Emission Transmission Electron Microscope. X-ray photoelectron spectroscopy (XPS) was performed on a Thermo ESCALAB 250Xi instrument with Al KαX-ray radiation. In-situ XPS was conducted under the same condition, except that ultraviolet light irradiation. The UV-visible absorption spectra of the samples were obtained by UV-vis spectrophotometer (Shimadzu, UV-2550, and Japan). With the excitation wavelength of 365 nm, SHIMADZU RF-6000 was used to analyze the photoluminescence (PL) spectra and Time-resolved photoluminescence (TRPL) spectrum of the photocatalysts. Electron spin resonance (ESR) analysis was performed using electron spin resonance spectrometer (Jeol/JES-FA200). The femtosecond transient absorption setup is based on a regenerative amplified Ti:sapphire laser system from Coherent (800 nm, 35 fs, 6 mJ pulse^-1^, and 1 kHz repetition rate), nonlinear frequency mixing techniques and the Femto-TA100 spectrometer (Time-Tech Spectra LLC). Fourier-transform infrared (FTIR) spectra were conducted on a Bruker INVENIO R infrared spectrophotometer. Agilent 1100-Thermos TSQ Quantum Ultra for isotope liquid chromatography mass spectrometry testing.

1. **Photocatalytic H_2_O_2_ production**

Photocatalytic H_2_O_2_ production was carried out under pure water and O_2_ conditions without any sacrificial agents. Specifically, 5 mg of photocatalyst was dispersed in 40 mL of ultrapure water, immediately followed by switching on the xenon lamp for the photocatalytic reaction. The generation of H_2_O_2_ was investigated by iodometry, specifically, 1 mL of the solution removed from the reactor was added to 1 mL of 0.4 M aqueous potassium iodide (KI) and 1 mL of 0.1 M aqueous potassium hydrogen phthalate, the solution was then analyzed using a UV-visible spectrophotometer at 350 nm.

Decomposition experiments were performed under argon on commercial H_2_O_2_ for degradation. Specifically, 5 mg of catalyst was placed in 40 ml of commercial H₂O₂ solution with a concentration of 1 mM, followed by the introduction of argon gas into the solution for 20 minutes. Finally, the light was turned on for irradiation for 20 minutes. The changes in the concentration of H_2_O_2_ solution before and after irradiation were compared. The concentration of H_2_O_2_ before the reaction was C_0_, while the concentration after the reaction was C. The decomposition rate constant (K_d_, min^-1^) of H_2_O_2_ follows first-order kinetics and can be calculated using the following formula: K_d_ = -ln(C/C_0_)/t, as for the H_2_O_2_ theoretical generation rate constant (K_f_, μM min^-1^), using the following formula:

$$K_{f}=\frac{K_{d}C_{{H_{2}O}_{2}}}{(1-e^{{-k}_{d}t})}$$

Among them, $C_{{H_{2}O}_{2}}$is the concentration of H_2_O_2_ produced by the photocatalyst. Decomposition experiments conducted in darkness are like those conducted under light, except that no light is used.

Photocatalytic H_2_O_2_ production experiments under sunlight were carried out with 20 mg of catalyst and a solution volume of 200 mL. In addition, antimicrobial experiments were applied to observe solar photocatalytic production of H_2_O_2_. Rhodospirillum, Alcaligenes and Escherichia coli as model organisms and cultured on nutrient agar plates using sterile petri dishes. Subsequently, the bacterial concentration was controlled at 10^3^ CFU mL^-1^ with PBS, and then 2 mL of the reaction solution after photocatalysis was removed and added to 1 mL of bacteria suspension and mixed thoroughly. Finally, the solution (3 mL) was added to the nutrient agar and incubated at 37 °C for 24 hours. The in-situ generation of H_2_O_2_ by the catalyst was further evaluated for the degradation of antibiotic pollutants at a concentration of 10 mg/L antibiotic and 1 g/L catalyst. In addition, the detoxification of antibiotics by the photocatalytic system was also characterized, Mung bean sprouts were used in this experiment to test the toxicity of TC before and after degradation due to their short growth period. Mung bean seeds were immersed in DI, TC solution and TC solution after catalytic degradation for 8 h at room temperature and subsequently grown at room temperature for 96 h. Finally, the length of the mung bean sprout rootstock was recorded.

1. **Photoelectrochemical measurements**

Photoelectrochemical experiments were performed on CHI650D electrochemical workstation with a conventional three electrode cell using Pt as the counter electrode, an Ag/AgCl electrode as the reference electrode. The preparation of the working electrode is as follows: 5 mg of photocatalyst was dispersed in mixed solution (2 mL) of water, isopropyl alcohol and Nafion solution (1:1:0.025) to obtain a slurry by ultrasonication 30 min. Appling the dispersion dropwise on the FTO and control the area to 1 cm^2^, and 0.5 M Na_2_SO_4_ as electrolyte. The light was provided by a 300 W Xe lamp.

1. **The rotating ring disk electrode (RRDE) measurement**

RRDE test was used to evaluate the number of transferred electrons (n) and H_2_O_2_ selectivity in the ORR reaction. The RRDE tests were conducted in an O_2_-saturated phosphate buffer (pH=7.0) solution with a rotating speed of 1600 rpm. The number of transferred electrons (n) is calculated according to the following formula:

$$n=4\times\frac{I_{d}}{I_{d}+I_{r}/N}$$

The selectivity of H_2_O_2_ is calculated by the following formula:

$$H_{2}O_{2}\%=200\times\frac{I_{r}/N}{I_{d}+I_{r}/N}\times100\%$$

where I_r_ is the ring current, I_d_ is the disk current, and N is the collection efficiency (N = 0.256).

1. **In situ Fourier transform infrared (FTIR) Spectroscopy**

The samples were pressed and placed into a reaction chamber and high purity helium gas was introduced for 30 min to remove small molecules such as H_2_O, O_2_ and CO_2_ adsorbed on the surface of the specimen. The samples were immediately scanned using IR spectroscopy and the results were used as a background baseline. Then 40 mL∙min^-1^ of O_2_ (containing water vapor 5%) was injected into the reaction chamber. Under dark conditions, the samples were subjected to an adsorption reaction for 60 min, and the adsorption state on the surface was examined every 10 min. Subsequently, the inlet and outlet of the reaction chamber were closed, and the photocatalytic reaction was carried out under full-spectrum irradiation for 60 min, and the infrared spectra were recorded at given time intervals, with the infrared spectral scanning area in the range of 4000-800 cm^-1^.

1. **Computational methods**

All spin−polarized density functional theory (DFT) calculations were performed with the plane−wave basis set as implemented in the Vienna Ab Initio Simulation Package (VASP),^1, 2^ and the electrons and ions interactions were described by the projector augmented wave (PAW) potential.^3, 4^ The exchange–correlation interactions were determined by the Perdew–Burke–Ernzerhof (PBE) functional within the generalized gradient approximation (GGA).^5^ The plane wave energy cutoff of 500 eV, and the convergence criterion for the residual force and energy was set to 0.05 eV Å^−1^ and 10^−5^ eV, respectively. The empirical correction in Grimme's method (DFT+D3) was used to describe the van der Waals (vdW) interactions.^6^ The Brillouin region was sampled by the Monkhorst-Pack method with a 3× 3 × 1 k-point mesh. The change in the Gibbs free energy change (ΔG) for each possible step during the electrochemical synthesis of urea was obtained using the computational hydrogen electrode (CHE) model.^7, 8^ According to this model, the changes in Gibbs free energy (∆*G*) for all electrochemical steps was defined as: Δ*G* = Δ*E* + Δ*E_ZPE_* − *T*Δ*S*, where the reaction energy (Δ*E*) can be directly obtained by analyzing the DFT total energies. The zero-point energy difference (Δ*E*_ZPE_) between the products and the reactants can be computed from the vibrational frequencies. Δ*S* is the change in entropy between the products and the reactants at room temperature (T = 298.15 K).

1. **Figure S1-S42**


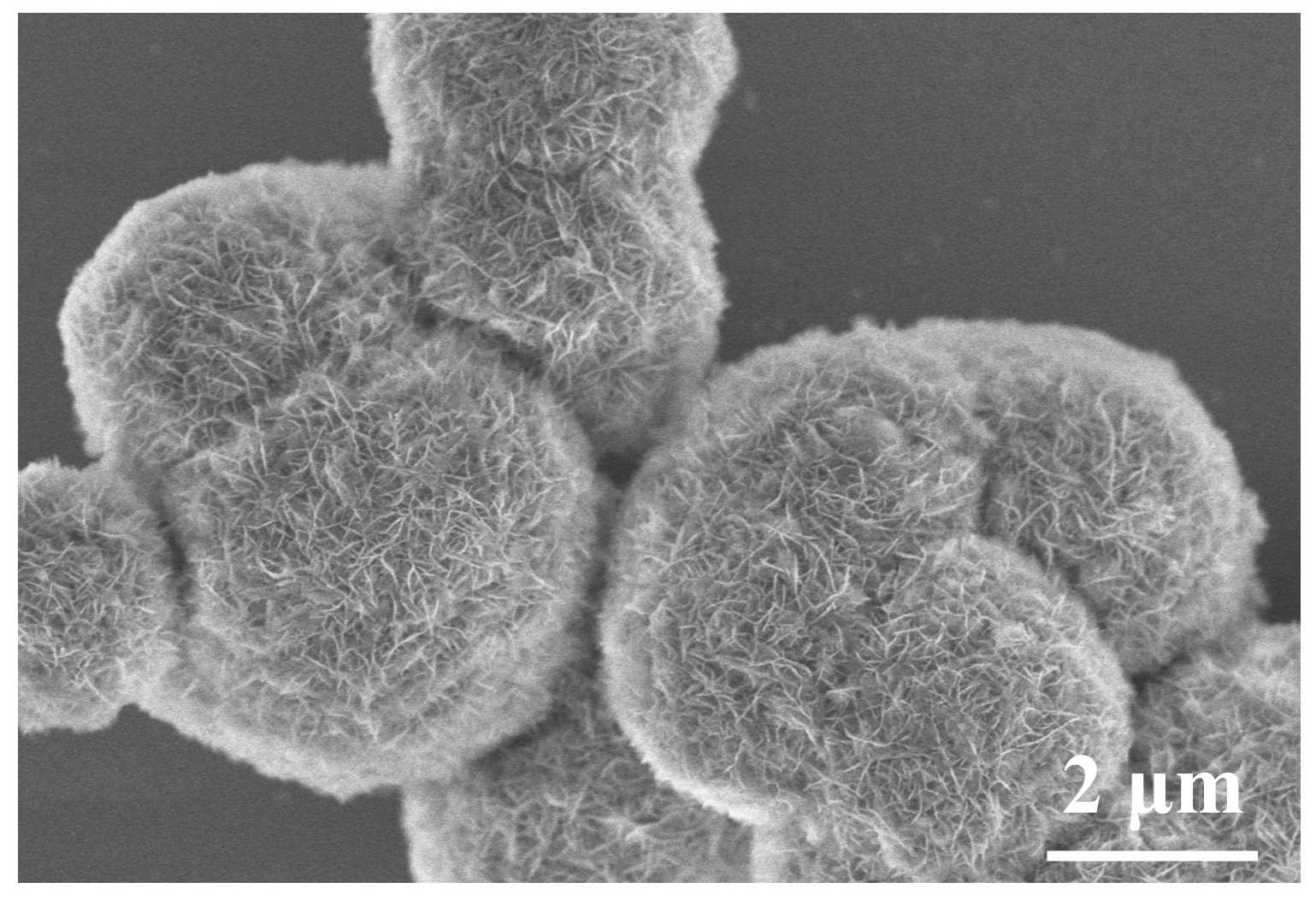


**Figure S1** SEM image of ZIS.


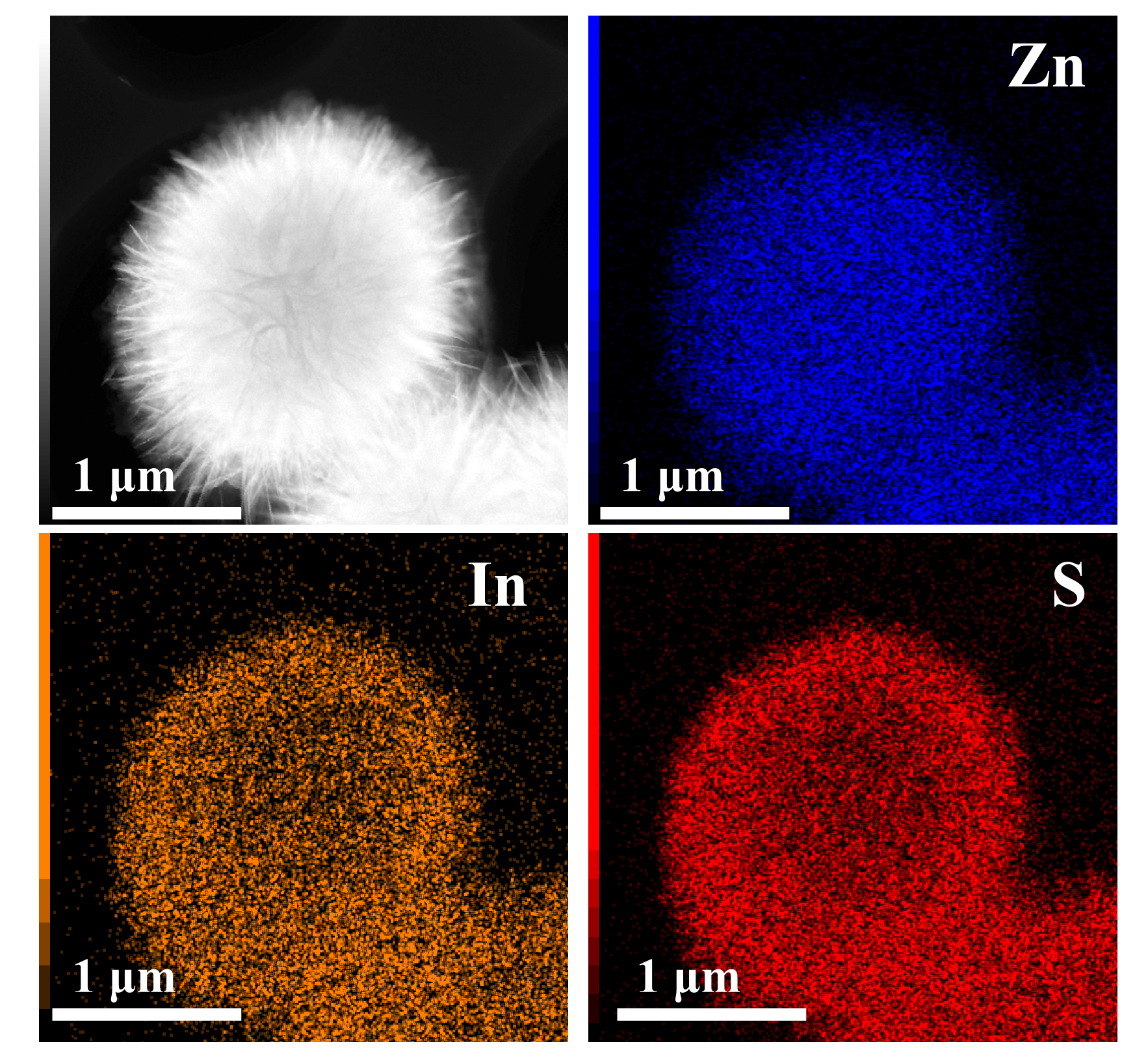


**Figure S2** Element distribution mapping images of ZIS.


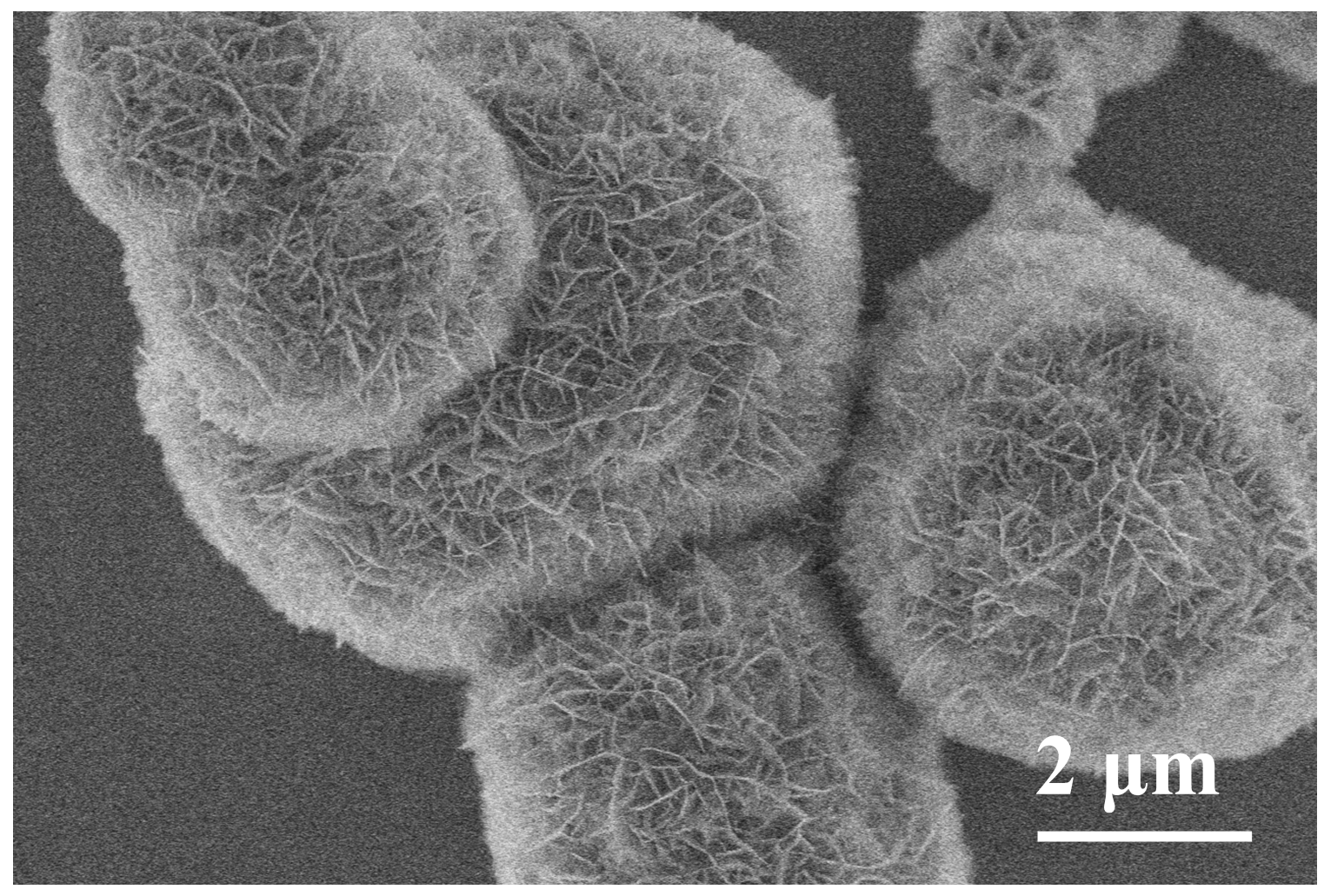


**Figure S3** SEM image of Cu/ZIS.


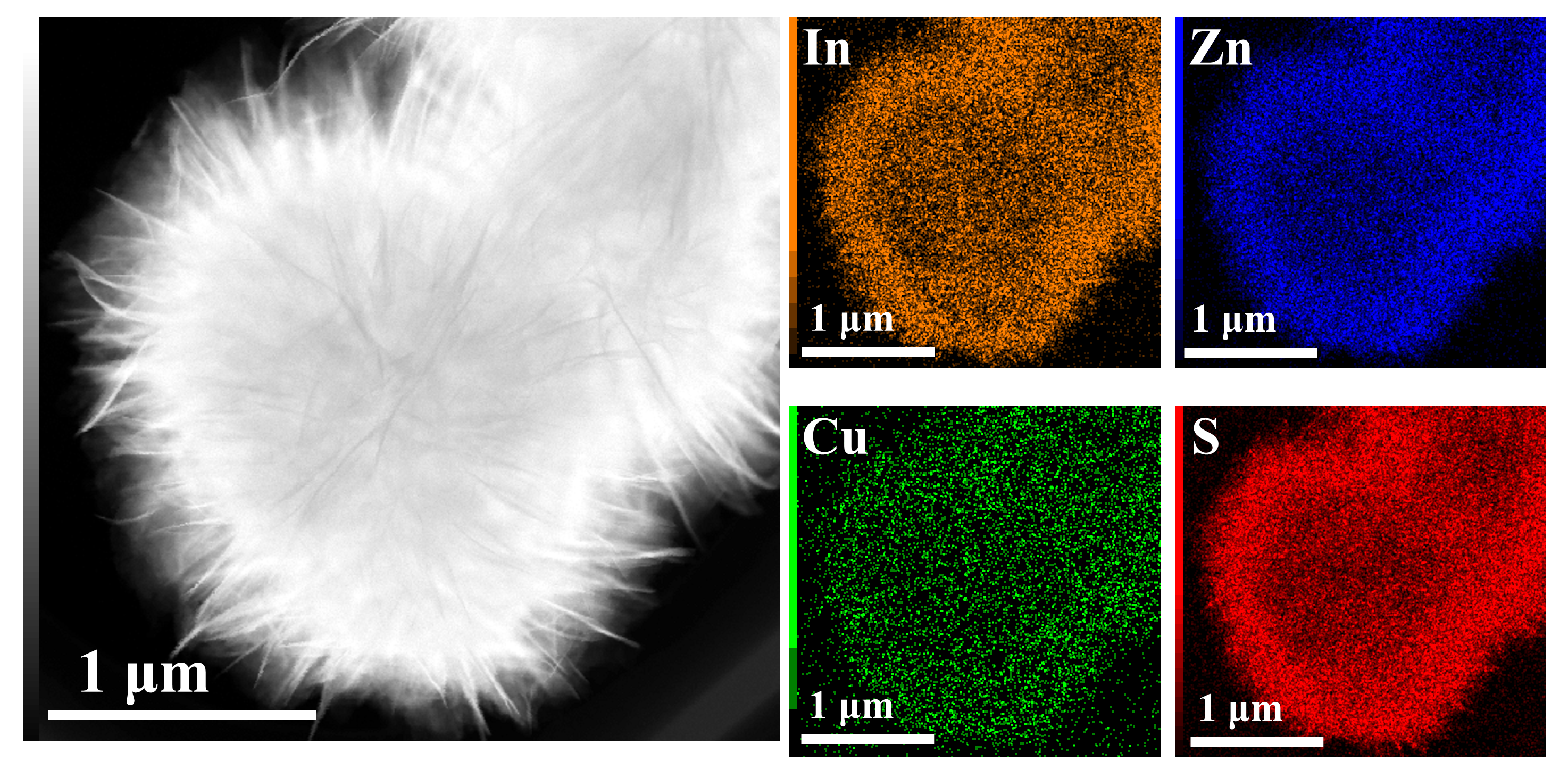


**Figure S4** Element distribution mapping images of Cu/ZIS.


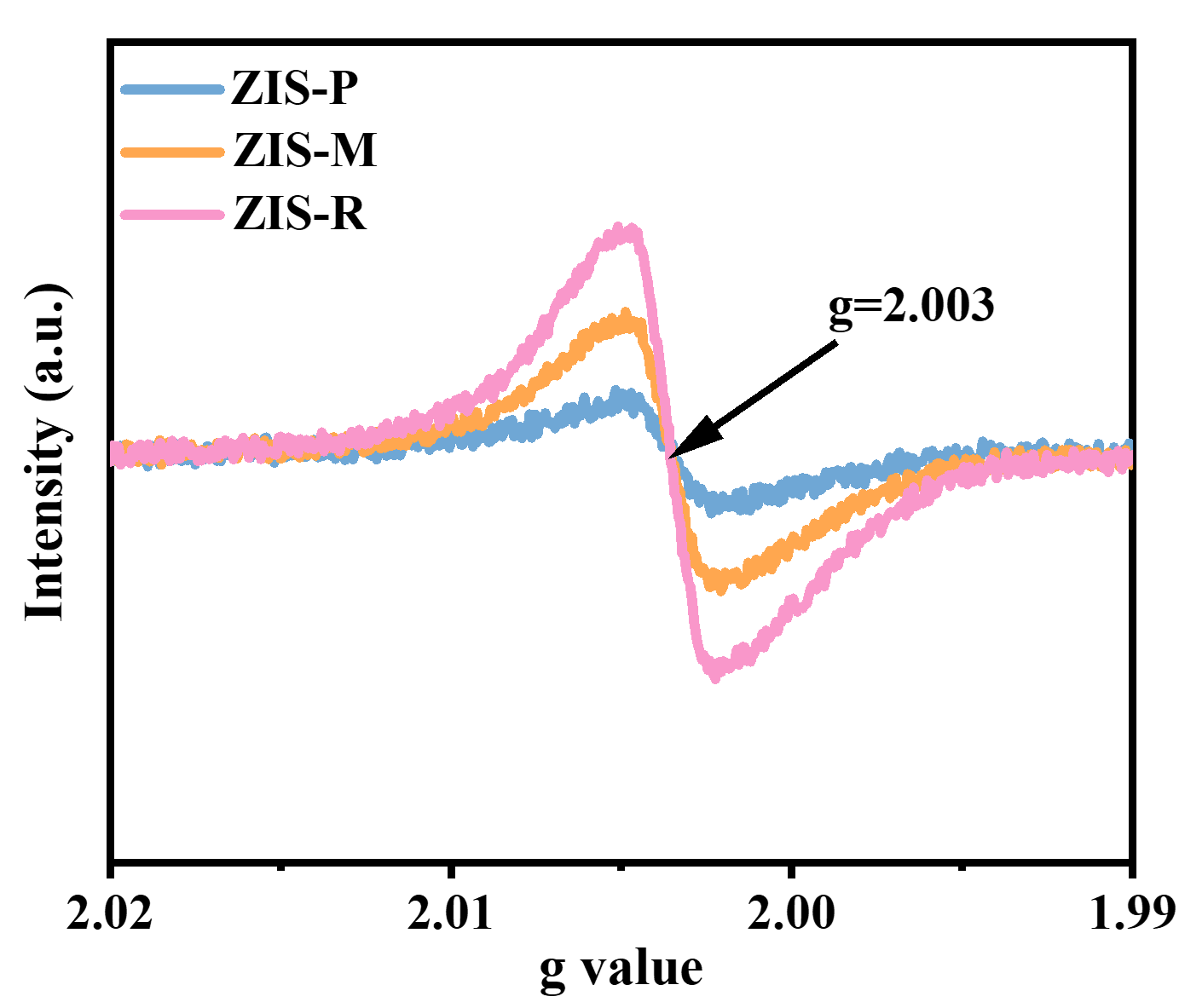


**Figure S5** EPR of ZIS with different concentrations of sulfur vacancies (Note: P, M and R represents poor, moderate and rich vacancy, respectively).


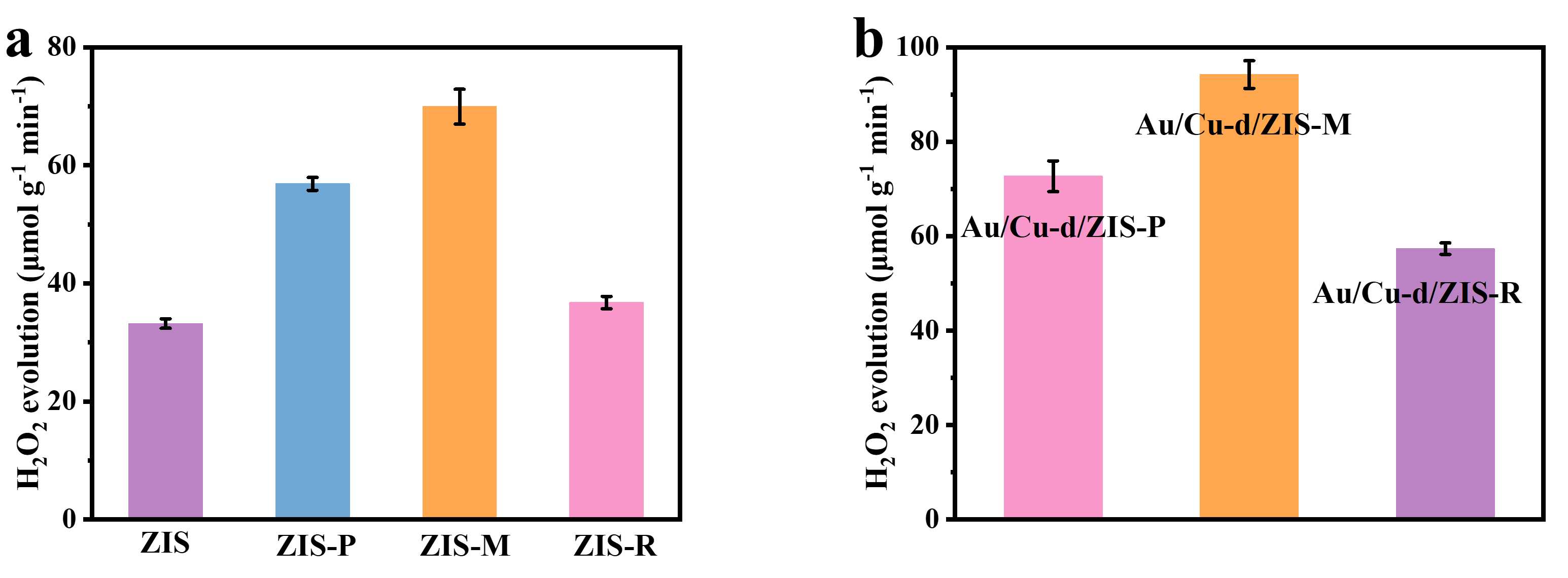


**Figure S6** Photocatalytic H_2_O_2_ evolution of (a) ZIS and (b) Au/Cu-d/ZIS with different concentrations of sulfur vacancies.


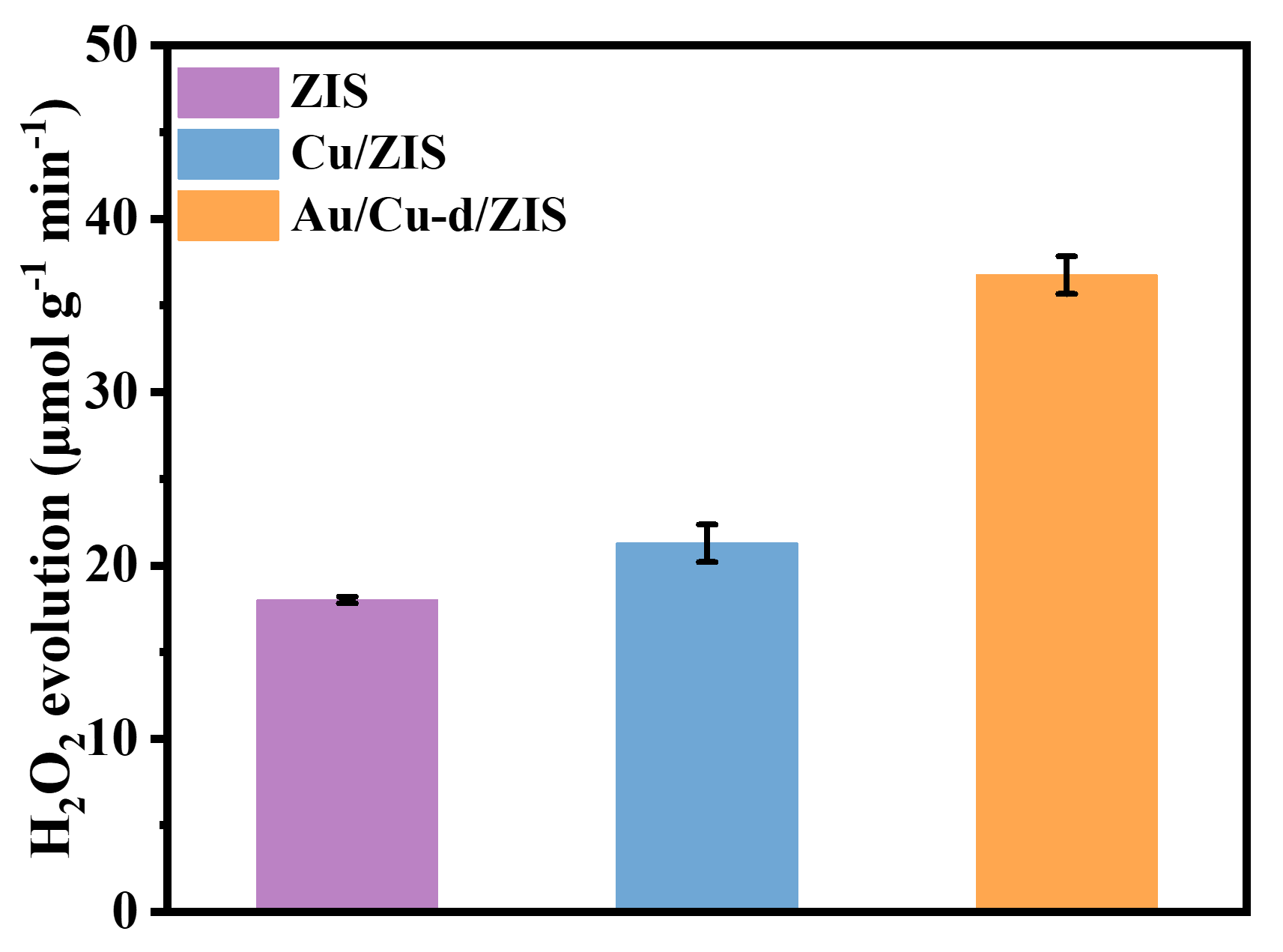


**Figure S7** Photocatalytic H_2_O_2_ evolution of samples under visible light irradiation.


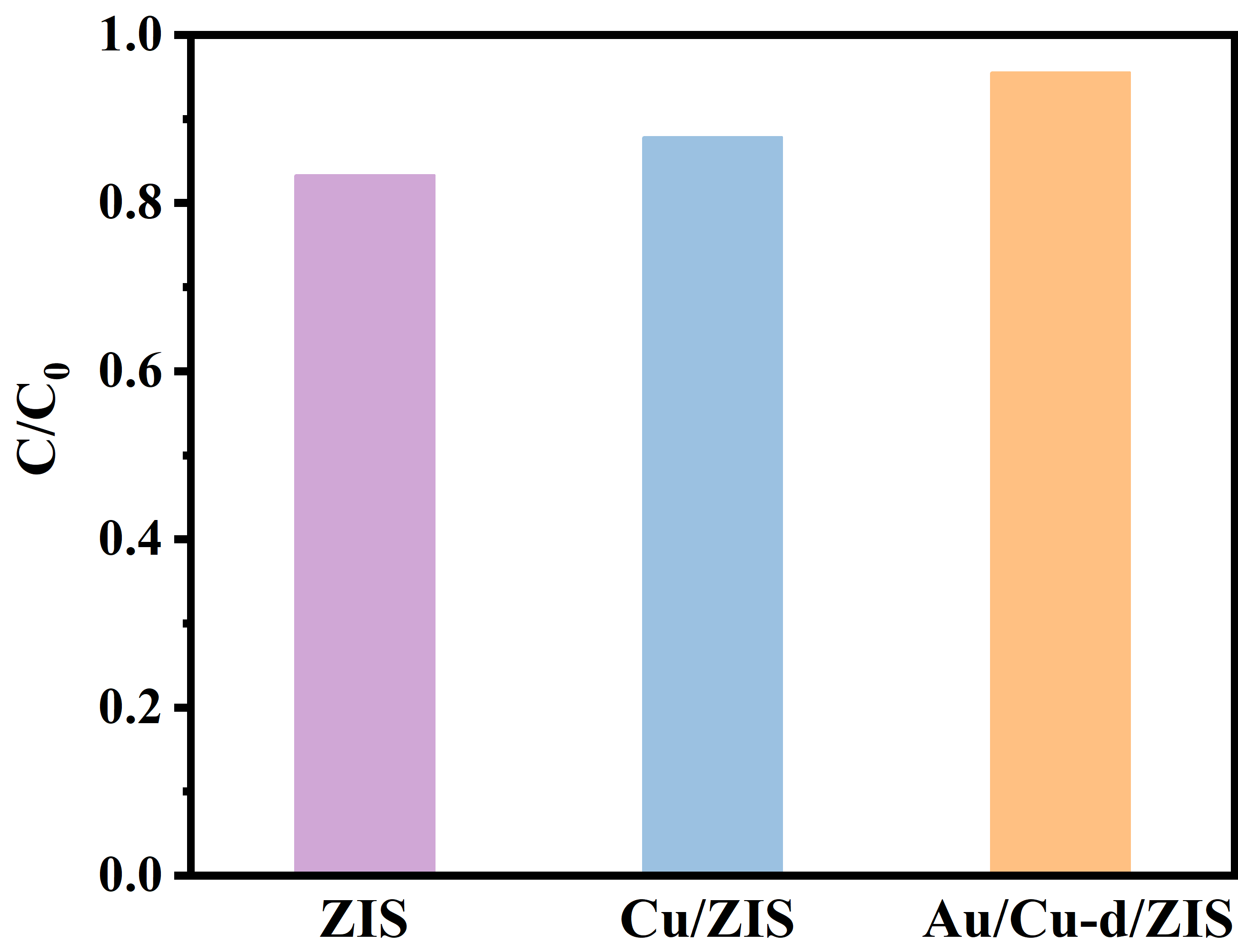


**Figure S8** Photocatalytic decomposition of H_2_O_2_ (1 mM) by different samples in the Ar-saturated pure water within 20 min.


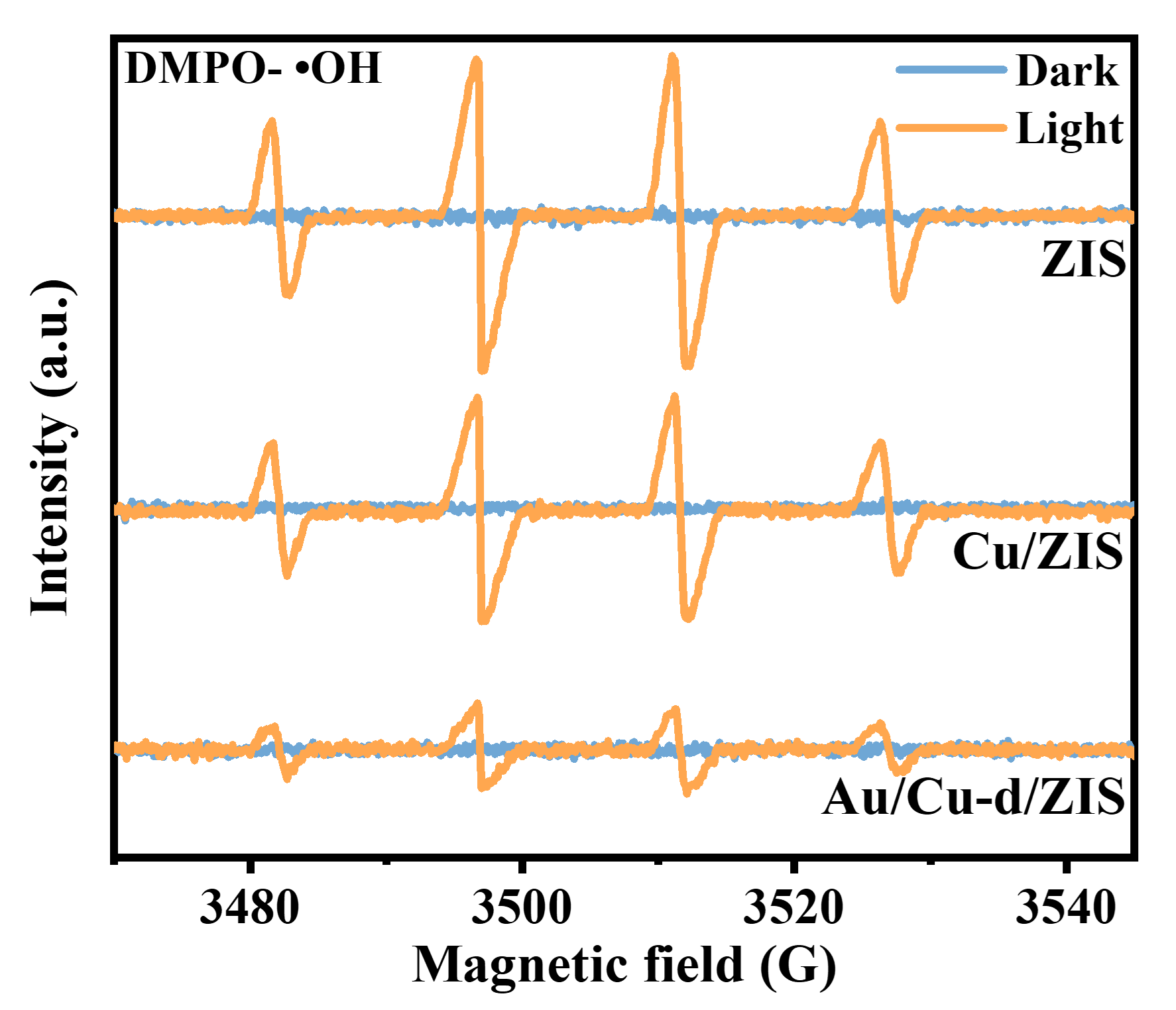


**Figure S9** DMPO spin-trapping ESR spectra for (a) •OH of pristine ZIS, Cu/ZIS and Au/Cu-d/ZIS catalyst in H_2_O_2_ solution under argon atmosphere.


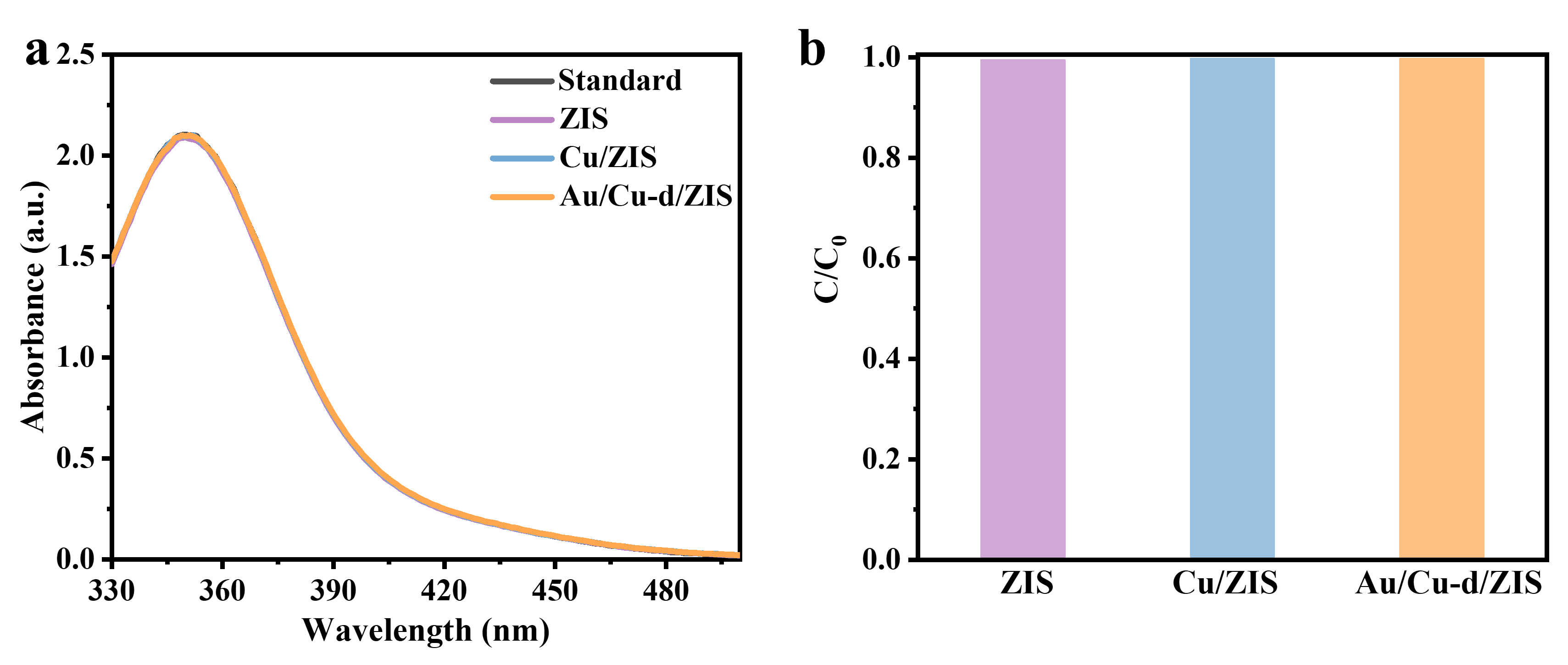


**Figure S10** (a) H_2_O_2_ degradation of pristine ZIS, Cu/ZIS, and Au/Cu-d/ZIS in the ultraviolet-visible absorption spectrum (three times diluted concentration) under dark conditions. (b) Decomposition of H_2_O_2_ (1 mM) by different samples in the Ar-saturated pure water within 20 min under dark conditions.


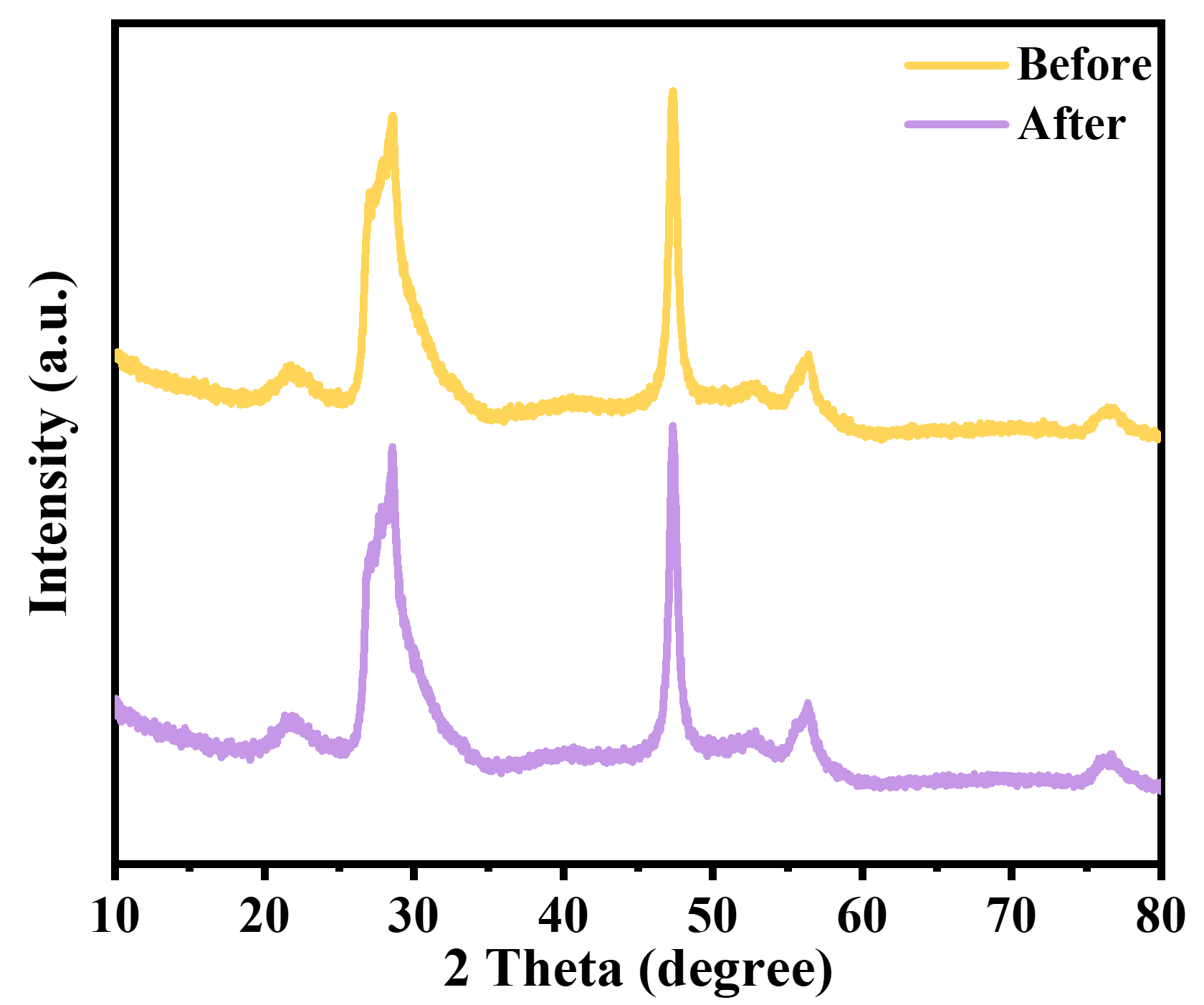


**Figure S11** XRD patterns of Au/Cu-d/ZIS before and after photocatalytic H_2_O_2_ evolution test.


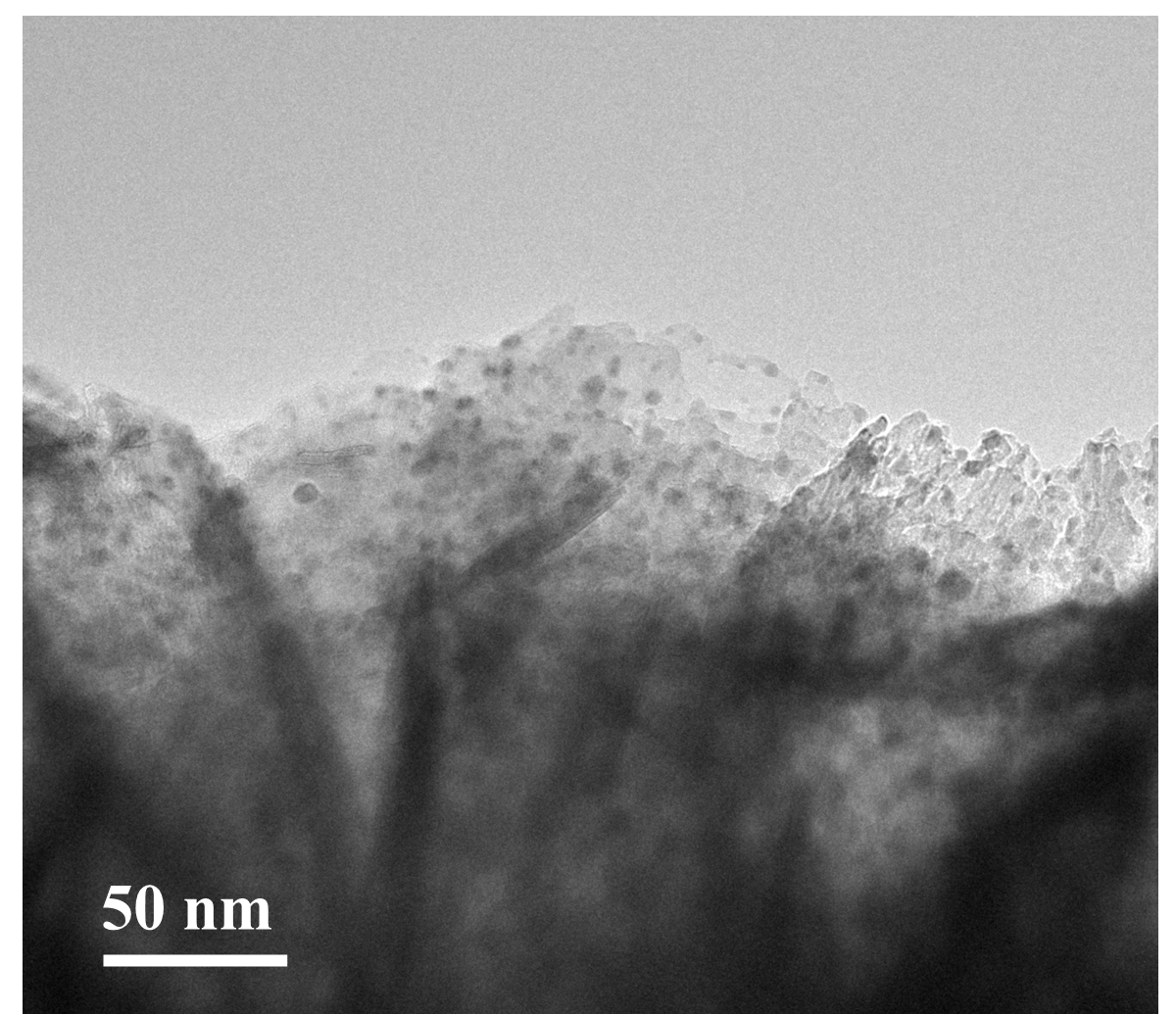


**Figure S12** TEM image of Au/Cu-d/ZIS after photocatalytic H_2_O_2_ evolution test.


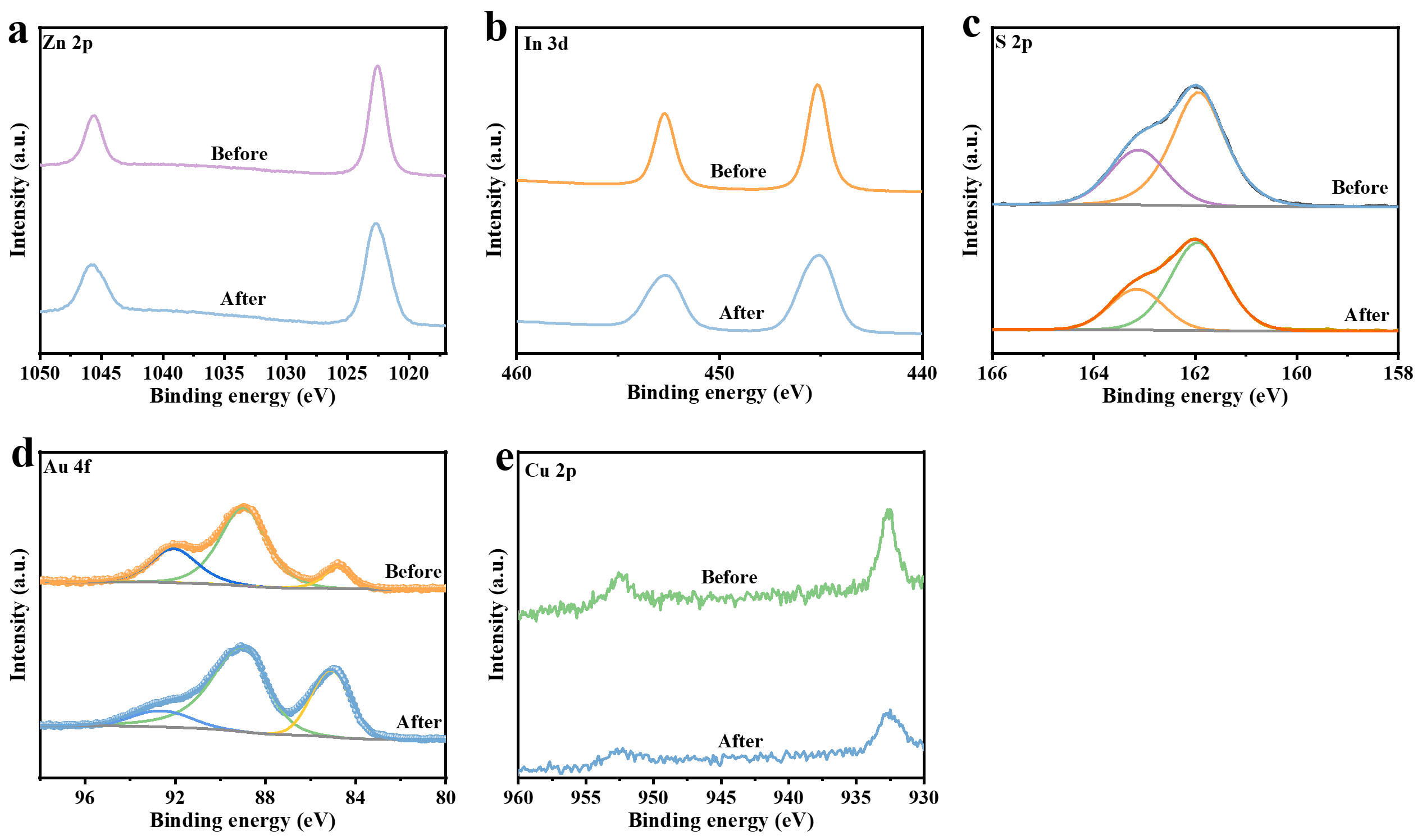


**Figure S13** (a) Zn 2p, (b) In 3d, (c) S 2p, (d) Au 4f and (e) Cu 2p XPS spectra of Au/Cu-d/ZIS before and after photocatalytic H_2_O_2_ evolution test.


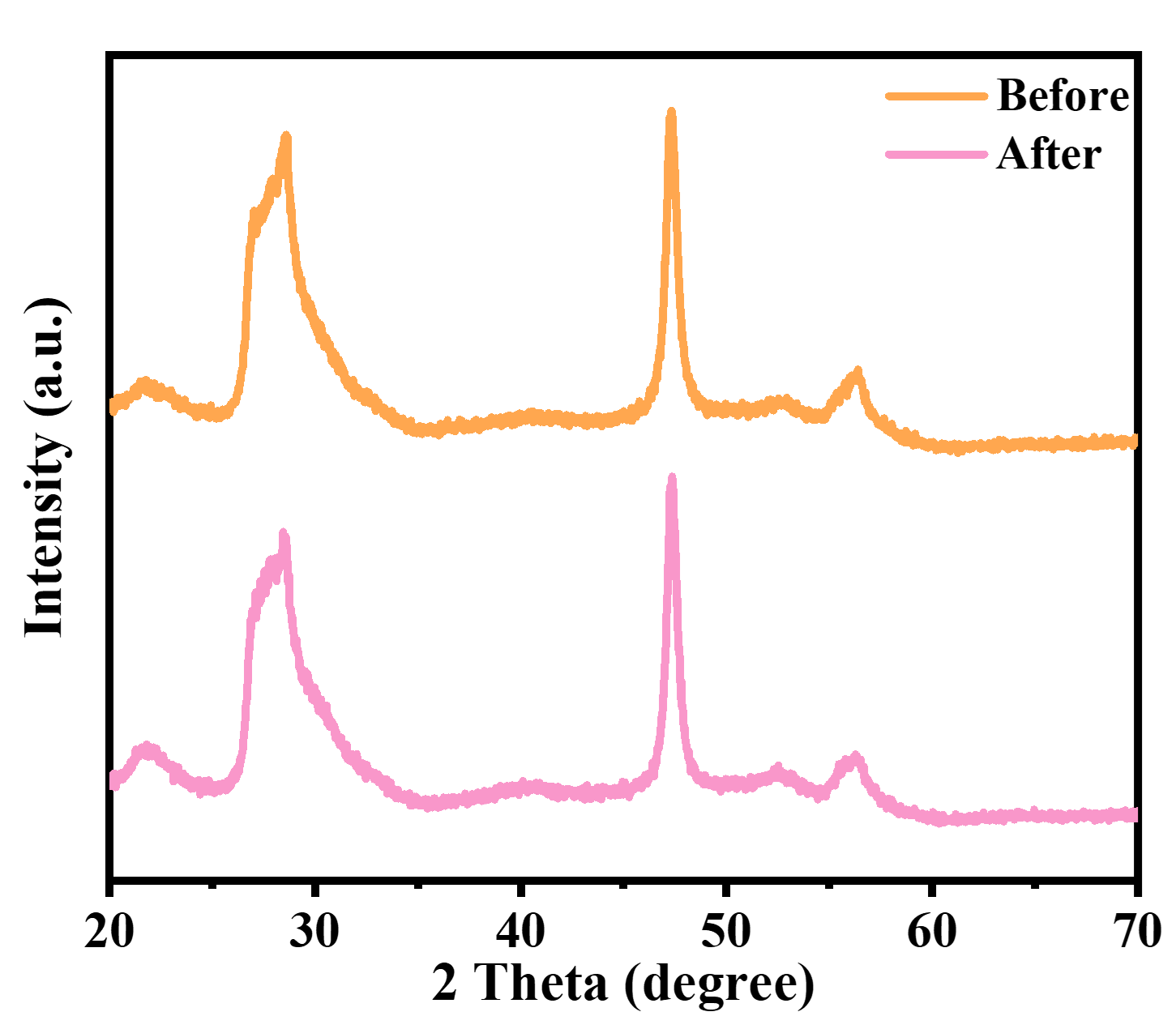


**Figure S14** XRD patterns of Au/Cu-d/ZIS before and after long-term photocatalytic H_2_O_2_ evolution test.


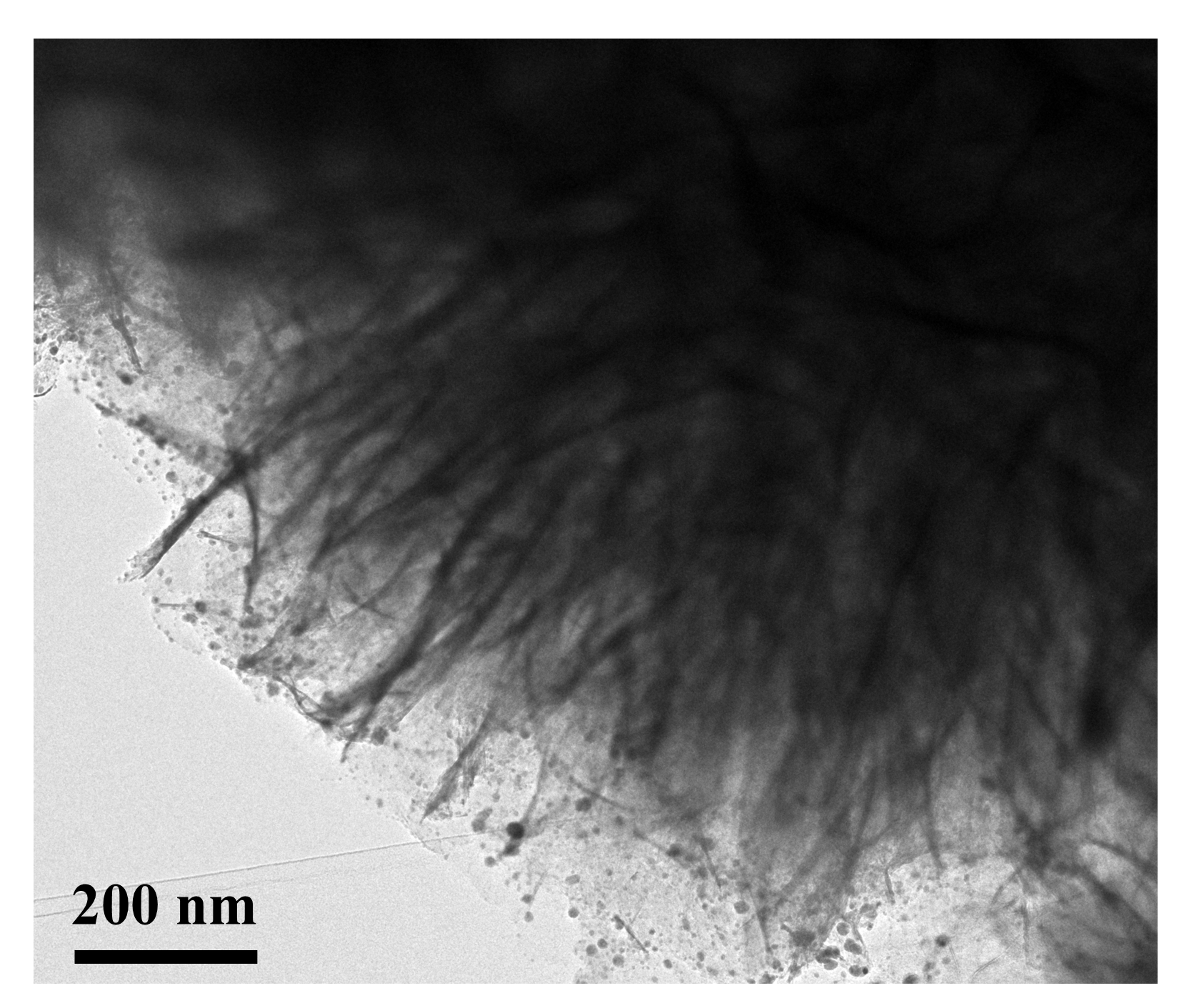


**Figure S15** TEM image of Au/Cu-d/ZIS after long-term photocatalytic H_2_O_2_ evolution test.


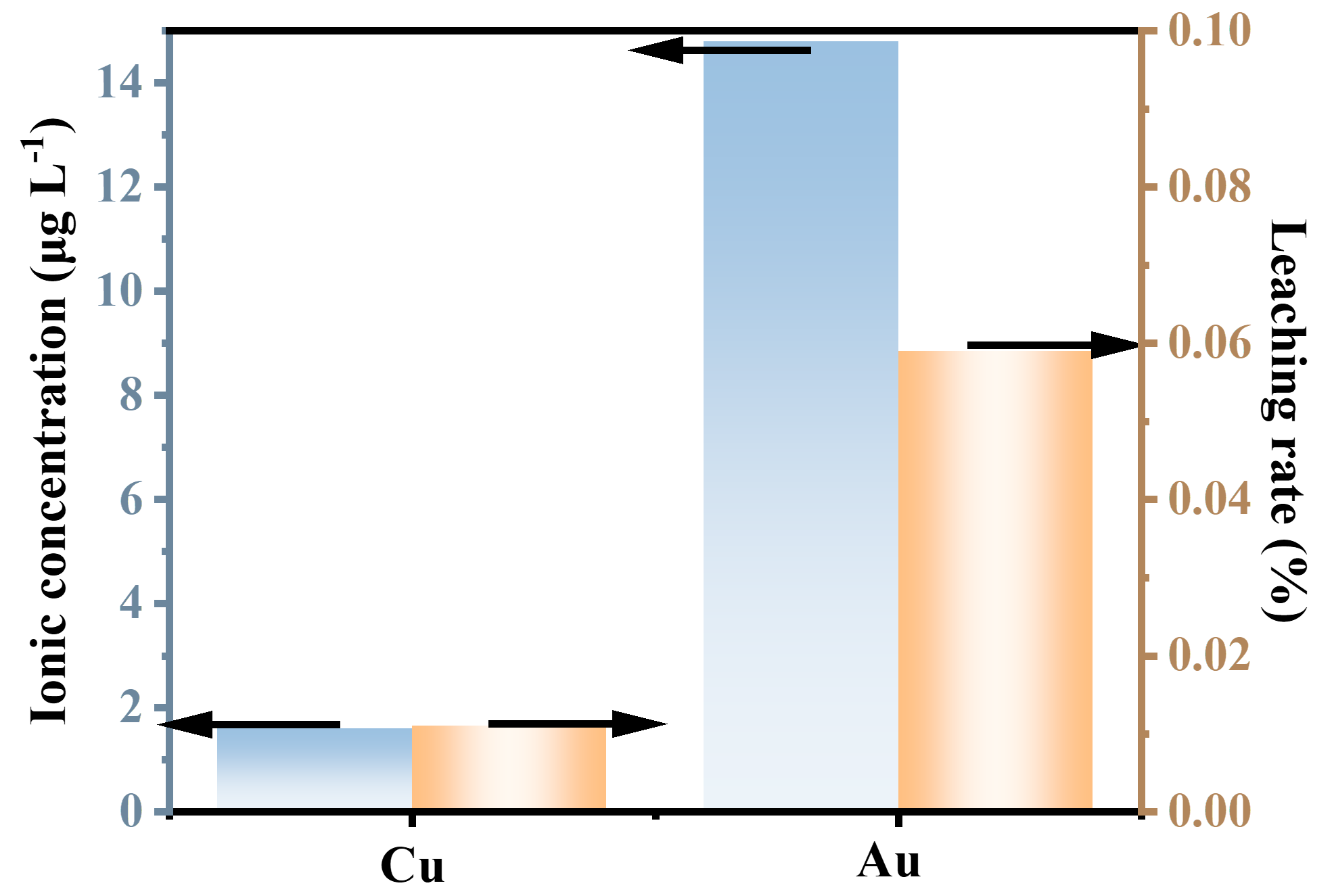


**Figure S16** Concentrations and leaching rates of Cu and Au in solution after photocatalytic reactions (Conditions: 100 mg catalyst, 40 ml water, light for 3 h. According to the ICP results, the contents of Cu and Au in Au/Cu-d/ZIS were 0.57% and 0.99% respectively).


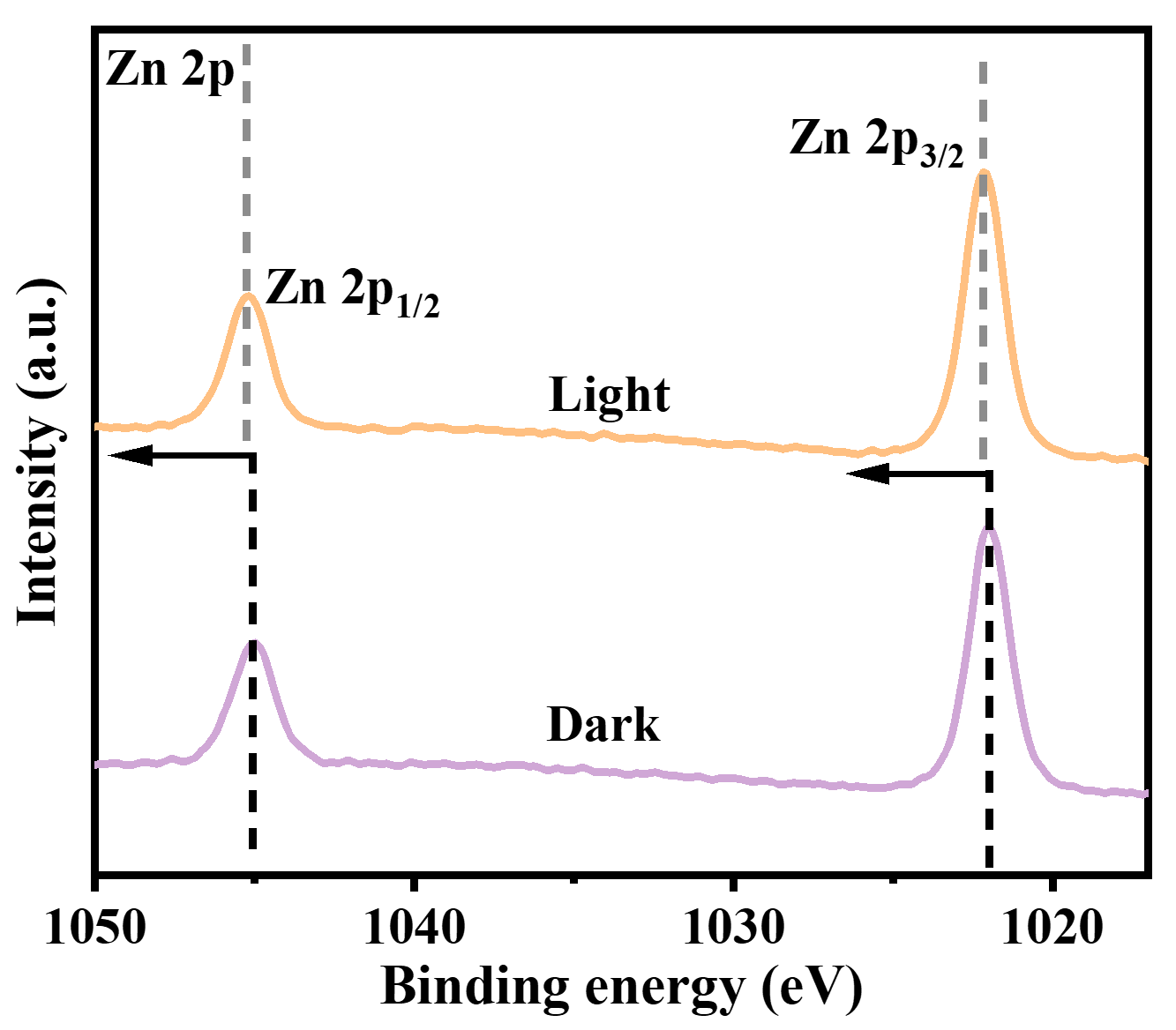


**Figure S17** In-situ XPS spectra of Zn 2p for Au/Cu-d/ZIS.


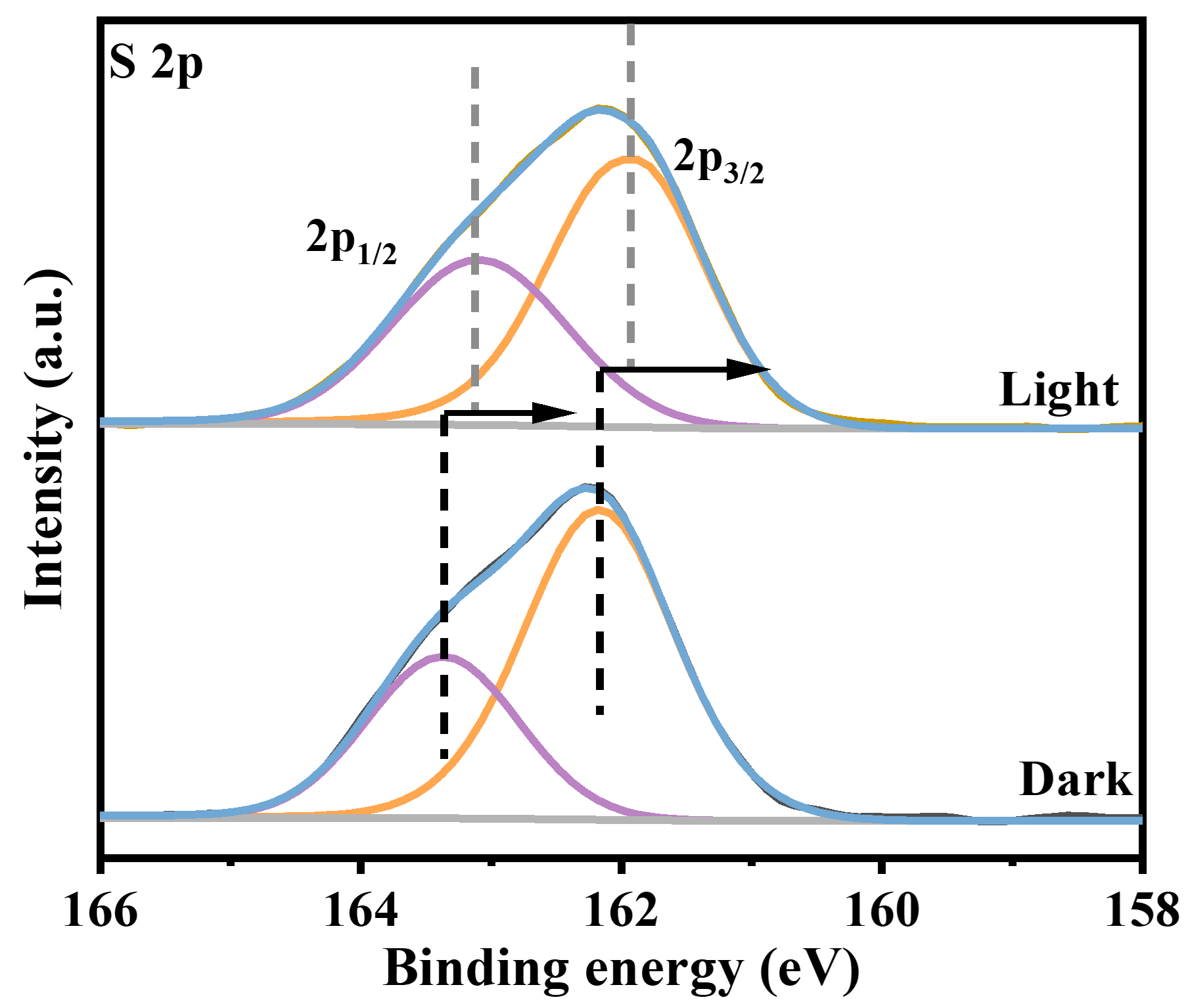


**Figure S18** In-situ XPS spectra of S 2p for Au/Cu-d/ZIS.


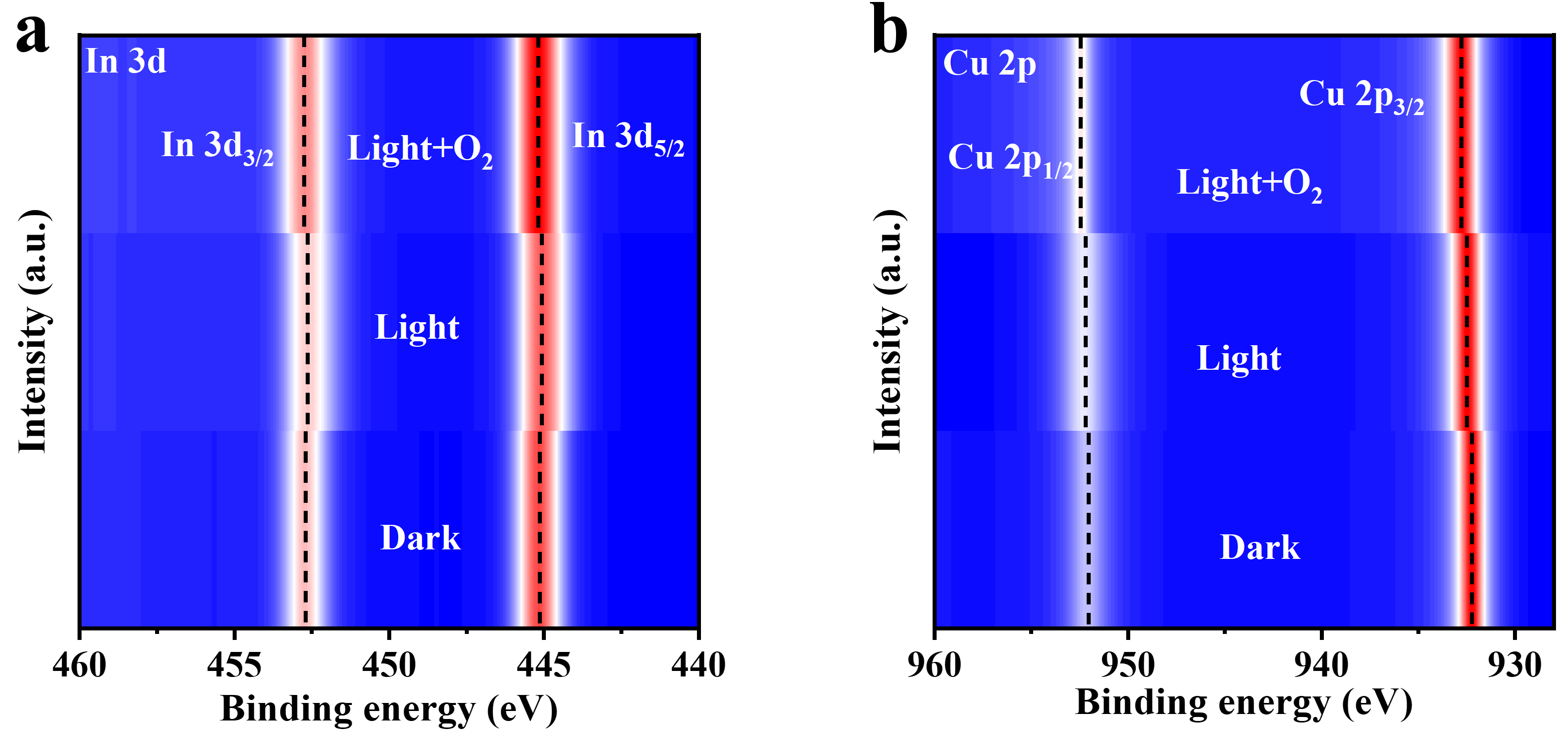


**Figure S19** In-situ XPS spectra of (a) In 3d, (b) Cu 2p under dark and irradiation of Au/Cu-d/ZIS catalyst.


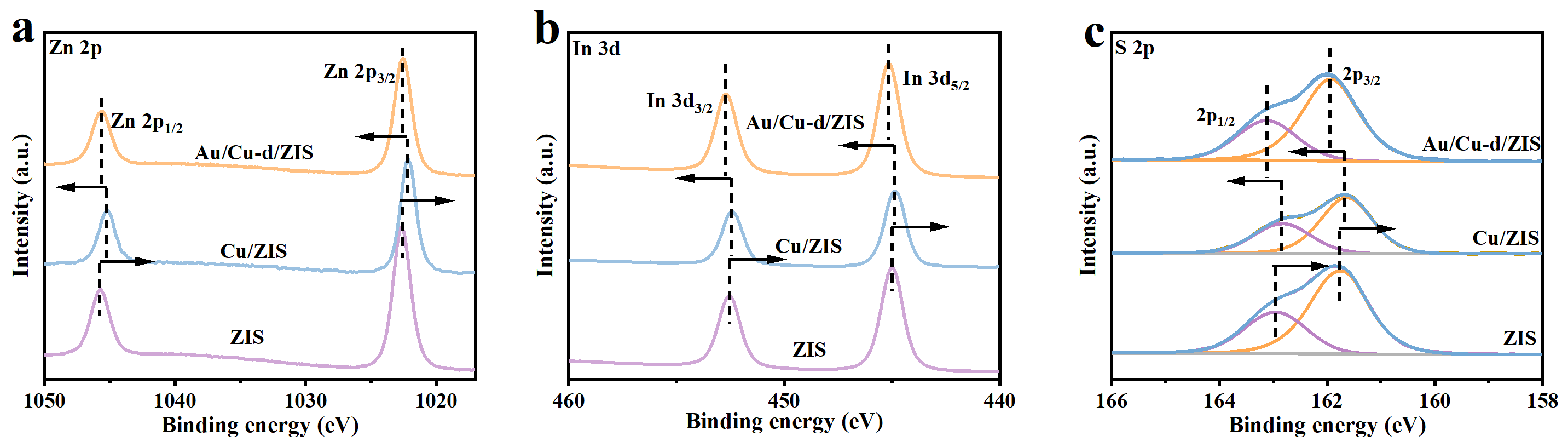


**Figure S20** (a) Zn 2p, (b) In 3d and (c) S 2p XPS spectra of samples.


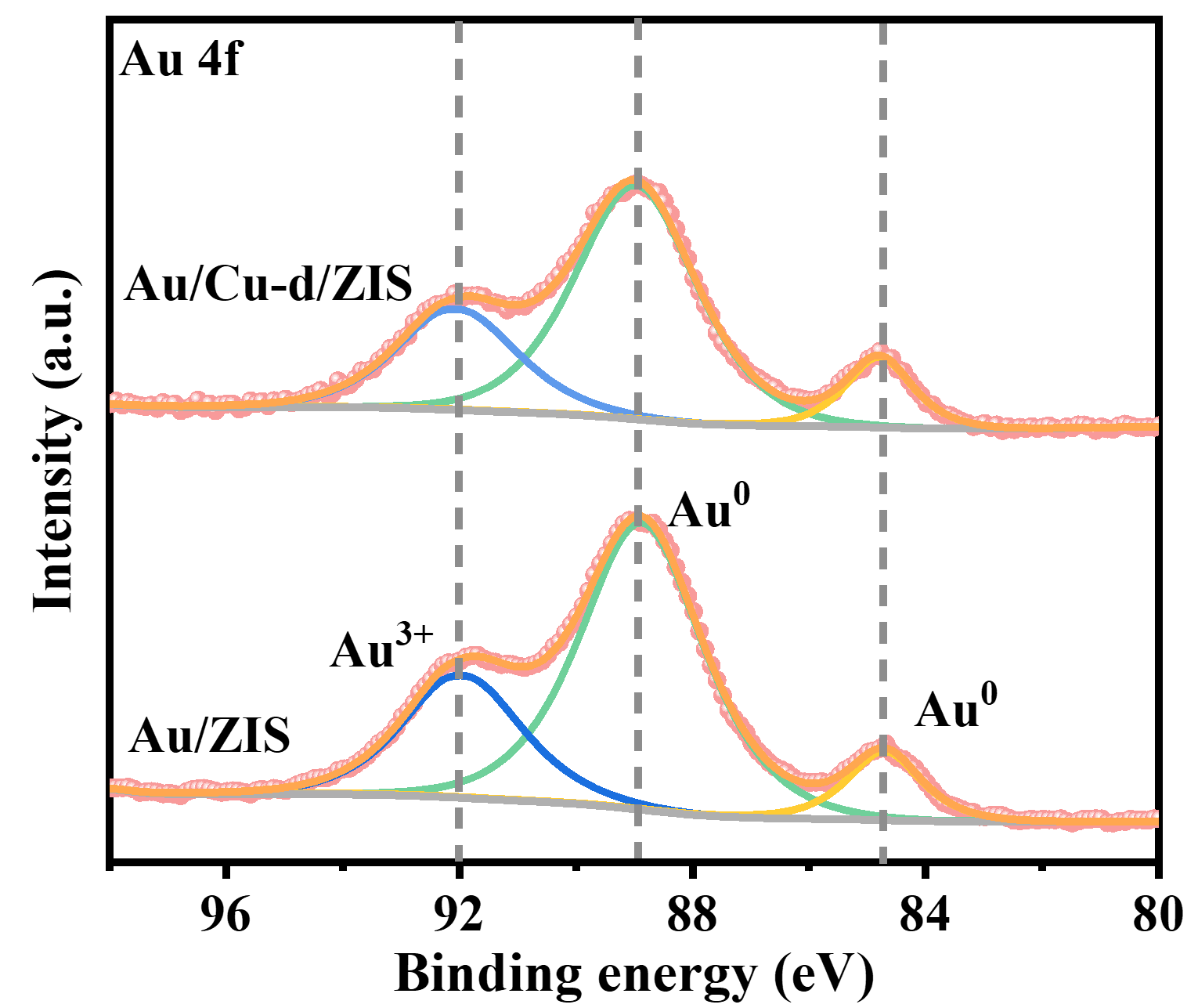


**Figure S21** Au 4f XPS spectra of samples.


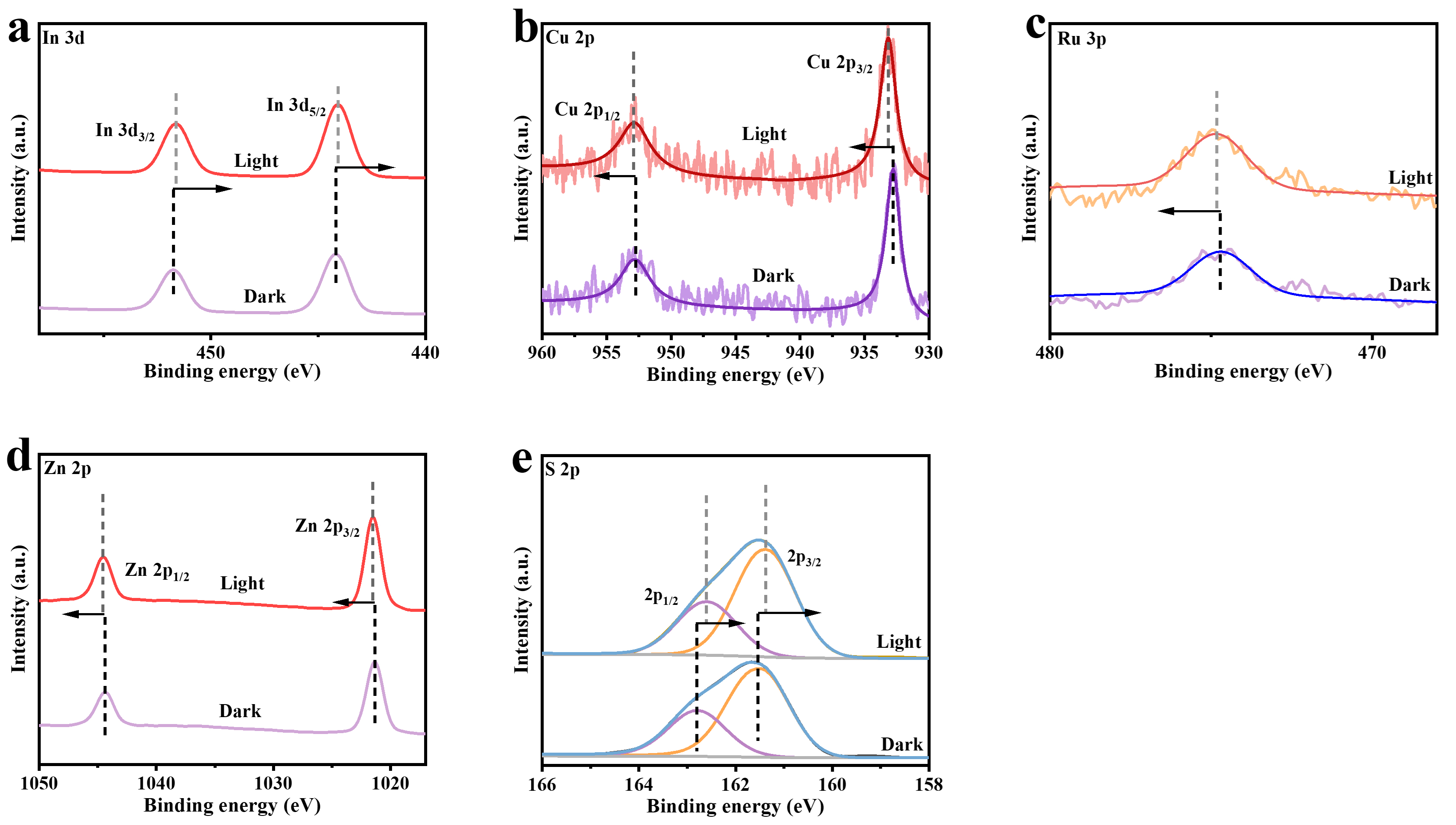


**Figure S22** In-situ XPS spectra of (a) In 3d, (b) Cu 2p, (c) Ru 3p, (d) Zn 2p and (e) S 2p under dark and irradiation of Ru/Cu-d/ZIS catalyst.


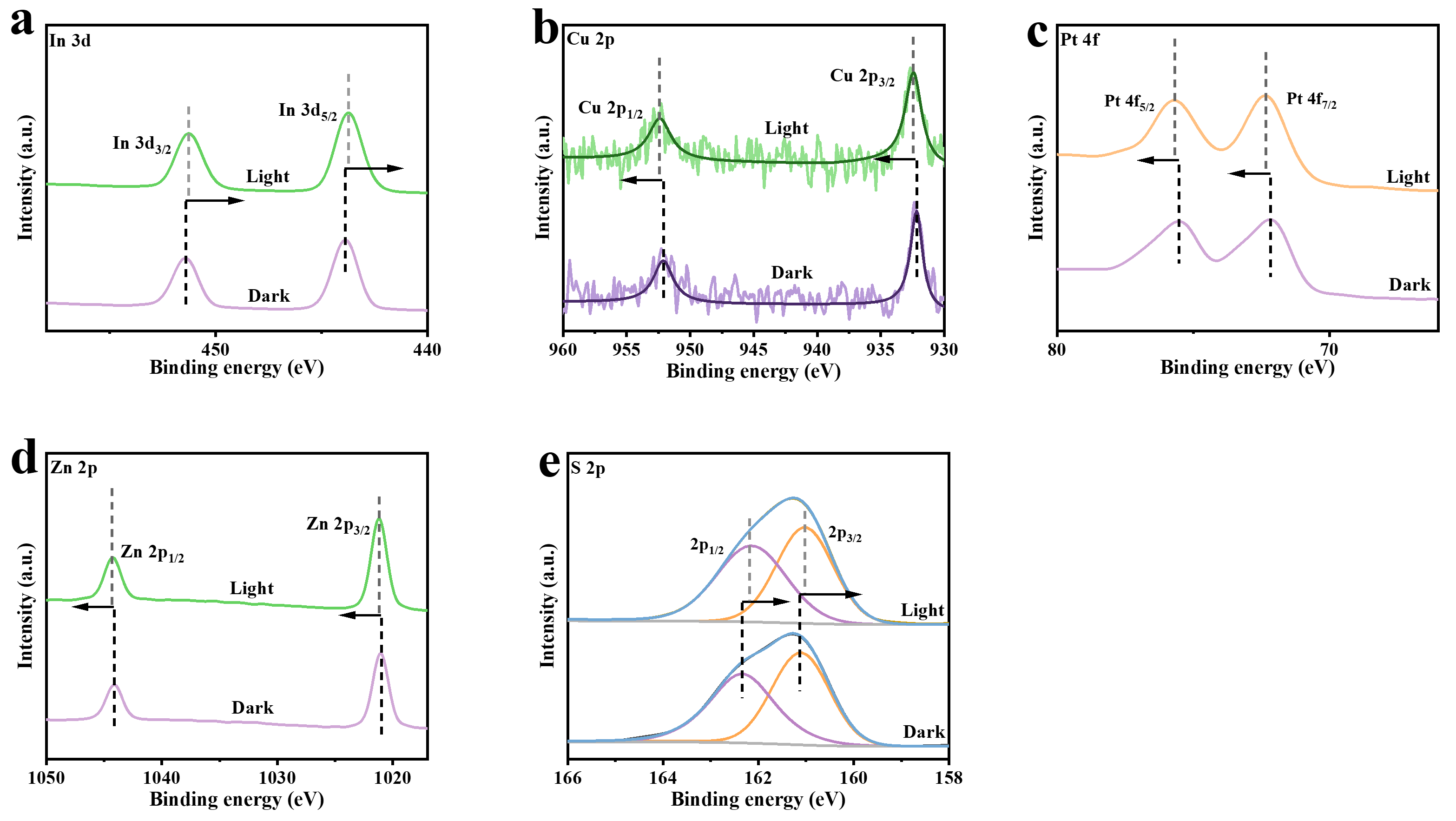


**Figure S23** In-situ XPS spectra of (a) In 3d, (b) Cu 2p, (c) Pt 4f, (d) Zn 2p and (e) S 2p under dark and irradiation of Pt/Cu-d/ZIS catalyst.


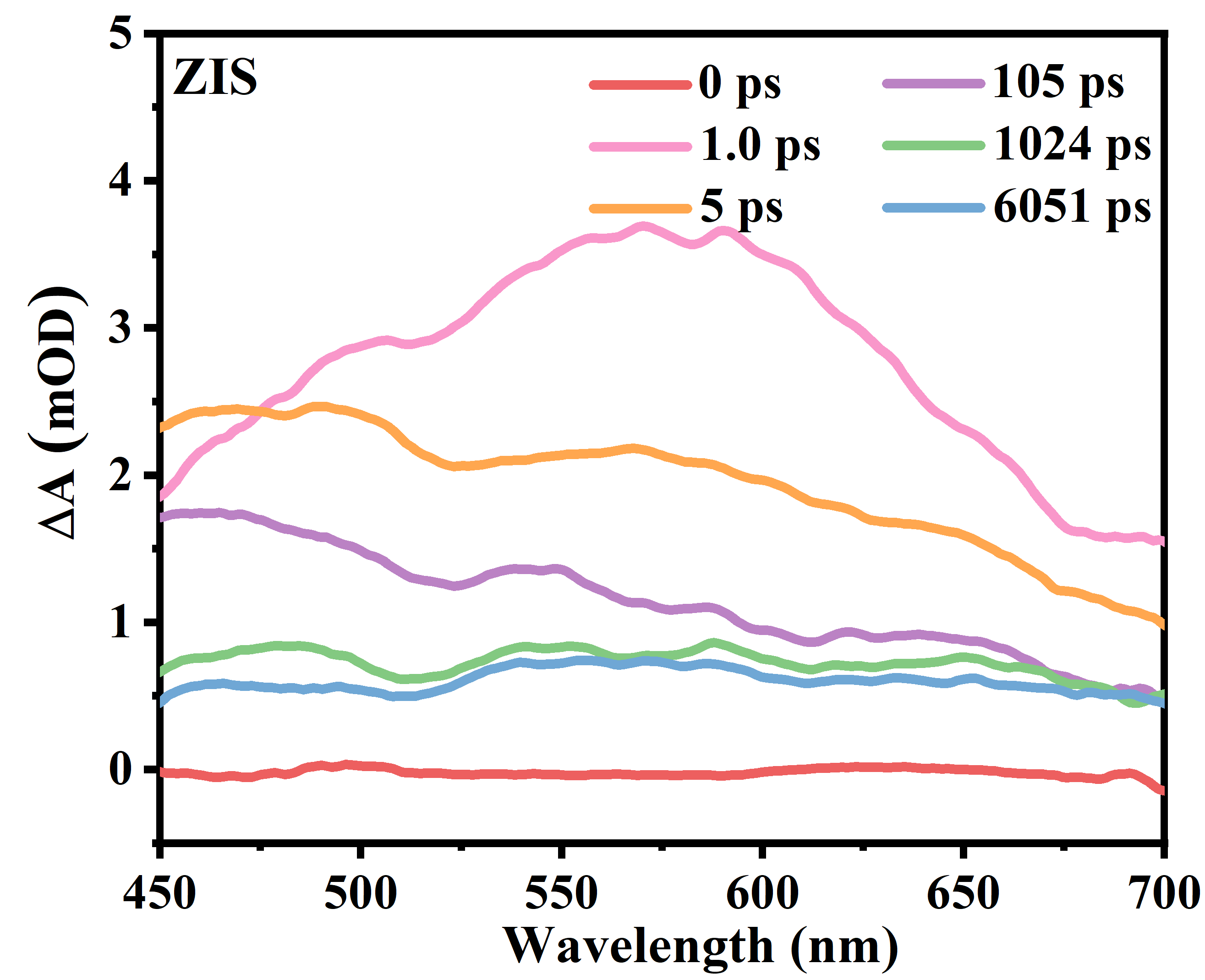


**Figure S24** Transient absorption spectra of ZIS.


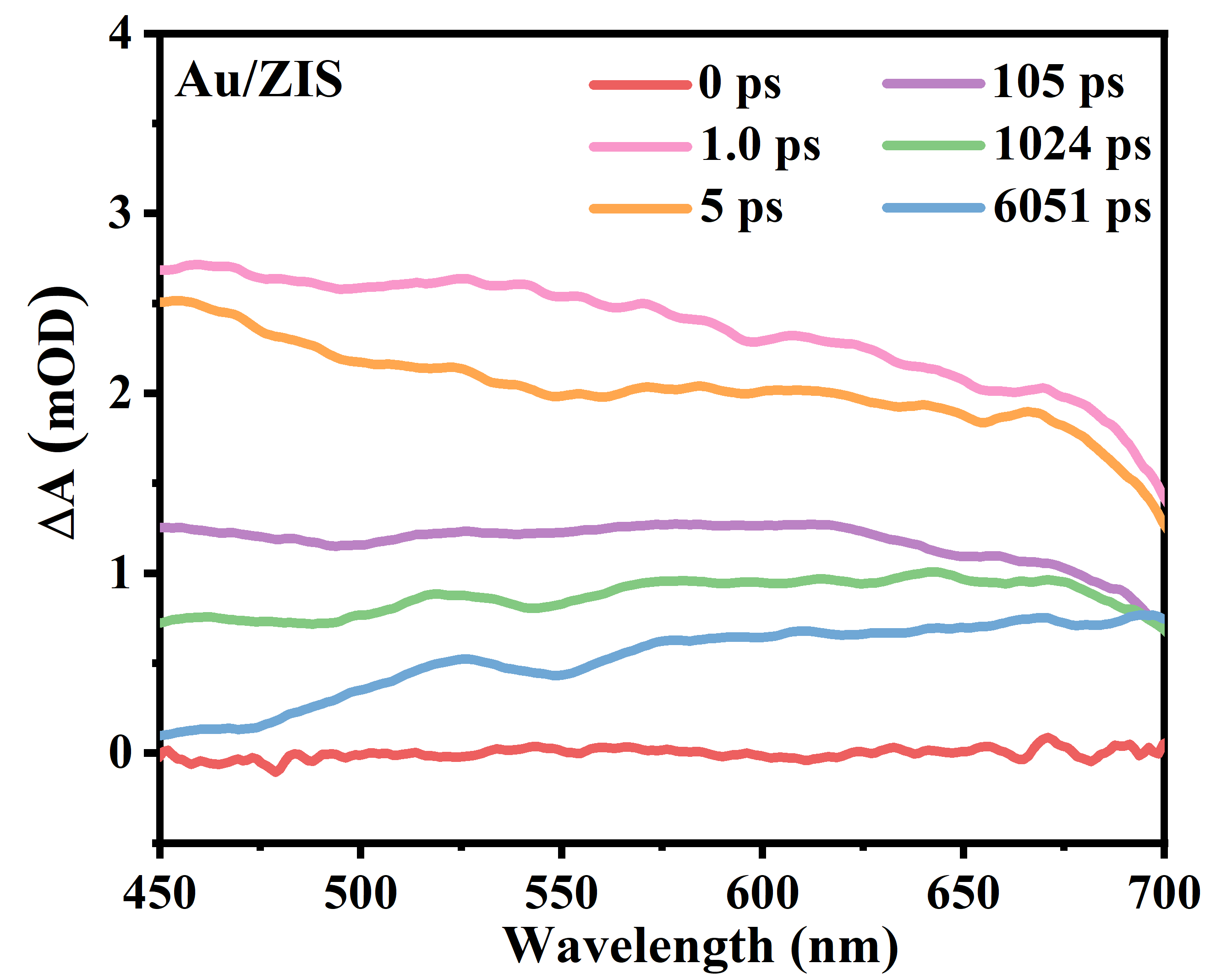


**Figure S25** Transient absorption spectra of Au/ZIS.


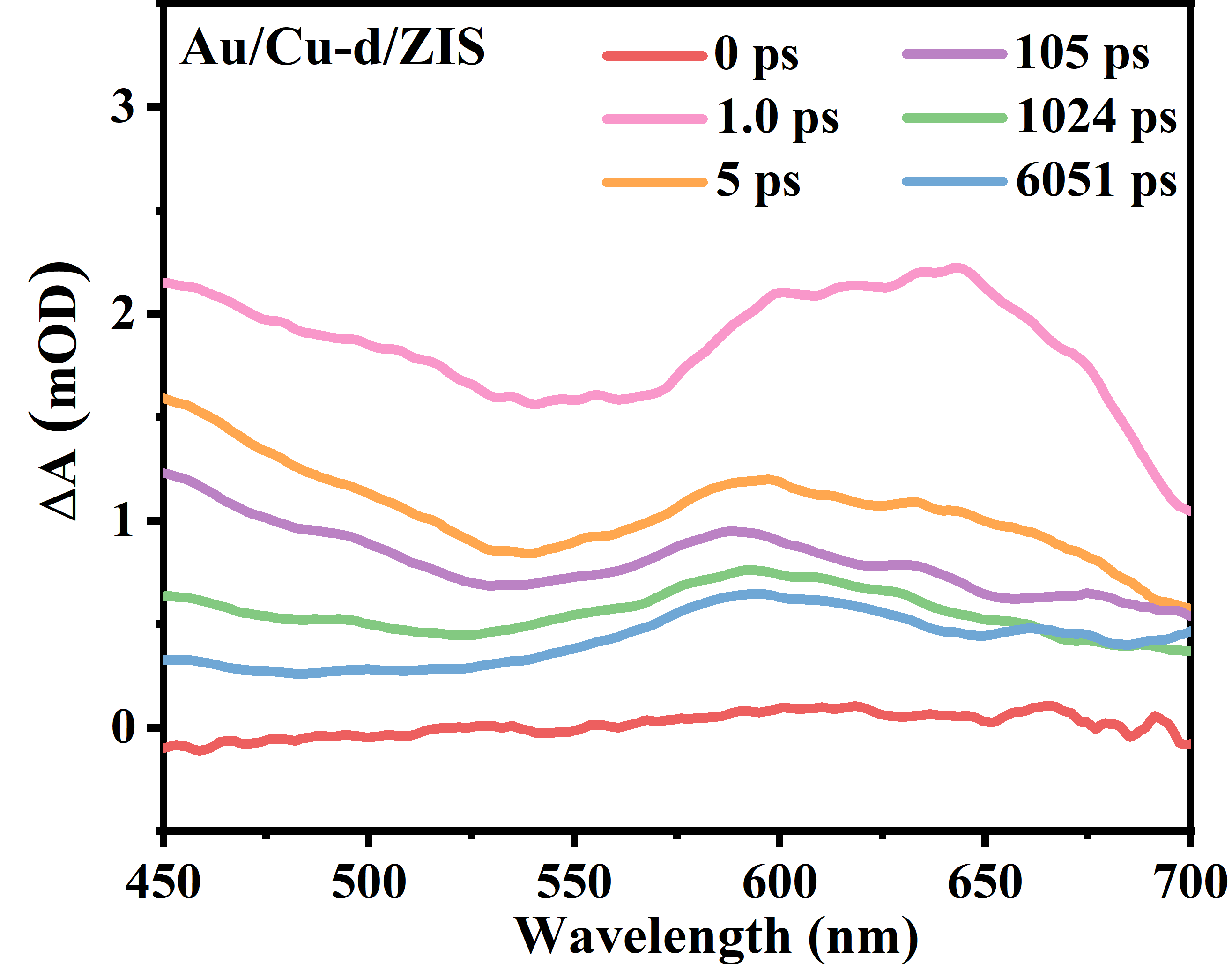


**Figure S26** Transient absorption spectra of Au/Cu-d/ZIS.


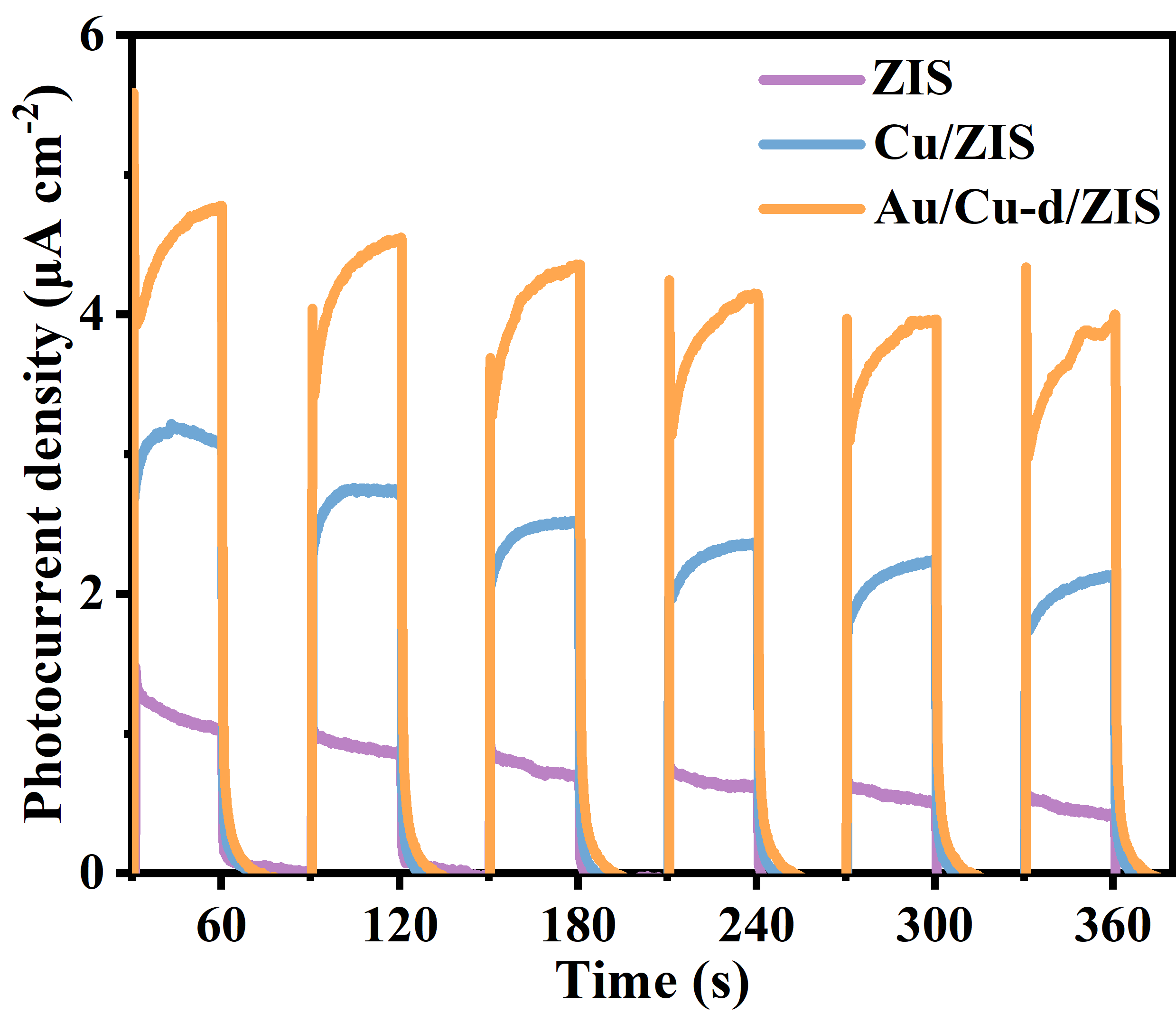


**Figure S27** Transient photocurrent response of ZIS, Cu/ZIS and Au/Cu-d/ZIS.


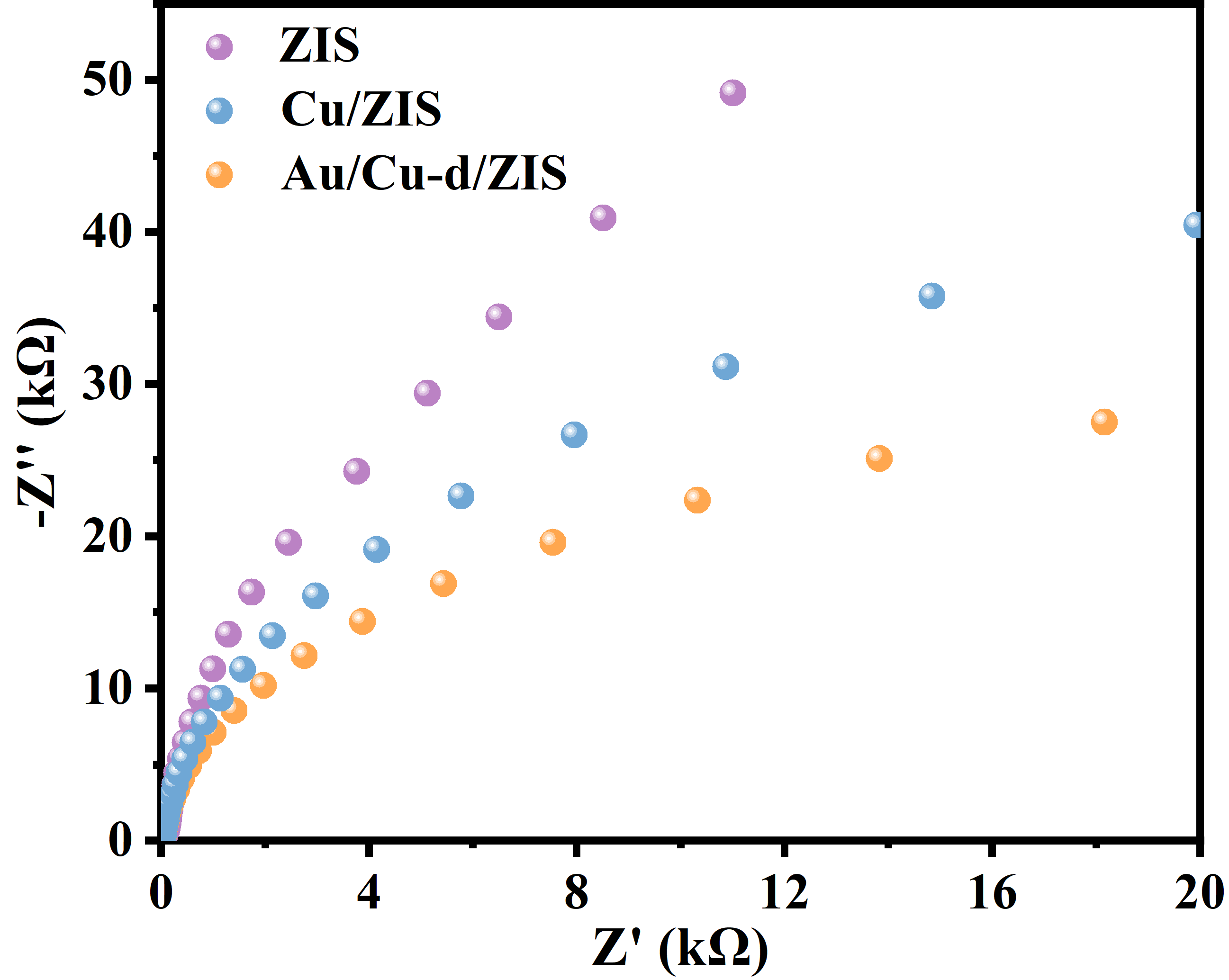


**Figure S28** Electrochemical impedance spectroscopy of ZIS, Cu/ZIS and Au/Cu-d/ZIS.


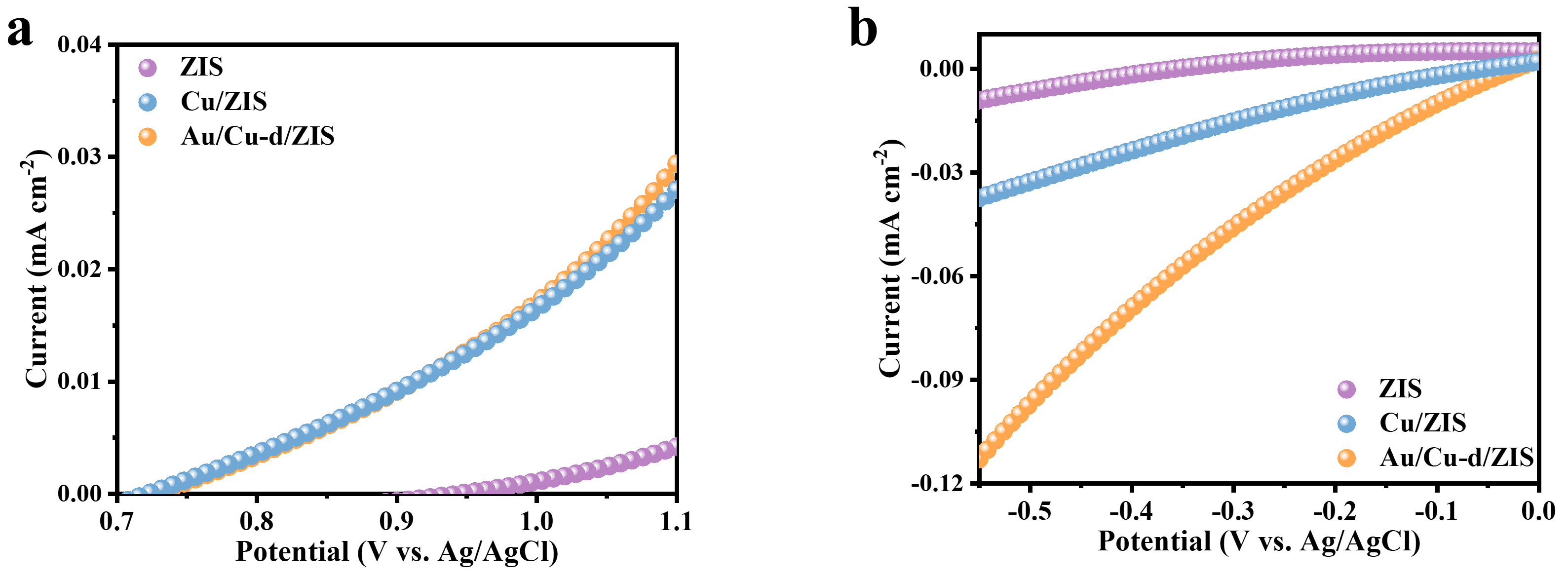


**Figure S29** Negative sweep LSV current curves (a) and positive sweep LSV current curves (b) of samples under light.


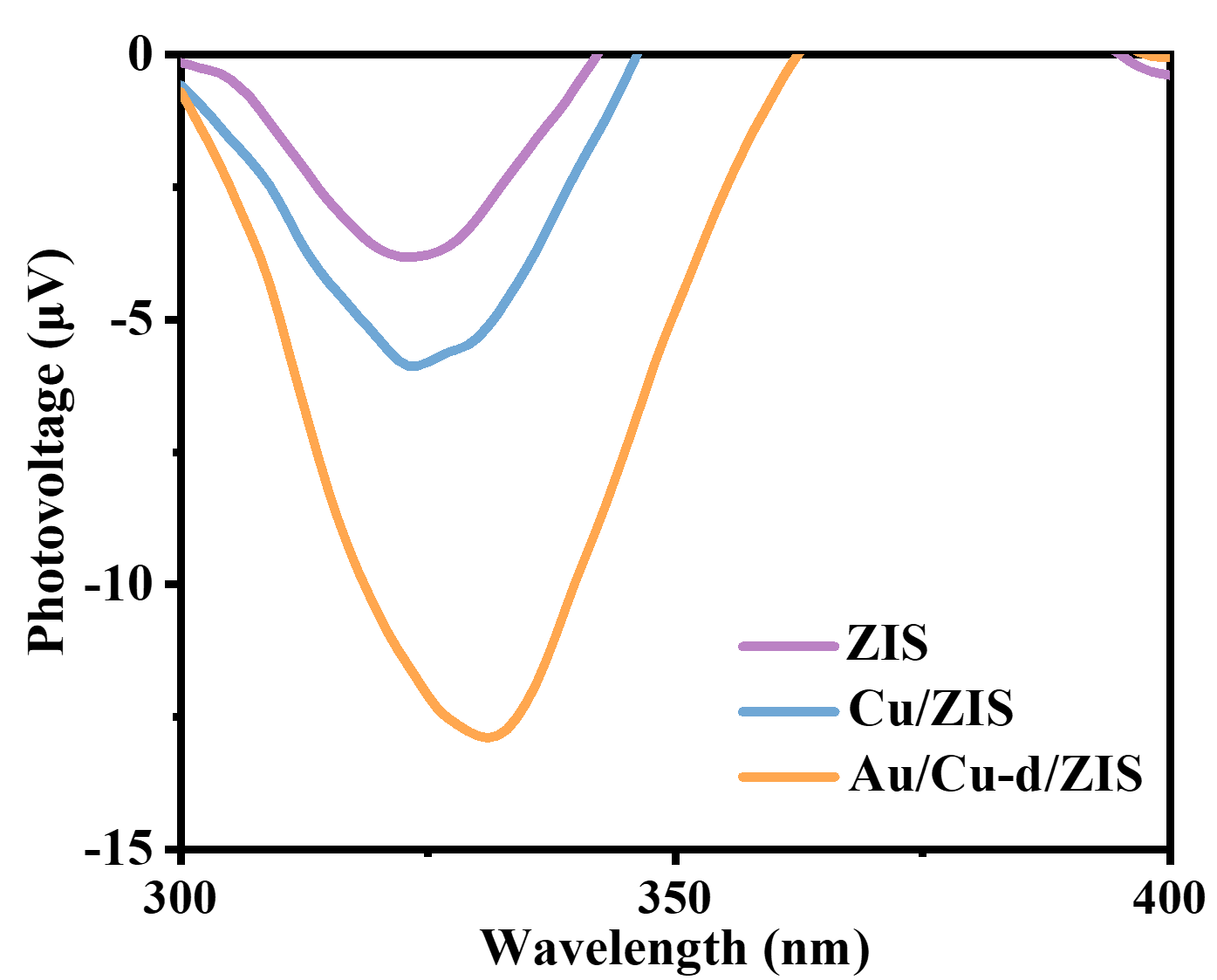


**Figure S30** Steady-state surface photovoltage (SPV) of as-prepared catalysts.


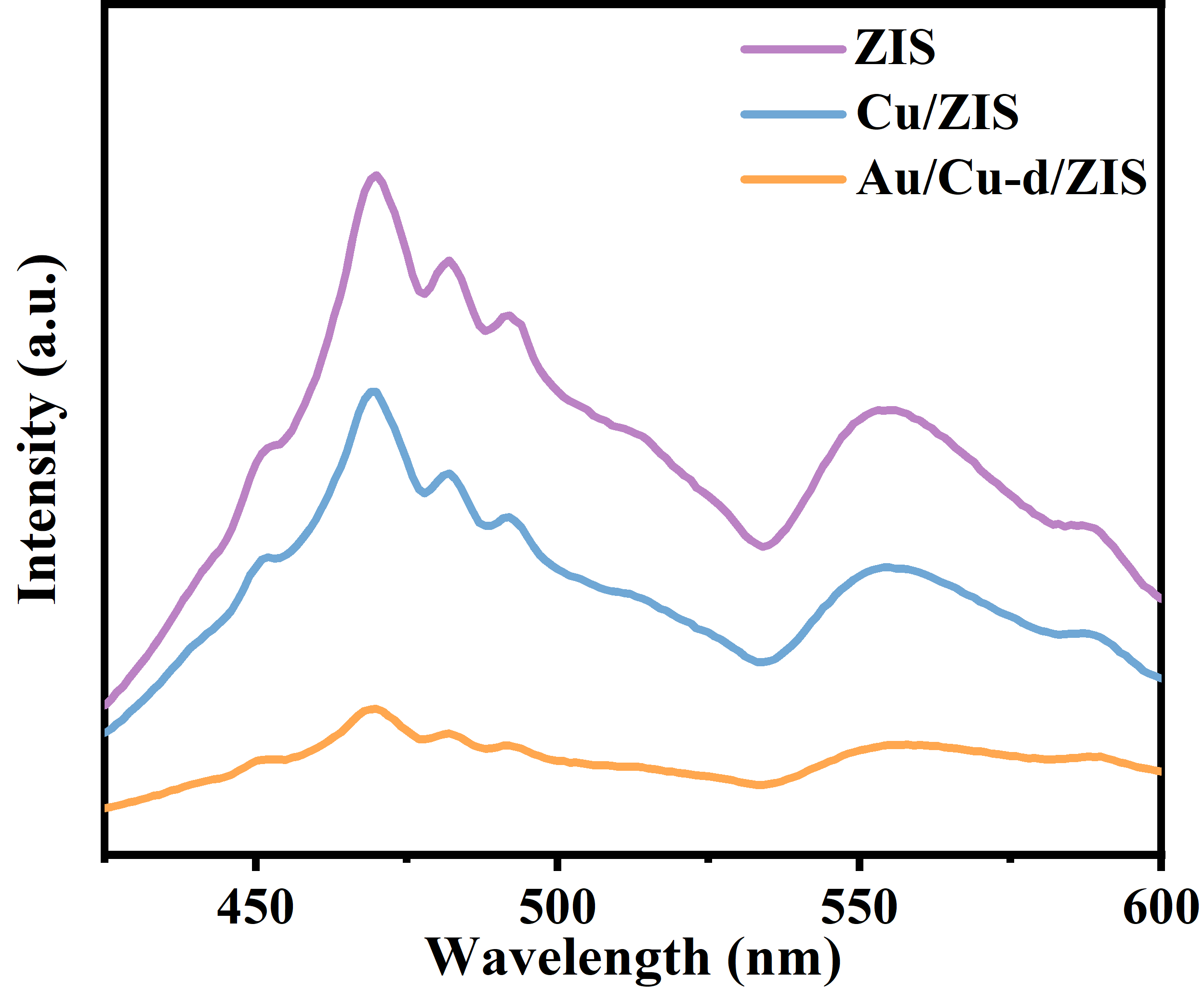


**Figure S31** Photoluminescence spectrum of ZIS, Cu/ZIS and Au/Cu-d/ZIS.


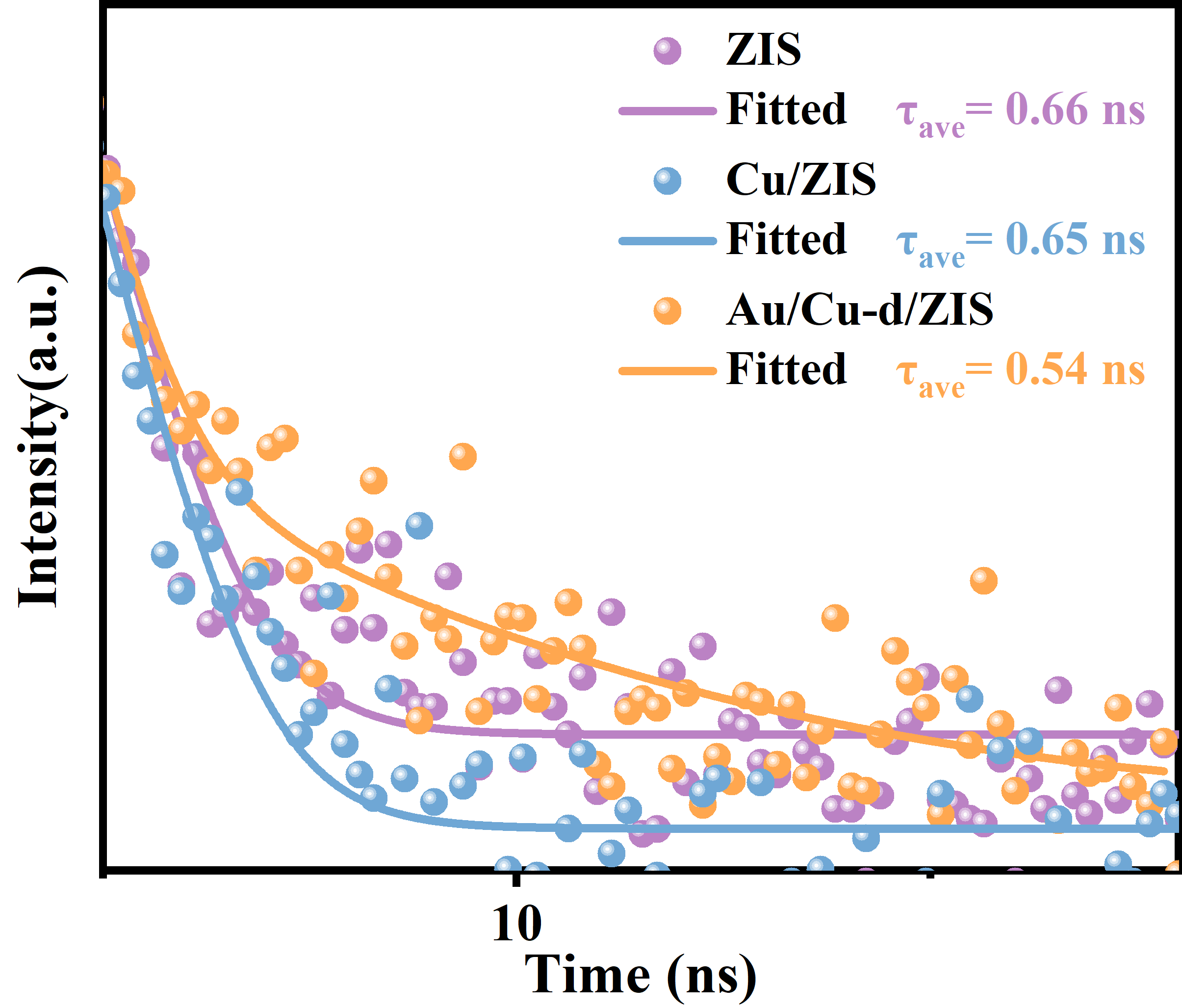


**Figure S32** TRPL of ZIS, Cu/ZIS and Au/Cu-d/ZIS.


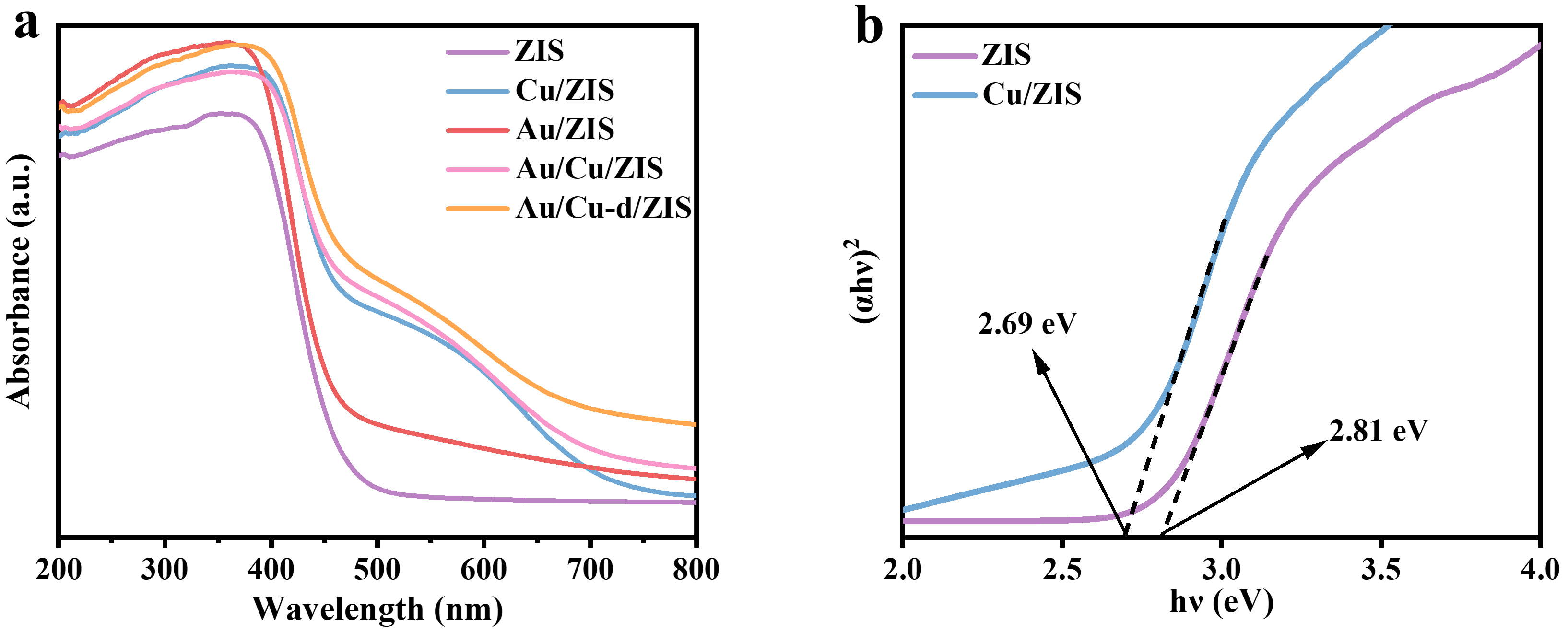


**Figure S33** (a) Ultraviolet-visible absorption spectra of samples. (b) Tauc plots of ZIS and Cu/ZIS.


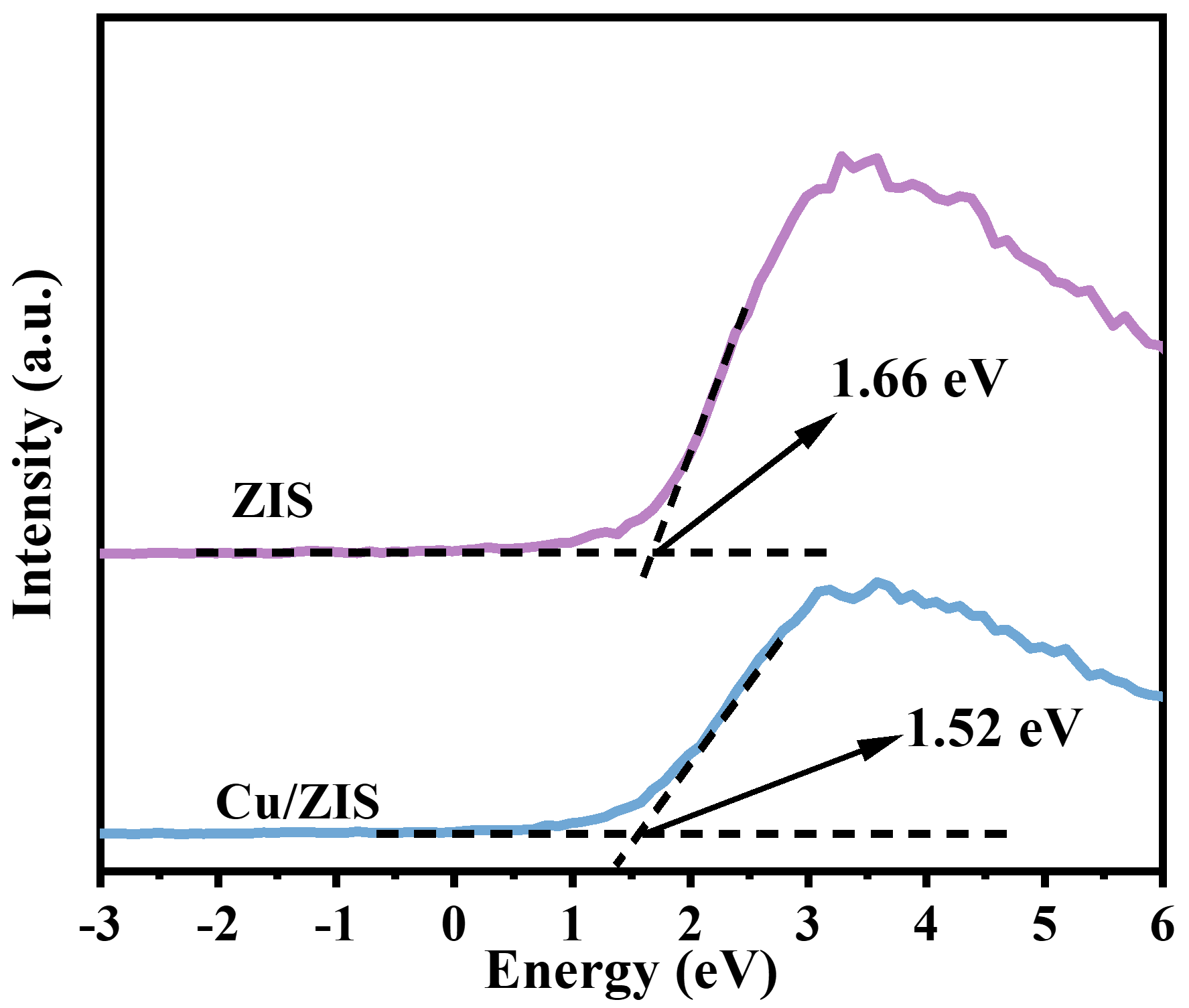


**Figure S34** VB-XPS spectra of ZIS and Cu/ZIS.


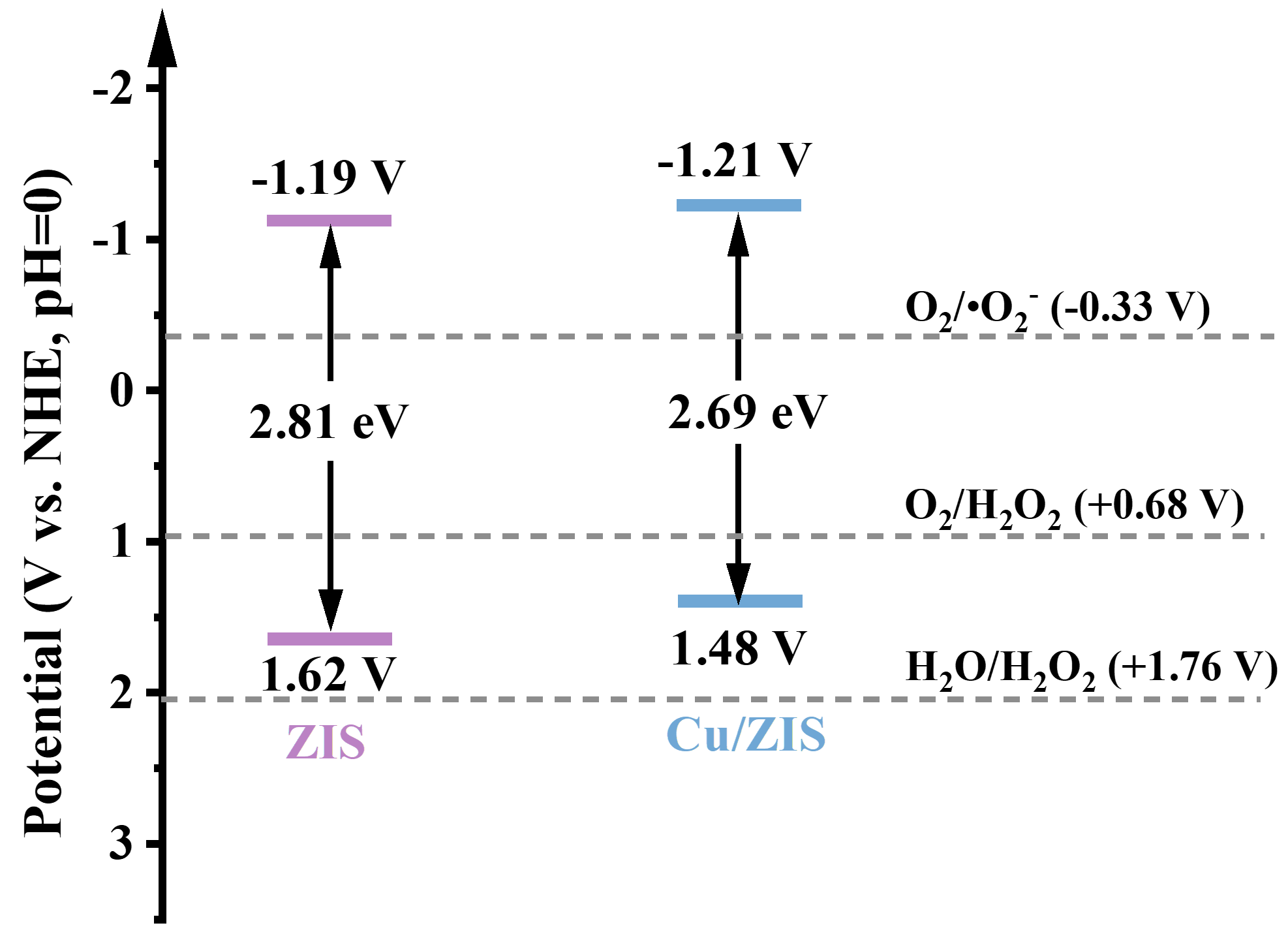


**Figure S35** Energy band diagrams of ZIS and Cu/ZIS.


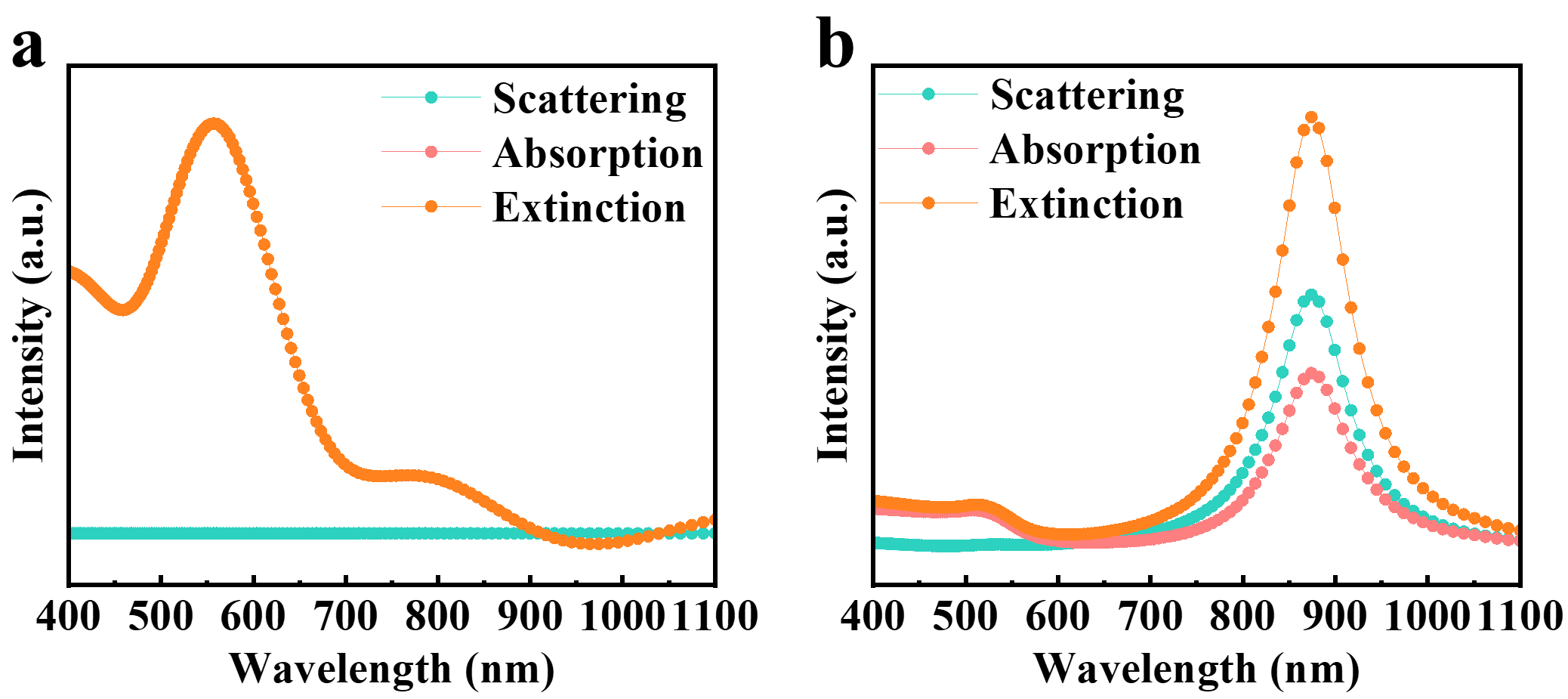


**Figure S36** Calculated extinction, adsorption and scattering fraction of (a) Au NPs (2.6 nm), (b) Au NRs.


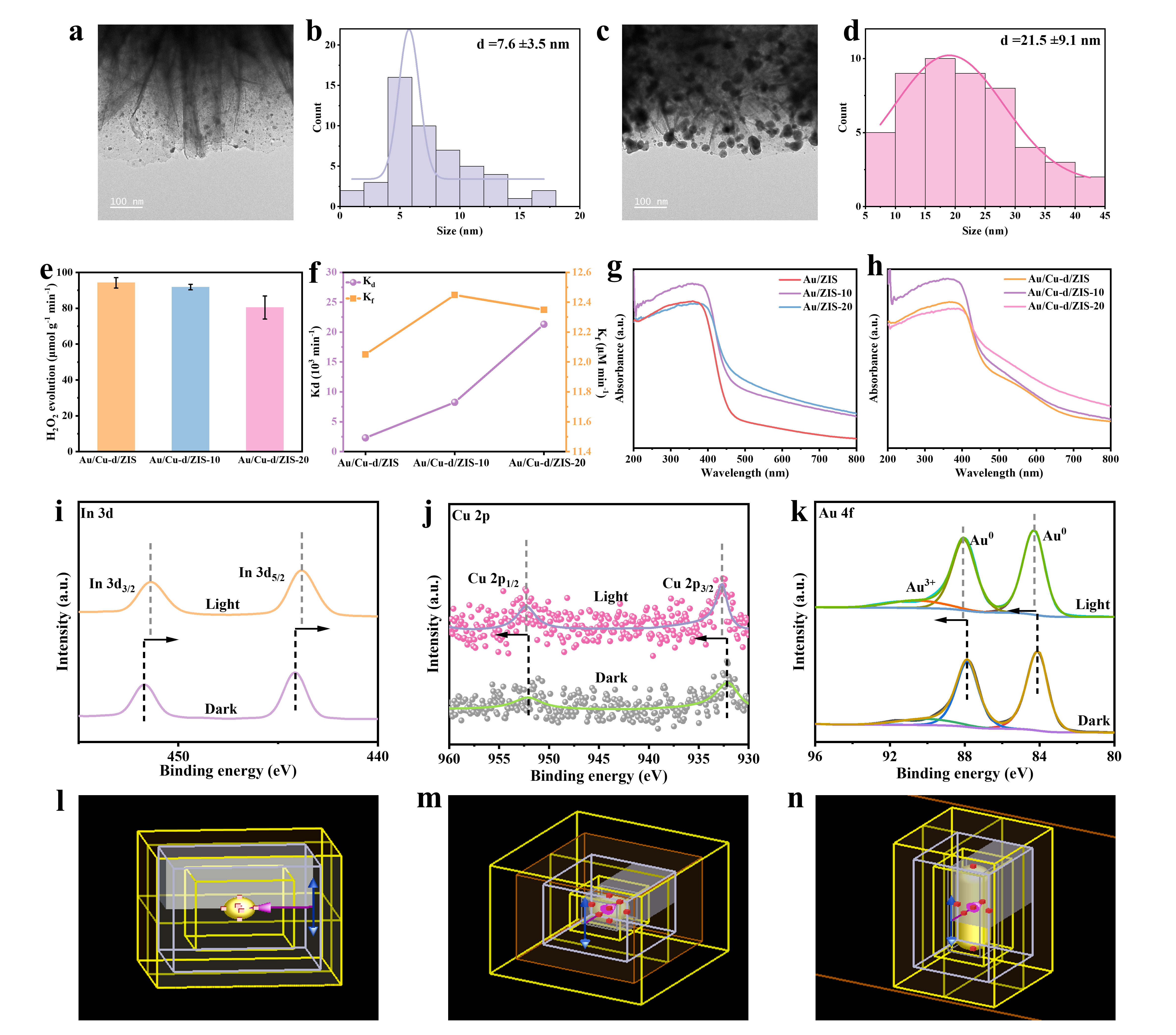


**Figure S37** (a) Transmission electron microscopy of (a) Au/Cu-d/ZIS-10, (c) Au/Cu-d/ZIS-20 and (b and d) particle size statistics of Au. (e) Photocatalytic H_2_O_2_ evolution of photocatalysts. (f) Formation rate constant (*K*_f_) and decomposition rate constant (*K*_d_) of H_2_O_2_. (g, h) Ultraviolet-visible absorption spectra of samples. In-situ XPS spectra of (i) In 3d, (j) Cu 2p, (k) Au 4f under dark and irradiation of Au/Cu-d/ZIS-20 catalyst. The FDTD model of (l) Au NPs (2.6 nm), (m) Au NPs (21 nm) and (n) Au nanorods.


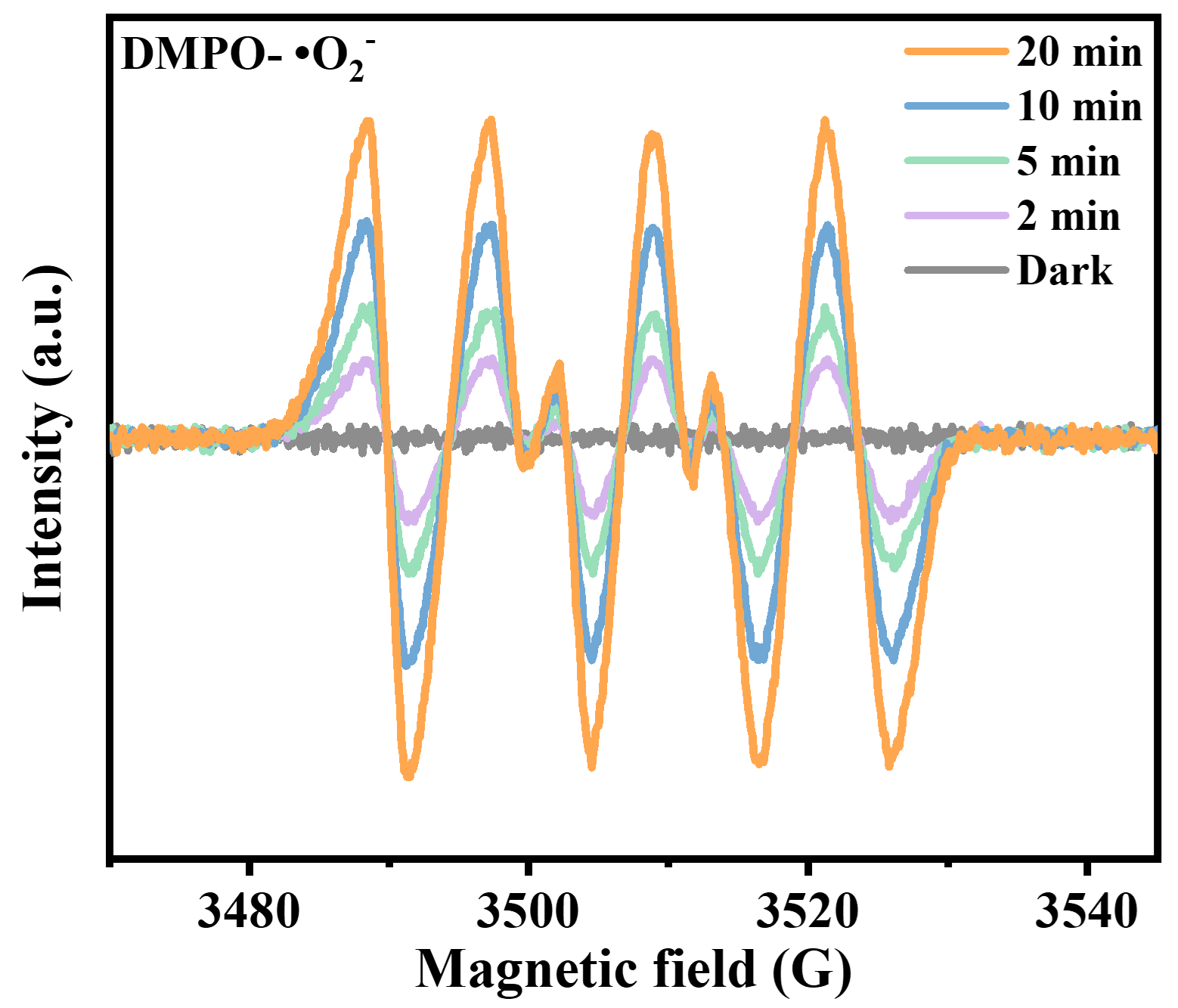


**Figure S38** DMPO spin-trapping ESR spectra for •O_2_^−^ in methanol of Au/Cu-d/ZIS with different time.


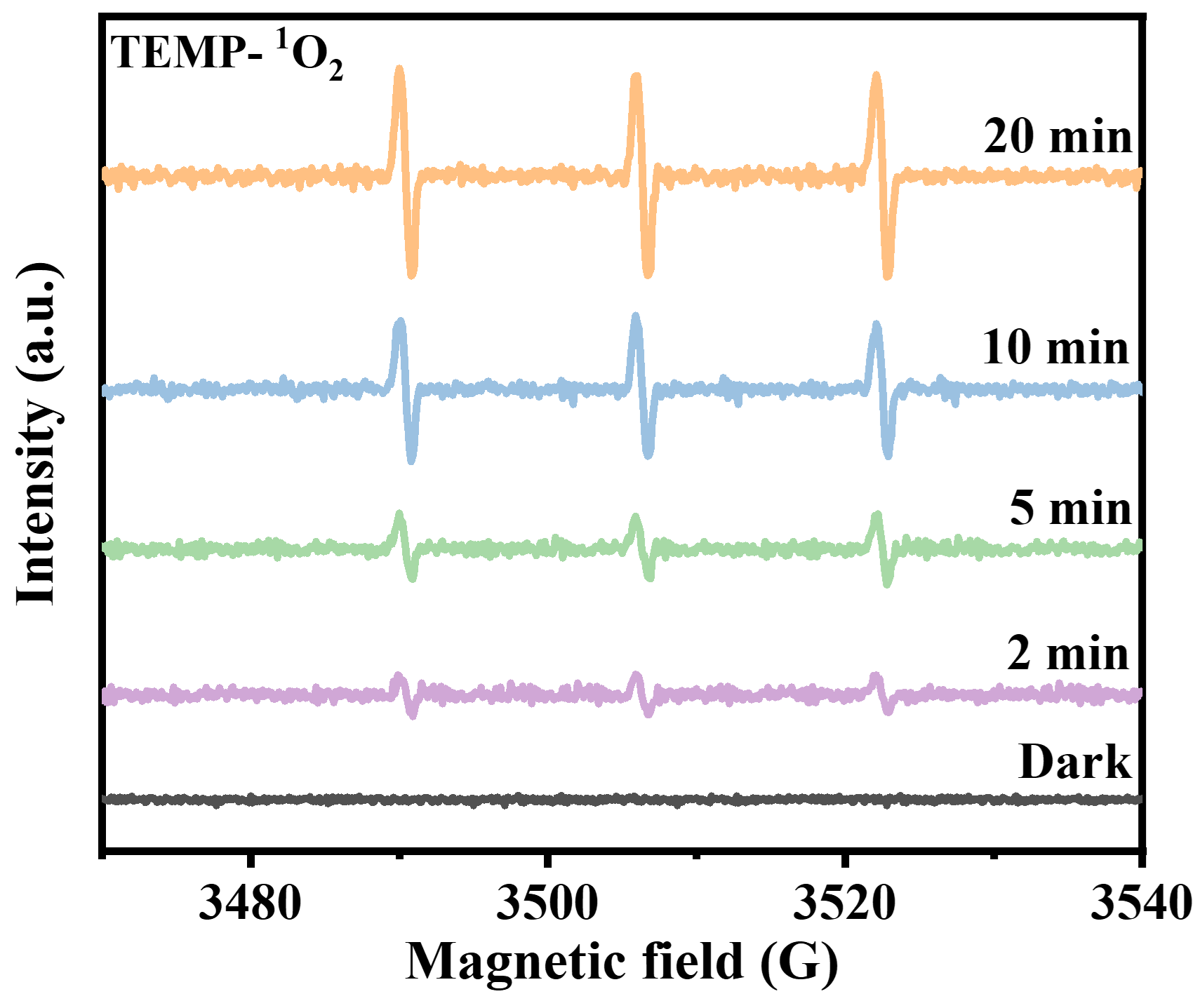


**Figure S39** TEMP spin-trapping ESR spectra for ^1^O_2_ of Au/Cu-d/ZIS with different times.


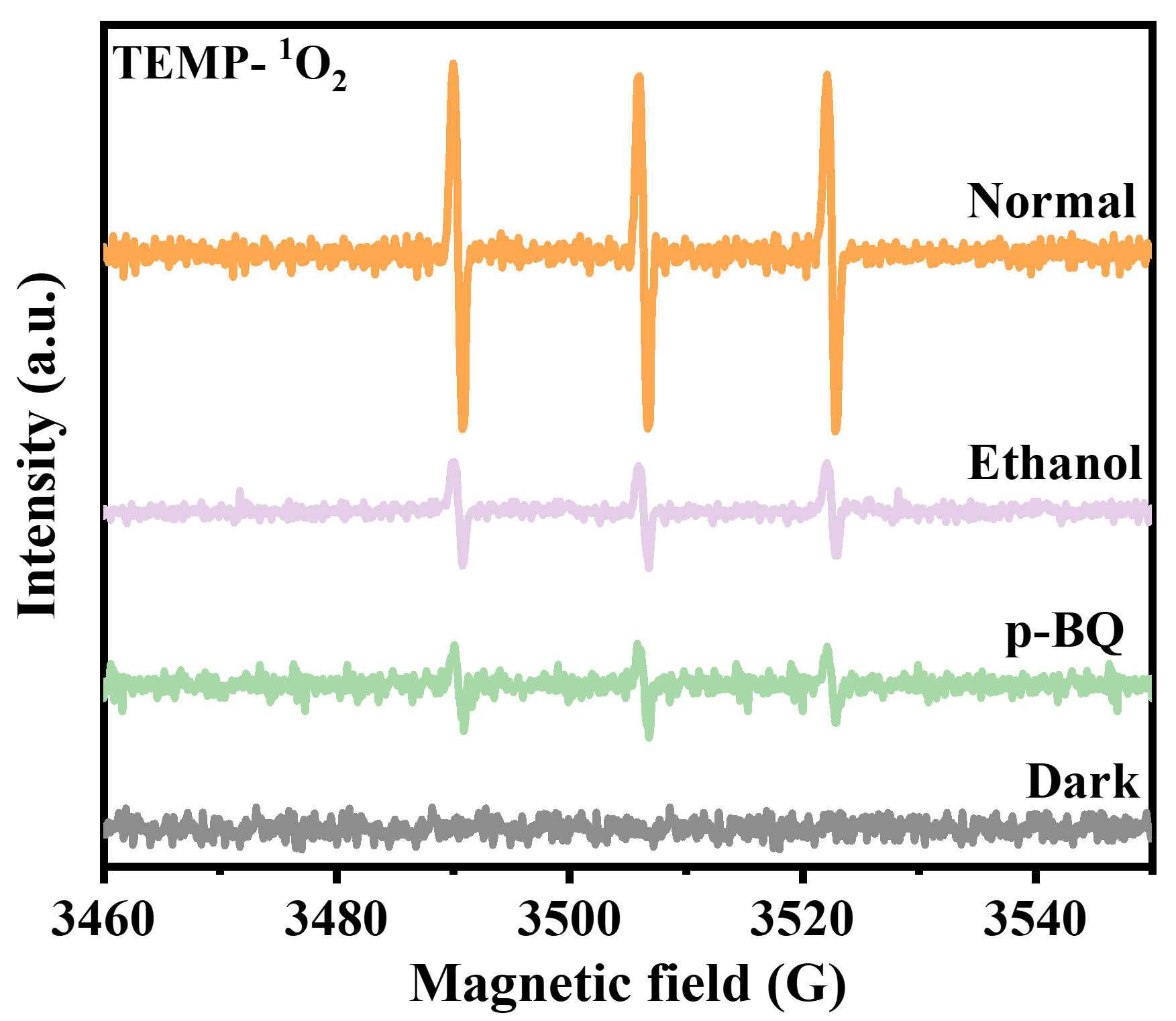


**Figure S40** TEMP spin-trapping ESR spectra for ^1^O_2_ of Au/Cu-d/ZIS with different conditions.


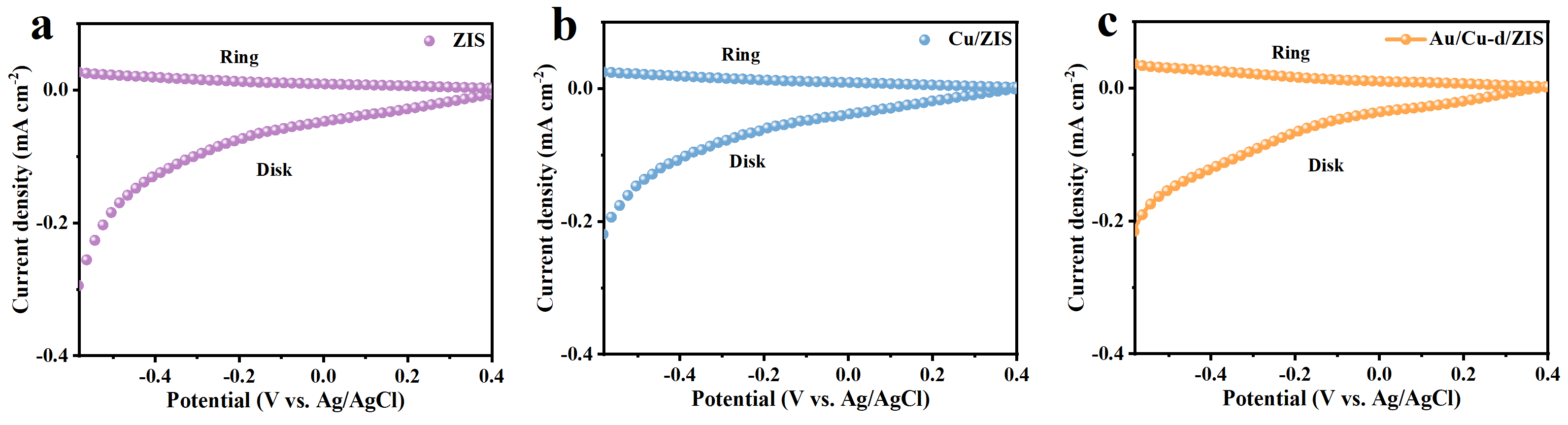


**Figure S41** RRDE curves of ring current and disk current density of (a) ZIS, (b) Cu/ZIS, and (c) Au/Cu-d/ZIS.


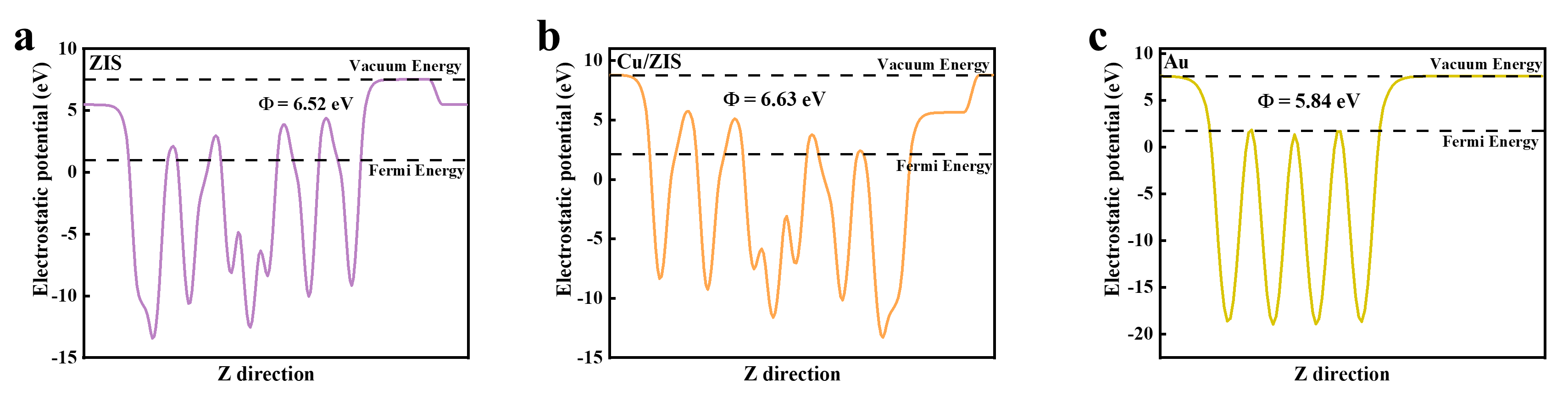


**Figure S42** Calculated electrostatic potentials for (a) ZIS, (b) Cu/ZIS and (c) Au.

1. **Table. S1**

**Table S1** Comparison of the performance with other organic/inorganic photocatalysts for photocatalytic production of H_2_O_2_ (no sacrificial agent).

| **Photocatalysts** | **Light source** | **Gas** | **Yield (μmol·g^−1^·min^−1^**) | **Ref.** |
| --- | --- | --- | --- | --- |
| **Inorganic photocatalysts** | | | | |
| **Au/Cu-d/ZIS** | AM 1.5G | **O_2_** | **94.2** | **This work** |
| CoO_x_/Mo:BiVO_4_/Pd | AM 1.5G | O_2_ | 11.9 | ^9^ |
| TiO_2_ | Hg lamp (500 W) | - | 54.8 | ^10^ |
| Pd/A/BiVO_4_ | 300 W Xe lamp (λ≥420 nm) | O_2_ | 13.4 | ^11^ |
| BiPO_4_ | 18 W UV lamp | O_2_ | 10.0 | ^12^ |
| CdS/K_2_Ta_2_O_6_ | λ≥420 nm | Air | 2.7 | ^13^ |
| ZIS@BVO  Sv-ZIS  Sv-CdS  TpMA/In_2_S_3_  CdS/ZnIn_2_S_4_ | λ≥420 nm  λ≥400 nm  Full spectrum  λ≥420 nm  λ≥400 nm | O_2_  O_2_  O_2_  O_2_  - | 30.0  28.4  27.2  17.3  10.1 | ^14^  ^15^  ^16^  ^17^  ^18^ |
| **Organic photocatalysts** | | | | |
| o-COF-TpPzda | λ>420 nm | O_2_ | 73.3 | ^19^ |
| PM-CDs-30 | λ≥420 nm | O_2_ | 29.6 | ^20^ |
| TD-COF | λ=400-700 nm | O_2_ | 67.7 | ^21^ |
| SA-TCPP | λ≥420 nm | O_2_ | 19.2 | ^22^ |
| Ni_SAPs_-PuCN | λ≥420 nm | O_2_ | 5.7 | ^23^ |
| TB-COF | λ=400-700 nm | O_2_ | 86.4 | ^24^ |
| COF-2CN | λ≥420 nm | O_2_ | 81.0 | ^25^ |
| CuBr-dptz | λ≥400 nm | O_2_ | 44.8 | ^26^ |
| Co_SA_/Py-CTF | λ≥420 nm | O_2_ | 48.3 | ^27^ |
| H_2_Pc-THHI-COF | λ≥400 nm | O_2_ | 75.2 | ^28^ |

**11. References**

1. G. Kresse and J. Hafner, Ab initio molecular dynamics for liquid metals. *Phys. Rev. B.* 1993, **47**, 558.

2. G. Kresse and J. Furthmüller, Efficient iterative schemes for ab initio total-energy calculations using a plane-wave basis set. *Phys. Rev. B.* 1996, **54**, 11169.

3. P. E. Blöchl, Projector augmented-wave method. *Phys. Rev. B.* 1994, **50**, 17953.

4. G. Kresse and D. Joubert, From ultrasoft pseudopotentials to the projector augmented-wave method. *Phys. Rev. B.* 1999, **59**, 1758.

5. J. P. Perdew, K. Burke and M. Ernzerhof, Generalized Gradient Approximation Made Simple. *Phys*. *Rev*. *Lett*. 1996, **77**, 3865.

6. S. Grimme, Semiempirical GGA-type density functional constructed with a long-range dispersion correction. *J*. *Comput*. *Chem*. 2006, **27**, 1787.

7. J. K. Nørskov, J. Rossmeisl, A. Logadottir, L. Lindqvist, J. R. Kitchin, T. Bligaard and H. Jónsson, Origin of the Overpotential for Oxygen Reduction at a Fuel-Cell Cathode. *J*. *Phys*. *Chem*. *B* 2004, **108**, 17886.

8. A. A. Peterson, F. Abild-Pedersen, F. Studt, J. Rossmeisl and J. K. Nørskov, How copper catalyzes the electroreduction of carbon dioxide into hydrocarbon fuels. *Energy Environ*. *Sci*. 2010, **3**, 1311.

9. T. Liu, Z. Pan, J. J. M. Vequizo, K. Kato, B. Wu, A. Yamakata, K. Katayama, B. Chen, C. Chu and K. Domen, Overall photosynthesis of H_2_O_2_ by an inorganic semiconductor. *Nat*. *Commun*. 2022, **13**, 1034.

10. G.-h. Moon, W. Kim, A. D. Bokare, N.-e. Sung and W. Choi, Solar production of H_2_O_2_ on reduced graphene oxide–TiO_2_ hybrid photocatalysts consisting of earth-abundant elements only. [*Energy Environ*. *Sci*.](https://www.x-mol.com/paper/0/18) 2014, **7**, 4023.

11. M. Sun, X. Wang, Y. Li, H. Pan, M. Murugananthan, Y. Han, J. Wu, M. Zhang, Y. Zhang and Z. Kang, Bifunctional Pd-O_x_ Center at the Liquid–Solid–Gas Triphase Interface for H_2_O_2_ Photosynthesis. *ACS Catal*. 2022, **12**, 2138.

12. C. Pan, G. Bian, Y. Zhang, Y. Lou, Y. Zhang, Y. Dong, J. Xu and Y. Zhu, Efficient and stable H_2_O_2_ production from H_2_O and O_2_ on BiPO_4_ photocatalyst. *Appl. Catal. B Environ. Energy.* 2022, **316**, 121675.

13. C. Lai, M. Xu, F. Xu, B. Li, D. Ma, Y. Li, L. Li, M. Zhang, D. Huang, L. Tang, S. Liu, H. Yan, X. Zhou, Y. Fu and H. Yi, An S-scheme CdS/K_2_Ta_2_O_6_ heterojunction photocatalyst for production of H_2_O_2_ from water and air. *Chem*. *Eng*. *J*. 2023, **452**, 139070.

14. M. Gu, Y. Yang, L. Zhang, B. Zhu, G. Liang and J. Yu, Efficient sacrificial-agent-free solar H_2_O_2_ production over all-inorganic S-scheme composites. *Appl. Catal. B Environ. Energy.* 2023, **324**, 122227.

15. H. Peng, H. Yang, J. Han, X. Liu, D. Su, T. Yang, S. Liu, C.-W. Pao, Z. Hu, Q. Zhang, Y. Xu, H. Geng and X. Huang, Defective ZnIn_2_S_4_ Nanosheets for Visible-Light and Sacrificial-Agent-Free H_2_O_2_ Photosynthesis via O_2_/H_2_O Redox. *J. Am. Chem. Soc.* 2023, **145**, 27757.

16. Y. Zhang, L. Wang, H. Huang, C. Hu, X. Zhang, C. Wang and Y. Zhang, Water flow induced piezoelectric polarization and sulfur vacancy boosting photocatalytic hydrogen peroxide evolution of cadmium sulfide nanorods. *Appl. Catal. B Environ. Energy.* 2023, **331**, 122714.

17. H. Chen, S. Gao, G. Huang, Q. Chen, Y. Gao and J. Bi, Built-in electric field mediated S-scheme charge migration in COF/In_2_S_3_ heterojunction for boosting H_2_O_2_ photosynthesis and sterilization. *Appl. Catal. B Environ. Energy.* 2024, **343**, 123545.

18. E. Zhang, Q. Zhu, J. Huang, J. Liu, G. Tan, C. Sun, T. Li, S. Liu, Y. Li, H. Wang, X. Wan, Z. Wen, F. Fan, J. Zhang and K. Ariga, Visually resolving the direct Z-scheme heterojunction in CdS@ZnIn_2_S_4_ hollow cubes for photocatalytic evolution of H_2_ and H_2_O_2_ from pure water. *Appl. Catal. B Environ. Energy.* 2021, **293**, 120213.

19. T. Yang, D. Zhang, A. Kong, Y. Zou, L. Yuan, C. Liu, S. Luo, G. Wei and C. Yu, Robust Covalent Organic Framework Photocatalysts for H_2_O_2_ Production: Linkage Position Matters. *Angew. Chem. Int. Ed.* 2024, **63**, e202404077.

20. Q. Wu, J. Cao, X. Wang, Y. Liu, Y. Zhao, H. Wang, Y. Liu, H. Huang, F. Liao, M. Shao and Z. Kang, A metal-free photocatalyst for highly efficient hydrogen peroxide photoproduction in real seawater. *Nat. Commun.* 2021, **12**, 483.

21. J.-Y. Yue, L.-P. Song, Y.-F. Fan, Z.-X. Pan, P. Yang, Y. Ma, Q. Xu and B. Tang, Thiophene-Containing Covalent Organic Frameworks for Overall Photocatalytic H_2_O_2_ Synthesis in Water and Seawater. *Angew. Chem. Int. Ed.* 2023, **62**, e202309624.

22. Y. Zhang, C. Pan, G. Bian, J. Xu, Y. Dong, Y. Zhang, Y. Lou, W. Liu and Y. Zhu, H_2_O_2_ generation from O_2_ and H_2_O on a near-infrared absorbing porphyrin supramolecular photocatalyst. *Nat*. *Energy*. 2023, **8**, 361.

23. X. Zhang, H. Su, P. Cui, Y. Cao, Z. Teng, Q. Zhang, Y. Wang, Y. Feng, R. Feng, J. Hou, X. Zhou, P. Ma, H. Hu, K. Wang, C. Wang, L. Gan, Y. Zhao, Q. Liu, T. Zhang and K. Zheng, Developing Ni single-atom sites in carbon nitride for efficient photocatalytic H_2_O_2_ production. *Nat. Commun.* 2023, **14**, 7115.

24. J.-Y. Yue, L.-P. Song, Z.-X. Pan, P. Yang, Y. Ma, Q. Xu and B. Tang, Regulating the H_2_O_2_ Photosynthetic Activity of Covalent Organic Frameworks through Linkage Orientation. *ACS Catal*. 2024, **14**, 4728.

25. Y. Hou, P. Zhou, F. Liu, Y. Lu, H. Tan, Z. Li, M. Tong and J. Ni, Efficient Photosynthesis of Hydrogen Peroxide by Cyano-Containing Covalent Organic Frameworks from Water, Air and Sunlight. *Angew. Chem. Int. Ed.* 2024, **63**, e202318562.

26. J. Zhang, H. Lei, Z. Li, F. Jiang, L. Chen and M. Hong, Halogen-Modulated 2D Coordination Polymers for Efficient Hydrogen Peroxide Photosynthesis under Air and Pure Water Conditions. *Angew. Chem. Int. Ed.* 2024, **63**, e202316998.

27. C. Zhu, Y. Yao, Q. Fang, S. Song, B. Chen and Y. Shen, Unveiling the Dynamic Evolution of Single-Atom Co Sites in Covalent Triazine Frameworks for Enhanced H_2_O_2_ Photosynthesis. *ACS Catal*. 2024, **14**, 2847.

28. X. Wang, Y. Jin, N. Li, H. Zhang, X. Liu, X. Yang, H. Pan, T. Wang, K. Wang, D. Qi and J. Jiang, 12 Connecting Sites Linked Three-dimensional Covalent Organic Frameworks with Intrinsic Non-interpenetrated shp Topology for Photocatalytic H_2_O_2_ Synthesis. *Angew. Chem. Int. Ed.* 2024, **63**, e202401014.
